# Genetic susceptibility to gestational diabetes and its mild modification by bisphenol A and thyroid-stimulating hormone: findings from a South Chinese pregnancy cohort

**DOI:** 10.3389/fnut.2025.1652265

**Published:** 2025-12-10

**Authors:** Yanyan Mao, Zhaofeng Zhang, Yupei Shen, Min Li, Xiaoping Fei, Difei Wang, Qianxi Zhu, Xiaohong Chen, Jing Du

**Affiliations:** 1 Key Laboratory of Maternal & Fetal Medicine of National Health Commission of China, Shandong Provincial Maternal and Child Health Care Hospital Affiliated to Qingdao University, Jinan, China; 2 Shanghai-MOST Key Laboratory of Health and Disease Genomics, NHC Key Lab of Reproduction Regulation, Shanghai Institute for Biomedical and Pharmaceutical Technologies, Shanghai, China; 3 Obstetrics Department, The First People’s Hospital of Kunshan, Kunshan, Jiangsu, China; 4 Department of Obstetrics and Gynecology, Maternal and Child Health Hospital of Pudong New Area, Shanghai, China; 5 Key Laboratory of Research on Clinical Molecular Diagnosis for High Incidence Diseases in Western Guangxi of Guangxi Higher Education Institutions, Reproductive Medicine of Guangxi Medical and Health Key Discipline Construction Project, Affiliated Hospital of Youjiang Medical University for Nationalities, Baise, China; 6 Industrial College of Biomedicine and Health Industry, Youjiang Medical University for Nationalities, Baise, China

**Keywords:** gestational diabetes mellitus, genetic susceptibility, single-nucleotide variants, bisphenol A, thyroid-stimulating hormone, marginal effect analysis, modification

## Abstract

**Background:**

Gestational diabetes mellitus (GDM) is a multifaceted and complex condition. Genetic factors, maternal exposure to bisphenol A (BPA), and thyroid-stimulating hormone (TSH) levels have been associated with GDM. However, existing findings are inconsistent, and evidence regarding their interactions remains limited. This study aimed to identify single-nucleotide variants (SNVs) associated with GDM and to examine whether the genetic influence on GDM would be modulated by maternal BPA and TSH levels during pregnancy.

**Methods:**

This case–control study was nested within a prospective cohort of 2,884 pregnant women in South China from July 2016 to December 2020. Significant SNVs between cases and controls were identified by whole-exome sequencing and validated by Sequenom MassARRAY. Functional and pathway enrichment analyses were applied to explore potential biological pathways. The relationship between GDM and maternal SNVs’ genotype, BPA, and TSH was evaluated by logistic regression models and marginal effect analyses.

**Results:**

We identified 308 missense variants among 1,770 SNVs linked to GDM. After validation, the allele frequencies of PPARGC1A rs8192678 C > T (*p* = 0.005, FDR = 0.077) and GCK rs2971672 A > C (*p* = 0.007, FDR = 0.077) showed significant differences between cases and controls. In an exploratory analysis using logistical regression, the odds ratio (OR) for GDM was 0.417 (95% CI: 0.225–0.774) among women with the TT genotype of PPARGC1A rs8192678 and 0.470 (95% CI: 0.262–0.846) among those with the CC genotype of GCK rs2971672 compared to the wild type. Sub-population analysis revealed that urinary BPA levels were linked to an increased risk of GDM, with an OR of 2.295 (95% CI: 1.361–3.867). The protective effect ofPPARGC1A rs8192678 in GDM was confirmed and was non-linearly modified by sqrt-BPA levels. Additionally, this effect was modified by sqrt-TSH in a dose-dependent manner. The protective association was strongest at moderate BPA exposure levels (e.g., at sqrt-BPA = 2 and 3, the dy/dx for CT + TT vs. CC was −0.20 and −0.194, respectively; *p* < 0.01). At the highest level of BPA or TSH, the protective genetic effect was attenuated and became statistically non-significant.

**Conclusion:**

The study highlights the associations between GDM and the missense variant of PPARGC1A rs8192678, further revealing that the genetic effect is modified slightly by urinary BPA and serum TSH levels. The modification displayed a quasi-U-shaped distribution in relation to BPA and decreased as TSH levels increased.

## 1 Introduction

Gestational diabetes mellitus (GDM) is a prevalent metabolic complication among pregnant women, affecting a significant proportion of pregnancies worldwide. The global prevalence rate is approximately 14.0% (1), and it is specifically 14.7% in the Western Pacific region, including China (1). This condition is known to disrupt various metabolic pathways in pregnant women, resulting in abnormal placental modifications that can negatively affect both short- and long-term health outcomes for both the mother and the child (2–5). GDM also elevates the risk of complications, such as spontaneous abortion, gestational (6) hypertension, polyhydramnios, macrosomia, and neonatal hypoglycemia. Furthermore, it predisposes both mothers and their offspring to lifelong health challenges, such as diabetes, metabolic syndromes, and cardiovascular diseases (7).

The etiology of GDM is complex and multi-factorial, with advanced maternal age, obesity, and a history of adverse obstetrical outcomes being prominent risk factors. Previous studies have indicated a correlation between elevated serum triglyceride (TG) levels early in pregnancy and GDM (8, 9). Elevated thyroid-stimulating hormone (TSH) levels during early pregnancy have been linked to a higher risk of GDM (10). Moreover, the potential role of endocrine-disrupting chemicals (EDCs), particularly bisphenol A (BPA), in the pathogenesis of gestational diabetes mellitus (GDM) has attracted attention, given the concurrent rise in GDM incidence and BPA usage. This chemical, which is a widespread environmental contaminant found in food packaging, thermal receipts, and various consumer products, has been associated with GDM, although study findings have been inconsistent (11).

Beyond environmental factors, individual genetic profiles significantly influence GDM development. A multitude of single-nucleotide polymorphisms (SNPs) associated with GDM have been identified through candidate gene and genome-wide association studies, many of which are linked to type 2 diabetes (T2D) genes (12–15). Peroxisome proliferator-activated receptor-γ coactivator-1α (PPARGC1A/PGC-1α) is a coactivator of peroxisome proliferator-activated receptor-γ (PPARγ). Its expression products can regulate mitochondrial biogenesis, fatty acid metabolism, and insulin sensitivity (16). Glucokinase (GCK) encodes the key enzyme of the hexokinase family (hexokinase IV), which catalyzes the first step of glycolysis in liver and pancreatic islet beta cells (17). This enzyme regulates insulin secretion by sensing changes in glucose concentration. The missense variation at the 1444th locus (rs8192678) in exon 8 of PPARGC1A has attracted significant attention. However, epidemiological studies have produced inconsistent findings regarding its association with the risk of T2D (18–21). *In vitro* studies suggest that this polymorphism may impact PGC-1α stability, thereby influencing glucose and fat metabolism (22). Despite speculation, no significant association between rs8192678 and gestational diabetes mellitus has been observed in Scandinavian, Austrian, or Italian populations (23–25). Similar research in Chinese women remains limited.

Furthermore, elevated studies suggest that BPA exposure during pregnancy may influence glucose metabolism in both mothers and their children (26). However, the potential modification of genetic effects on GDM by BPA exposure or endocrine biomarkers, such as TSH, during pregnancy remains understudied. To address these knowledge gaps, we conducted a nested case–control study embedded within a prospective cohort of Chinese pregnant women. This study aimed to validate genetic variants identified through whole exome sequencing (WES) and prior research, examined the association between these variants, early gestational BPA exposure, TSH levels, and GDM, and investigated whether the genetic impact on GDM was modified by BPA and TSH levels during pregnancy.

## 2 Materials and methods

### 2.1 Study population and sample collection

This case–control study was nested within a pregnancy cohort of 2,884 women, including 329 with gestational diabetes mellitus (GDM) and 2,555 healthy women, recruited from hospitals in Pudong, Kunshan, and Changshou in South China between July 2016 and December 2020. In early pregnancy (the average gestation: 13.00 weeks), the clinical characteristics of pregnant women and their urine and blood samples were collected. Information on basic characteristics, such as serum TSH and TG levels during pregnancy, was obtained based on electronic medical records.

GDM was diagnosed according to the International Association of Diabetes and Pregnancy Study Groups (IADPSG) criteria, which include fasting blood glucose (FBG) > 5.1 mmol/L, 1-h oral glucose tolerance test (OGTT) > 10 mmol/L, or 2-h OGTT >8.5 mmol/L, conducted between the 24th and 28th weeks of gestation. Women exceeding any OGTT threshold were classified into the GDM group, while those with no history of GDM or diabetes and normal glucose tolerance were classified into the healthy group. Women with severe cardiovascular diseases, pre-existing diabetes, or other significant medical complications were excluded. For the study, 195 women with GDM and 180 healthy women were randomly selected. Maternal blood samples (*N* = 375) were collected from three hospitals during the first or second trimester, and maternal urine samples (*N* = 155) were collected during the first trimester at the Pudong hospital. All samples were initially stored at −20 °C and then transferred to a biobank for storage at −80 °C until analysis. The flowchart of the study population is presented in Supplementary Figure S1.

The study was approved by the Ethics Committee of the Shanghai Institute for Biomedical and Pharmaceutical Technologies on March 1, 2023 (Approval Number: PJ2023-10). All participants provided written informed consent in accordance with the Declaration of Helsinki.

### 2.2 Whole-exome sequencing

Ten whole blood samples from 195 cases and 10 matched samples from 180 controls were selected for whole-exome sequencing (WES). Sample preparation and WES data analysis were conducted using the standard pipeline (Supplementary Methods and Results).

### 2.3 Identification of rare, potentially pathogenic variants

Potentially pathogenic variants (*p* < 0.05) were classified based on damaging or probably damaging predictions and minor allele frequencies from three public databases (Supplementary material 1). Variants were categorized into four levels: High, Likely High, Medium, and Low.

### 2.4 Candidate SNP selection

Based on the whole-exome association study, variants were selected for further analysis according to the following criteria: Statistical significance (*p* < 0.05); preference for variants within exonic regions while filtering out intronic variants; preference for protein-altering (missense) variants within gene exonic regions; and variants predicted to be damaging or probably damaging by tools such as SIFT,^1^ Polyphen-2,^2^ MutationAssessor, MutationTaster,^3^ LRT, FATHMM, fathmm-MKL, PROVEAN,^4^ MetaSVM, MetaLR, M-CAP, and Combined Annotation Dependent Depletion.^5^

Meanwhile, previously published variants associated with T2D, glucose, lipid, or BPA metabolism were selected as candidate SNPs (27).

### 2.5 SNPs genotyping

Following selection, 63 single-nucleotide variations (SNVs) were chosen for MassARRAY genotyping, comprising 26 SNVs identified through WES and 37 SNVs reported in the literature. Paired primers for part of these SNVs were designed as detailed in Supplementary Table S1. These primers were designed by a multiplex PCR platform (iPlex GOLD Training Primer Set (36plex)), avoiding homology identification and ensuring its specificity. All selected SNVs were successfully genotyped, with an average call rate of ≥97%, indicating a genotyping failure rate of below 5%. All 375 selected blood samples (195 cases and 180 controls) were included in the validation process, which involved 20 samples used for whole-exome sequencing.

### 2.6 Assessment of urinary BPA concentration

Urinary total BPA concentration was quantified using an HPLC-MS/MS analytical method as previously described (28). Frozen urine samples were first thawed at room temperature. One milliliter of the dissolved sample, along with a mixed internal standard solution, was treated with β-glucuronidase and hydrolyzed in a water bath at 37 °C overnight. Following hydrolysis, the samples were extracted three times with 1 mL of a mixture of ethyl acetate and methyl tert-butyl ether. The supernatants were collected, evaporated under nitrogen flow, and the residues were dissolved in acetonitrile/water (6:4, v/v, 200 μL) before filtration. The BPA concentration was analyzed using Ultra High Performance Liquid Chromatography coupled with Triple Quadrupole Mass Spectrometry, following the method described in a previous study (29), with a limit of detection of 0.01 ng/mL.

Data on specific gravity (SG) were gathered for each urinary sample utilized in the assessment of BPA concentration. We calculated specific gravity (SG)-standardized BPA concentrations using the following formula: P_c_ = Pi[(SG_m_ - 1)/(SG_i_ - 1)], where P_c_ is the SG-standardized BPA concentration, P_i_ is the observed BPA concentration, SG_i_ is the specific gravity of the ith urine sample, and SG_m_ is the median SG for cases or controls (30, 31).

### 2.7 Statistical analysis

In the WES screening stage, Fisher’s exact test was employed to evaluate the significance of variants between 10 cases and 10 controls, with a cut-off criterion of *p* < 0.05 for statistical significance. The raw WES data were deposited in the Sequence Read Archive (PRJNA719775). Functional and pathway enrichment analyses of genes associated with significant SNVs were visualized using [Metascape]^6^ (32). For the validation stage, the *χ*^2^ test was used to compare differences in allele and genotype frequencies between cases and controls, utilizing SHEsisPlus (33, 34). Statistical significance was set at *p* < 0.05.

All genetic associations and interaction terms underwent Benjamini–Hochberg FDR correction. Significance was defined as *q* < 0.10.

Urinary BPA concentrations and serum TSH levels were transformed using the square root method to approximate a normal distribution. The histogram and Q-Q plots were analyzed for the transformed data (Supplementary Figure S2). BPA concentrations and TSH levels were further categorized into high and low groups by the median (BPA: ≥4.9 vs. <4.9 ng/mL; TSH: ≥0.91 vs. <0.91 mIU/L). Serum TG levels were categorized into high and low groups (≥1.7 mmol/L vs. <1.7 mmol/L). Continuous variables were presented as means ± standard deviation (SD), while categorical variables were presented as frequencies (percentages).

Differences between the case and control groups were assessed using a univariate logistic regression for both continuous and categorical variables. A multi-variable logistic regression analysis was conducted to investigate the relationship between GDM and the genotypes of significant SNVs, considering both heterozygote and homozygote models, as well as dominant and recessive models. Marginal effect analysis was further employed to determine if the genetic impact of rs8192678 on GDM was modulated by urinary BPA and serum TSH levels (35). Estimates were validated using the bootstrap command in Stata 15.1.

In sensitivity analyses, GDM sub-types were identified: those with FBG > 5.1 mmol/L but with both 1-h (≤10 mmol/L) and 2-h OGTT (≤8.5 mmol/L) within normal limits were classified as the “only FBG-high” group, while those with FBG ≤ 5.1 mmol/L but with 1-h (>10 mmol/L) or 2-h OGTT (>8.5 mmol/L) exceeding normal limits were classified as the “only OGTT-high” group. The relationship between the GDM sub-types and the two significant SNVs was further examined. In the sub-population, we calculated ORs using categorical BPA and TSH to improve their clinical interpretability.

Logistic regression and marginal effect analyses were conducted using Stata 15.1 (Stata Corp, TX, USA). Fisher’s Exact test calculations and all graphical representations were generated using R version 4.3.1. Missing values (3.2%) for maternal age were imputed using the average age of case or control women, respectively. The power was calculated to compare differences in allele and genotype frequencies between cases and controls, as well as for multiple regression (Supplementary material 1).

### 2.8 Prediction of protein structure and phase separation for PPARGC1A

The structural changes in PPARGC1A were predicted using the online tools HOPE and SWISS-MODEL. The alteration modes of proteins for the significant gene were visualized using PyMOL. The phase separation (PS) of the protein was predicted using PhaSePred (predict.phasep.pro) (more information listed in Supplementary material 1).

## 3 Results

### 3.1 Overview of case and control women

Maternal characteristics of GDM cases and controls are summarized in Table 1. The average age was 30.47 years for cases and 29.02 years for controls. The majority of the women did not have chronic diseases (cases: 90.77%; controls: 90.56%). Among the 195 cases, 54.87% accounted for only OGTT values exceeding the cut-point, while 23.08% due to only FBG > 5.1 mmol/L.

**Table 1:** Maternal age, chronic diseases, and glucose level between GDM cases and controls.

Variables	Cases (*n* = 195)	Controls (*n* = 180)		*Ζ*/*χ*^2^	*p*
Age (Years, Mean, SD)	30.47 ± 0.34	29.02 ± 0.32		−14.0779	<0.001
Chronic diseases (*n* (%))				0.0051	0.943
Yes	18 (9.23)	17 (9.44)			
No	177 (90.77)	163 (90.56)			
Site (*n*, %)				2.7575	0.252
Pudong	92 (47.18)	98 (54.44)			
Kunshan	90 (46.15)	75 (41.67)			
Changshou	13 (6.67)	7 (3.89)			
Sub-types for GDM (*n*, %)					
Type 1: Only FBG > 5.1 mmol/L	45 (23.08)	–	–	–	–
Type 2: Only (1 h OGTT > 10 mmol/L or 2 h OGTT > 8.5 mmol/L)	107 (54.87)	–	–	–	–

GDM, gestational diabetes mellitus; SD, standard deviation; FBG, fasting blood glucose; OGTT, oral glucose tolerance test.

### 3.2 Identification of rare, potentially pathogenic variants

The significant variants were classified depending on damaging or probably damaging prediction combined with the minor allele frequencies in three public databases. Four risk groups were identified: High, Likely-high, Medium, and Low (Supplementary Table S2).

### 3.3 Whole-exome association study

In total, WES identified 179,805 alteration sites within 10 cases and 10 controls selected from a study population of 375 women. Among these, 1,770 significant SNVs were screened between cases and controls with a significance threshold of *p* < 0.05 (Supplementary Table S3), and about 67% of the variants were highly stable by validation (Supplementary Figure S6).

**Figure 1:**
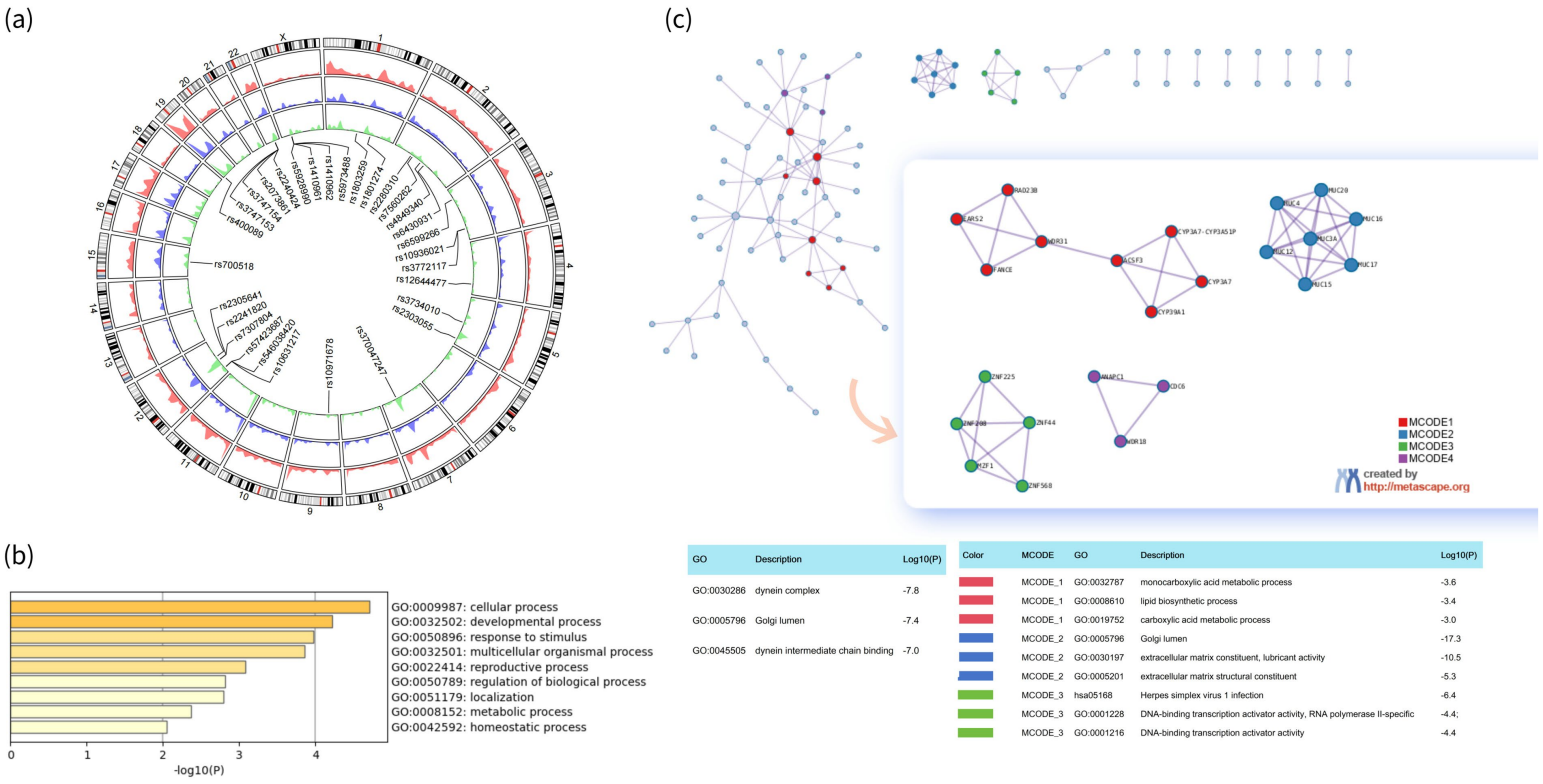
Analyses based on the whole-exome sequencing (WES). **(a)** Circus plot of the 1,79,805 variants detected by WES in cases and controls (including 1,79,805 sites in red, 1,770 significant variants in purple, 713 significant exonic variants in green, and the top 30 variants with the smallest *p*-values); **(b)** Heat-map of biology process cluster in functional enrichment analysis for the significant different SNVs related genes based on whole-exome sequencing; **(c)** Protein–protein interaction network and MCODE components identified in the gene lists of 246 genes for 308 differential missense variants.

Among the 1,770 significant sites, 713 sites were in the exonic region, with 642 found in at least one case. The top 15 exonic variants included HOXC9 (rs2241820; *p* = 1.19 × 10^−4^), LIMK2 (rs3747153; *p* = 7.14 × 10^−4^), LIMK2 (rs3747154; *p* = 7.14 × 10^−4^), PATZ1 (rs2240424; *p* = 7.14 × 10^−4^), FCGR2A (rs1801274; *p* = 7.14 × 10^−4^), ARHGAP25 (rs2280310; *p* = 7.14 × 10^−4^), YP19A1 (rs700518; *p* = 1.09 × 10^−3^), CPSF6 (rs2305641; *p* = 1.31 × 10^−3^), GCFC2 (rs7560262; *p* = 1.58 × 10^−3^), MAGEB16 (rs1410961; *p* = 1.91 × 10^−3^), MAGEB16 (rs1410962; *p* = 1.91 × 10^−3^), MAGEB16 (rs5973488; *p* = 1.91 × 10^−3^), MAGEB16 (rs4829390; *p* = 1.91 × 10^−3^), MAGEB16 (rs4829391; *p* = 1.91 × 10^−3^), and MAGEB16 (rs4829392; *p* = 1.91 × 10^−3^). Six of these sites reached the suggestive significance threshold (1 × 10^−4^). A circus plot showing the associations between all 179,805 variants, including 1,770 significant variants, 713 exonic variants, and the top 30 variants with the smallest *p*-values, is presented in Figure 1a.

Among 713 exonic variants, 308 were missense variants, appearing in eight or nine cases and annotated in 246 genes (Supplementary Table S4). These genes were mainly enriched in nine biological processes: cellular process, developmental process, response to stimulus, multicellular organismal process, reproductive process, regulation of biological process, localization, metabolic process, and homeostatic process (Supplementary Table S5 and Figure 1b).

The Molecular Complex Detection (MCODE) networks for individual gene lists are provided in Figure 1c. Pathway and process enrichment analysis for each MCODE component yielded the top three functional descriptions by *p*-value. The top processes across all components included dynein complex, Golgi lumen, and dynein intermediate chain binding. Specifically, MCODE_1 was associated with monocarboxylic acid metabolic process, lipid biosynthetic process, and carboxylic acid metabolic process. MCODE_2 highlighted the Golgi lumen, an extracellular matrix constituent, and lubricant activity. MCODE_3 focused on Herpes simplex virus 1 infection, DNA-binding transcription activator activity, and RNA- and DNA-binding transcription activator activity.

### 3.4 Validation the candidate SNVs associated with GDM

Out of the 63 selected SNVs, 31 significant missense exonic SNVs were successfully genotyped (Supplementary Table S6). Genotype distributions were in Hardy–Weinberg equilibrium (*p* > 0.05). Comparing 195 GDM cases with 180 controls, PPARGC1A rs8192678 and GCK rs2971672 were significantly associated with GDM. The allele and genotype frequencies of PPARGC1A rs8192678 C > T (P_allele_ = 0.005, P_genotype_ = 0.020) and GCK rs2971672 A > C (P_allele_ = 0.007, P_genotype_ = 0.029) showed significant differences between the cases and controls (Table 2). In examining allele and genotype frequencies of the significant SNVs between GDM sub-types and controls, GCK rs2971672 A > C showed significant differences between only OGTT cases and controls (Supplementary Table S7). Other SNVs did not show significant associations with GDM (Supplementary Table S8). In the analysis of gene interactions, the PPARGC1A variant rs8192678 C > T showed a significant interaction with glucose-6-phosphatase catalytic subunit 2 (G6PC2) variant rs16856187 A > C (*p* = 0.037) on GDM. Additionally, the variant rs2971672 A > C interacted significantly with glucose-6-phosphate isomerase (GPI) variant rs8191371 T > C (*p* = 0.006) (Supplementary Table S9). After FDR correction, the rs8192678 T allele and GCK rs2971672 C allele’s effect remained significantly protective against GDM (*q* = 0.077). Their genotype and interactions’ effect on GDM did not meet significance thresholds (*q* > 0.1).

**Table 2:** Distribution results of SHEsisPlus analysis between GDM cases and controls.

SNP	Allele association						Genotype association						Hardy–Weinberg equilibrium	
	Chi^2^	Pearson’s *p*	Fisher’s *p*	OR (95% CI)	Allele counts (frequency)		Chi^2^	Pearson’s *p*	Fisher’s *p*	Genotype observed (expected) counts			Chi^2^	*p*
GCK rs2971672 (A > C)	7.509	0.006	0.007	0.665 (0.496 ~ 0.89)	A	C	7.039	0.029	0.029	AA	AC	CC		
Case					238 (0.62)	148 (0.38)				77 (72,35)	84 (92.57)	32 (28.98)	1.128	0.569
Control					184 (0.52)	172 (0.48)				50 (47.54)	84 (88.86)	44 (41.55)	0.538	0.463
PPARGC1A rs8192678 (C > T)	7.891	0.004	0.005	0.658 (0.491 ~ 0.881)	C	T	7.838	0.019	0.020	CC	CT	TT		
Case					242 (0.63)	144 (0.37)				76 (75.83)	90 (90.35)	27 (26.82)	0.138	0.933
Control					188 (0.53)	170 (0.47)				49 (49.35)	90 (89.25)	40 (40.36)	0.012	0.912

GDM, gestational diabetes mellitus; OR, odds ratio; CI, confidence interval.

Further exploratory analysis revealed that GDM was associated with a decreased risk in the presence of the TT genotype of PPARGC1A rs8192678 C > T (OR: 0.417; 95% CI: 0.225–0.774) and the CC genotype of GCK rs2971672 A > C (OR: 0.470; 95% CI: 0.262–0.846) compared to wild types. The association with the TT genotype of rs8192678 was consistent across both GDM sub-types, while the CC genotype of rs2971672 was associated with reduced risk only in cases with high OGTT (Table 3).

**Table 3:** Logistic regression analysis for GDM and the genotype of PPARGC1A rs8192678 and GCK rs297167.

SNP and Genotype	All GDM cases vs. Controls				Only FBG high cases VS Controls		Only OGTT high cases VS Controls	
	cOR_with_CI	*p*	aOR_with_CI ^a^	*p*	aOR_with_CI ^a^	*p*	aOR_with_CI ^a^	*p*
rs2971672 (A > C)								
AC vs. AA	0.649 (0.407–1.036)	0.070	0.697 (0.432–1.122)	0.137	0.758 (0.362–1.590)	0.464	0.557 (0.319–0.973)	0.040
CC vs. AA	0.472 (0.265–0.842)	0.011	0.471 (0.262–0.848)	0.012	0.442 (0.165–0.180)	0.103	0.461 (0.233–0.911)	0.026
AC + CC vs. AA	0.588 (0.387–0.910)	0.017	0.616 (0.396–0.960)	0.032	0.644 (0.322–1.290)	0.214	0.523 (0.313–0.873)	0.013
CC vs. AC + AA	0.605 (0.364–1.008)	0.054	0.578 (0.343–0.973)	0.039	0.518 (0.213–1.262)	0.148	0.633 (0.343–1.168)	0.143
rs8192678 (C > T)								
CT vs. CC	0.645 (0.406–1.024)	0.063	0.677 (0.422–1.090)	0.106	0.753 (0.365–1.554)	0.443	0.707 (0.406–1.232)	0.221
TT vs. CC	0.435 (0.237–0.798)	0.007	0.417 (0.225–0.775)	0.006	0.257 (0.078–0.844)	0.025	0.444 (0.212–0.931)	0.032
CT + TT vs. CC	0.580 (0.375–0.899)	0.015	0.593 (0.380–0.926)	0.022	0.596 (0.297–1.197)	0.146	0.624 (0.369–1.054)	0.078
TT vs. CT + CC	0.565 (0.330–0.968)	0.038	0.524 (0.302–0.910)	0.022	0.306 (0.101–0.926)	0.036	0.547 (0.282–1.061)	0.074

GDM, gestational diabetes mellitus; FBG, fasting blood glucose; OGTT, oral glucose tolerance test; cOR, crude odds ratio; aOR, adjusted odds ratio; CI, confidence interval.

^a ORs were adjusted for site, maternal age, and chronic diseases.^

### 3.5 Association between GDM and maternal PPARGC1A rs8192678, TG, TSH, and BPA exposure in early gestation

#### 3.5.1 Multi-variable logistic regression analysis of maternal PPARGC1A rs8192678, TG, TSH, BPA and GDM

In the sub-population of 155 pregnant women, the relationship between GDM and maternal BPA exposure was analyzed. Women with GDM exhibited lower TSH and TG levels and BPA concentrations compared to controls (Supplementary Table S11). The result suggests a potential association between elevated BPA exposure and GDM, along with alterations in lipid and thyroid profiles during early gestation.

In the multi-variable logistic regression analysis, in addition to PPARGC1A rs8192678, maternal urinary BPA concentration and serum TG and TSH levels during early gestation were associated with GDM. For each unit increase in the square root-transformed BPA (sqrt-BPA) level, the odds of GDM increased by more than two times (OR: 2.295; 95% CI: 1.361–3.867) in Model 1 (Table 4). Conversely, the odds of GDM decreased by 61.4% (OR: 0.386; 95% CI: 0.150, 0.992) for each unit increase in the square root-transformed TSH (sqrt-TSH) level. The OR for GDM was 3.071 (95% CI: 1.17, 8.061) when comparing women with TG levels of 1.7 mmol/L or higher to those with TG levels below 1.7 mmol/L. Sensitivity analysis results were consistent when using categorical BPA and TSH instead of continuous variables. These associations remained significant after regrouping the genotype in Models 2 and 3 (Table 4, dominant and recessive models). No significant interaction items were observed in the analyses (Supplementary Table S12).

**Table 4:** Logistic regression analysis for the genotype of PPARGC1A rs8192678 C > T, early-gestational BPA exposure, and GDM.

Primary analysis					Sensitivity analysis				
Variables	*β*	*Z*	aOR_with_CI ^a^	*p*	Variables	*β*	*Z*	aOR_with_CI^a^	*p*
Model 1					Model 1				
rs8192678 (C > T): CT vs. CC	−0.946	−2.12	0.388 (0.162–0.932)	0.034	rs8192678 (C > T): CT vs. CC	−0.878	−2.03	0.416 (0.178–0.970)	0.042
rs8192678 (C > T): TT vs. CC	−1.223	−214	0.294 (0.096–0.903)	0.032	rs8192678 (C > T): TT vs. CC	−1.280	−2.24	0.278 (0.091–0.851)	0.025
SQRT (BPA)	0.831	3.12	2.295 (1.361–3.867)	0.002	BPA: ≥Median vs. < Median	0.872	2.24	2.392 (1.115–5.133)	0.025
SQRT (TSH)	−0.951	−1.98	0.386 (0.150–0.992)	0.048	TSH: ≥Median vs. <Median	−0.758	−1.98	0.469 (0.221–0.993)	0.048
TG: ≥1.7 vs. <1.7 mmol/L	1.122	2.28	3.071 (1.170–8.061)	0.023	TG: ≥1.7 vs. <1.7 mmol/L	1.032	2.19	2.807 (1.113–7.080)	0.029
Model 2					Model 2				
rs8192678 (C > T): CT/TT vs. CC	−1.028	−2.46	0.358 (0.158–0.812)	0.014	rs8192678 (C > T): CT/TT vs. CC	−0.988	−2.43	0.372 (0.167–0.827)	0.015
SQRT (BPA)	0.845	3.19	2.328 (1.385–3.913)	0.001	BPA: ≥Median vs. <Median	0.883	2.28	2.418 (1.130–5.173)	0.023
SQRT (TSH)	−0.962	−1.99	0.382 (0.148–0.984)	0.046	TSH: ≥Median vs. <Median	−0.773	−2.02	0.462 (0.218–0.976)	0.043
TG: ≥1.7 vs. <1.7 mmol/L	1.135	2.31	3.110 (1.187–8.150)	0.021	TG: ≥1.7 vs. <1.7 mmol/L	1.048	2.22	2.853 (1.133–7.188)	0.026
Model 3					Model 3				
rs8192678 (C > T): TT vs. CT/CC	−0.688	−1.36	0.502 (0.187–1.352)	0.173	rs8192678 (C > T): TT vs. CT/CC	−0.772	−1.53	0.462 (0.172–1.239)	0.125
SQRT (BPA)	0.768	2.98	2.156 (1.300–3.575)	0.003	BPA: ≥Median vs. <Median	0.816	2.15	2.261 (1.074–4.758)	0.032
SQRT (TSH)	−0.843	−1.81	0.430 (0.173–1.071)	0.070	TSH: ≥Median vs. <Median	−0.648	−1.74	0.523 (0.252–1.083)	0.081
TG: ≥1.7 vs. <1.7 mmol/L	1.015	2.12	2.759 (1.107–7.051)	0.034	TG: ≥1.7 vs. <1.7 mmol/L	0.949	2.06	2.583 (1.046–6.379)	0.040

GDM, gestational diabetes mellitus; aOR, adjusted odds ratio; CI, confidence interval; SQRT (BPA), bisphenol A concentration was transformed by square root; TG, triglyceride; SQRT (TSH), thyroid-stimulating hormone concentration was transformed by square root.

^aORs were adjusted for maternal age, body mass index, gravidity, and parity.^

#### 3.5.2 Average marginal effects (AMEs) of PPARGC1A rs8192678s and TG on GDM

**Figure 2:**
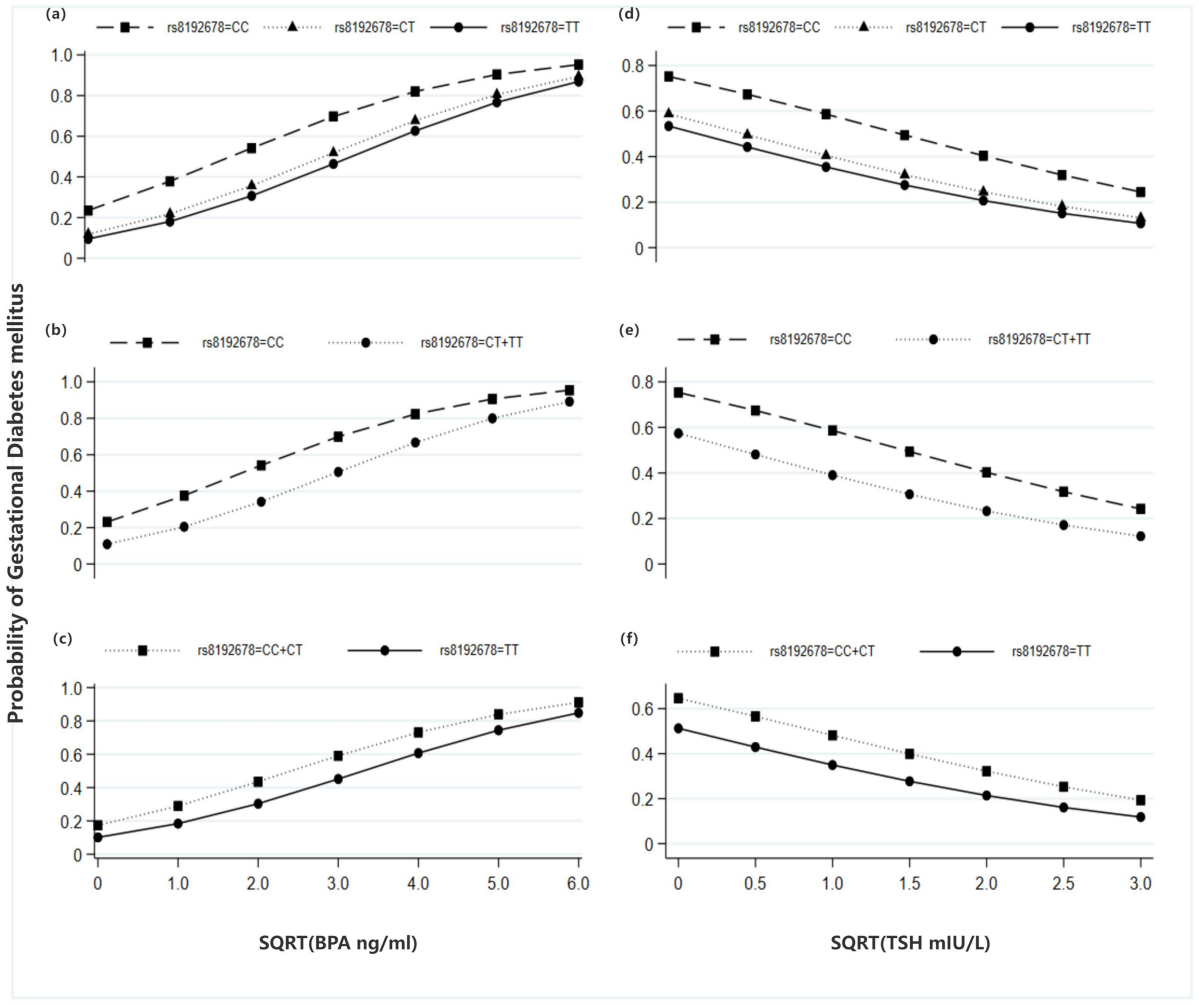
Predictive probabilities for GDM across genotypes of PPARGC1A rs8192678, based on the Model 1, 2 & 3 in Table 4 (Primary analysis). **(a)** GDM probability for rs8192678 genotypes (CC, CT, TT) at varying sqrt-BPA levels; **(b)** GDM probability for rs8192678 genotypes (CC, CT + TT) at varying sqrt-BPA levels; **(c)** GDM probability for rs8192678 genotypes (CC + CT, TT) at varying sqrt-BPA levels; **(d)** GDM probability for rs8192678 genotypes (CC, CT, TT) at varying sqrt-TSH levels; **(e)** GDM probability for rs8192678 genotypes (CC, CT + TT) at varying sqrt-TSH levels; **(f)** GDM probability for rs8192678 genotypes (CC + CT, TT) at varying sqrt-TSH levels.

The predictions and marginal effects were further analyzed using the margins command in Stata 15.1. The plots showed that the probability of GDM increased linearly with the sqrt-BPA level and decreased linearly with the sqrt-TSH level (Figure 2). This trend was observed in both the heterozygote and homozygote model (Model 1, Figures 2a,d), as well as in the dominant and recessive models (Model 2 and Model 3, Figures 2b,c,e,f).

The average marginal effects (AMEs) of PPARGC1A rs8192678 and elevated TG on GDM probability were quantified using multivariable logistic regression models, adjusted for maternal age, body mass index, gravidity, parity, BPA, and TSH levels. As shown in Supplementary Table S13, carriage of the T allele was associated with a reduced probability of GDM. Specifically, individuals with the CT genotype had an average 17.5 percentage-point reduction (dy/dx = −0.175, 95% CI: −0.330 to −0.021, *p* = 0.026) in the predicted probability of GDM compared to those with the CC genotype. The reduction was more pronounced for the TT genotype, with an average 22.4 percentage-point reduction (dy/dx = −0.224, 95% CI: −0.419 to −0.029, *p* = 0.024). When genotypes were combined (CT/TT vs. CC), the AME indicated a 19.0 percentage-point reduction in GDM probability (dy/dx = −0.190, 95% CI: −0.333 to −0.046, *p* = 0.009).

Elevated TG levels (≥1.7 mmol/L) were a strong, independent risk factor for GDM. After adjusting for covariates, having high TG was associated with an average 21.4 percentage-point increase (dy/dx = 0.214, 95% CI: 0.036–0.391, *p* = 0.018) in the predicted probability of GDM compared to lower TG levels (<1.7 mmol/L).

#### 3.5.3 Effect modification by BPA and TSH exposure levels

Marginal effect analysis at representative values was employed to assess whether the genetic effect of PPARGC1A rs8192678 was modified by maternal exposure to BPA and TSH. The results suggested a significant and dynamic effect modification.

**Figure 3:**
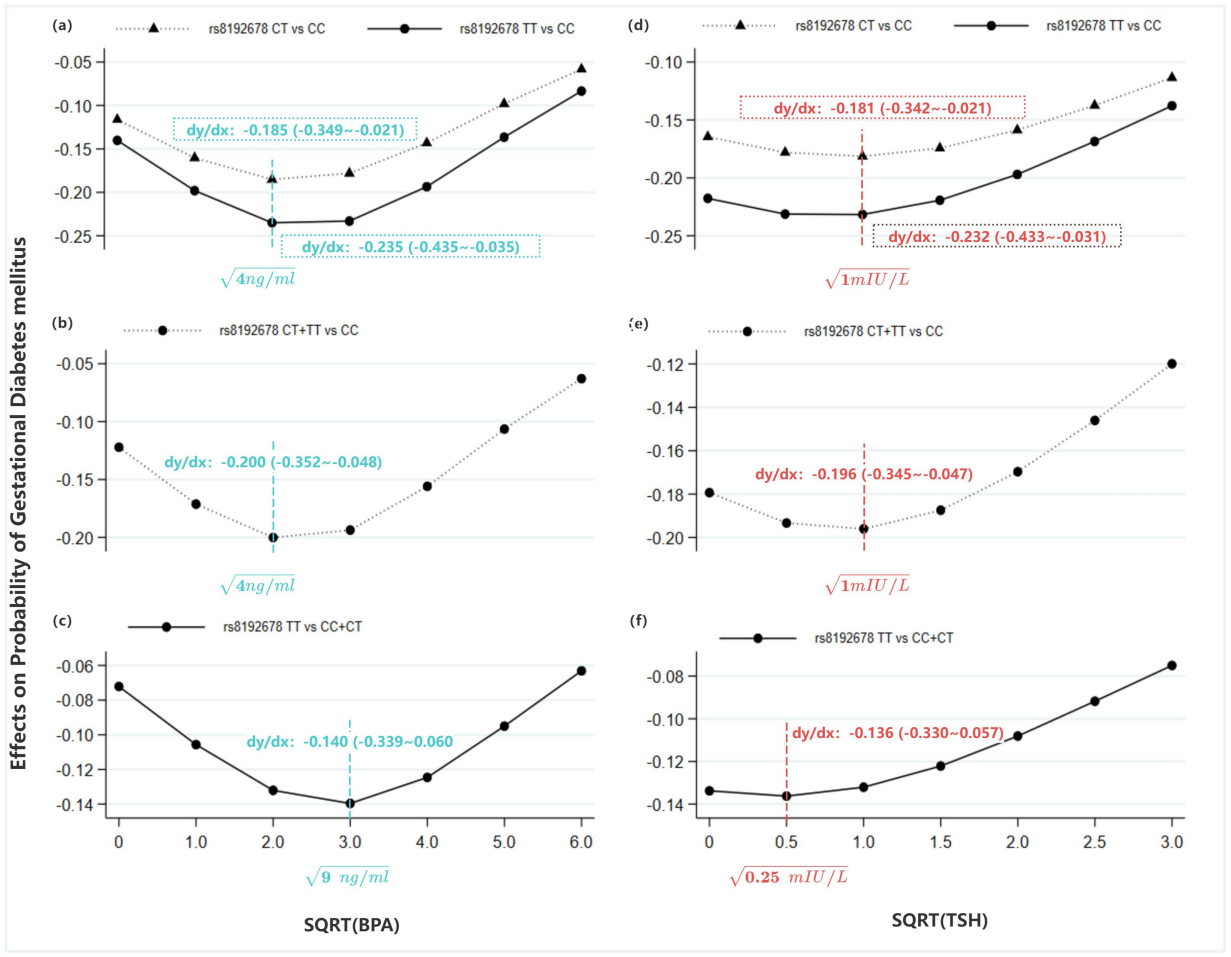
Average marginal effects (AMEs) on GDM probability, based on the Model 1, 2 & 3 in Table 4 (Primary analysis). **(a)** AMEs on GDM probability of CT and TT type for PPARGC1A rs8192678 compared to CC type at varying sqrt-BPA levels; **(b)** AMEs on GDM probability of CT + TT type for PPARGC1A rs8192678 compared to CC type at varying sqrt-BPA levels; **(c)** AMEs on GDM probability of TT type for PPARGC1A rs8192678 compared to CC + CT type at varying sqrt-BPA levels; **(d)** AMEs on GDM probability of CT and TT type for PPARGC1A rs8192678 compared to CC type at varying sqrt-TSH levels; **(e)** AMEs on GDM probability of CT + TT type for PPARGC1A rs8192678 compared to CC type at varying sqrt-TSH levels; **(f)** AMEs on GDM probability of TT type for PPARGC1A rs8192678 compared to CC + CT type at varying sqrt-TSH levels.

The protective effect of the PPARGC1A rs8192678 T allele was non-linearly modified by sqrt-BPA levels. The protective association was strongest at moderate BPA exposure levels (e.g., at sqrt-BPA = 2 and 3, the dy/dx for CT/TT vs. CC was −0.20 and −0.194, respectively; *p* < 0.01). Critically, at the highest level of BPA exposure (sqrt-BPA = 6), the protective genetic effect was attenuated and became statistically non-significant (dy/dx = −0.063, *p* = 0.179), indicating that high environmental BPA exposure may negate the protective genetic advantage (Figures 3a–c; Supplementary Table S14).

The protective effect of the T allele was also modified by sqrt-TSH in a dose-dependent manner. The effect was strong at lower TSH levels (e.g., at sqrt-TSH < =1, dy/dx for CT/TT vs. CC = −0.179–0.196, *p* < 0.01) and exhibited a gradual, linear attenuation as TSH levels increased. At the highest TSH level (sqrt-TSH = 3.0), the effect was weakened and lost statistical significance (dy/dx = −0.119, *p* = 0.090), suggesting that higher maternal TSH levels may diminish the protective genetic effect (Figures 3e–f; Supplementary Table S14).

**Figure 4:**
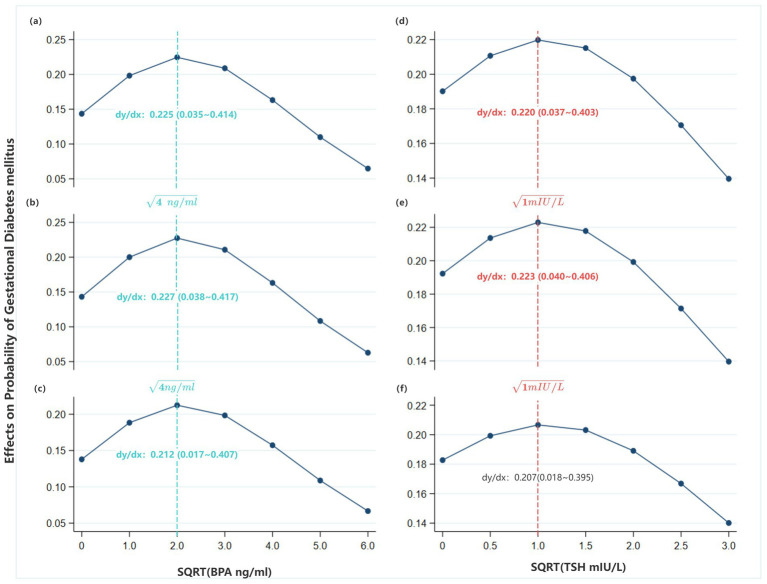
Average marginal effects (AMEs) on GDM probability of women with TG of 1.7 mmol/L or higher (compared to those with TG less than 1.7 mmol/L), based on the Model 1, 2 & 3 in Table 4 (Primary analysis). **(a)** AMEs on GDM probability at varying sqrt-BPA levels, based on Model 1; **(b)** AMEs on GDM probability at varying sqrt-BPA levels, based on Model 1; **(c)** AMEs on GDM probability at varying sqrt-BPA levels, based on Model 2; **(d)** AMEs on GDM probability at varying sqrt-TSH levels, based on Model 2; **(e)** AMEs on GDM probability at varying sqrt-TSH levels, based on Model 3; **(f)** AMEs on GDM probability at varying sqrt-TSH levels, based on Model 3.

The AME of high TG (≥1.7 mmol/L) on GDM risk remained positive and statistically significant across most strata of sqrt-BPA and sqrt-TSH levels, confirming its robust and independent association with GDM. The magnitude of the effect was greatest at moderate levels of environmental exposure (Figures 4a–f; Supplementary Table S15).

### 3.6 Molecular visualization of the protein coded by PPARGC1A

The schematic structure of the PPARGC1A protein highlighted the original and mutant amino acids (Supplementary Figure S4). The PPARGC1A rs8192678 C > T variant, being located at 4p15.2 (g.23815662 C > T c.1444 G > A p. Gly482Ser), introduced a missense mutation that changes an amino acid from glycine (Gly) to serine (Ser). According to predictions from online tools, this mutation occurred within a region annotated in UniProt as crucial for interaction with Ring Finger Protein 34 (RNF34). Glycine, being the most flexible amino acid, contributed to the flexibility required for proper protein function. The substitution of glycine with serine—an amino acid with different properties—could disrupt this flexibility and consequently impact the protein’s ability to interact with RNF34. The mutated residue (serine) was larger than the wild-type glycine, potentially causing steric hindrance or “bumps” in the protein structure. The change from glycine, which allowed a broader range of torsion angles due to its flexibility, to serine, which had more constrained angles, might force the local backbone into an incorrect conformation, disrupting the protein’s structural integrity. Moreover, this mutation could alter the length and stability of hydrogen bonds in the vicinity of the mutation site. These structural changes could further result in alteration of the protein’s function and its interaction with other molecules, affecting the overall biological role of PPARGC1A.

### 3.7 Prediction of phase separation for PPARGC1A

Phase separation (PS) of PPARGC1A was predicted using PhaSePred, as shown in Supplementary Figure S5. This tool integrates various predictors, such as the self-assembling phase-separating predictor (SaPS), the partner-dependent phase-separating predictor (PdPS), the granule-forming propensity predictor (catGRANULE), the prion-like domain predictor (PLAAC), π-contact predictor (PScore), IDR predictor (ESpritz), low-complexity region predictor (SEG), hydropathy prediction from CIDER (Hydropathy), coiled-coil domain predictor (DeepCoil), and immunofluorescence image-based droplet-forming propensity predictor (DeepPhase). The radar chart in Supplementary Figure S5a displays the proteome-level quantiles for these predictors. Most features, except for hydropathy and DeepPhase, had quantile values exceeding 0.75. These high quantile scores indicate a significant propensity for PPARGC1A to undergo phase separation, reflecting its potential role in forming phase-separated droplets or structures within cells.

## 4 Discussion

This nested case–control study underscores the potential roles of genetic variants in GDM and reveals an association between GDM and the missense variant of PPARGC1A rs8192678 and the variant of GCK rs2971672. Upon sub-population analysis, PPARGC1A rs8192678, urinary BPA concentration, and serum TG and TSH levels were related to GDM. The difference in GDM probability between the TT and CC genotype of PPARGC1A rs8192678 varied with changes in urinary BPA and serum TSH levels, following a U-shaped distribution for BPA and monotonic attenuation with increasing TSH levels.

Gestational diabetes mellitus (GDM) is a complex condition influenced by the interplay of genetic and environmental factors. Research has shown that GDM shares genetic characteristics with other forms of diabetes, such as type 1 diabetes (T1DM), type 2 diabetes (T2DM), and maturity-onset diabetes of the young (MODY). Numerous polymorphisms in genes associated with these diabetes types have been identified or confirmed to be linked to GDM through candidate gene studies, meta-analyses, and genome-wide association studies (GWAS) (36). This genetic overlap highlights the importance of understanding the underlying genetic predispositions when assessing the risk and management of GDM. In our study of 2,884 Chinese pregnant women, we identified 308 missense variants associated with GDM through WES. Further validation confirmed a significant association between the PPARGC1A rs8192678 (C > T) variant and GDM. The missense variant of PPARGC1A, located in the exonic region, has been linked to elevated BMI, higher total cholesterol levels, and increased fasting plasma glucose (37). It also appears to be associated with reduced insulin sensitivity and heightened insulin resistance, which can influence the development of T2DM (38, 39). However, we observed that T carriers were less likely to develop GDM. The T allele’s protective effect in GDM may arise from compensatory adaptation of the placenta during pregnancy. As far as we know, the variant may affect the differentiation of human white adipocytes, lipogenesis, and the content and turnover of PGC-1α, as indicated by recent findings (40). Previous research suggests that Gly482Ser missense polymorphism in PGC-1α has metabolic consequences on lipid metabolism that could influence insulin secretion (20), while lipid and lipid metabolism play an important role in diabetic complications (41).

Based on in silico structural predictions, the Gly482Ser variant may alter the residue’s flexibility, binding dynamics, protein size, and backbone conformation (42). Such alterations may affect the binding of PGC-1αwith RNF34, theoretically altering its ubiquitin-mediated degradation pathways and modifying oxidative stress and regulating lipid metabolism in brown fat cells, as well as affecting placental mitochondrial function during pregnancy (43, 44). Notably, further prediction indicated that the protein coded by PPARGC1A may undergo phase separation, with quantiles for both self-assembling and partner-dependent phase-separation predictors exceeding 0.8.

Although this mechanistic framework is supported by computational evidence showing altered binding interfaces and prior reports of RNF34-mediated PGC-1α regulation, it remains a hypothesis, requiring empirical validation in pregnancy-relevant models. This validation is necessary to confirm our predictions about the protein’s phase separation capability and to ascertain whether the mutation affects the protein’s structure and function in these models, as well as its potential impact on related biological processes, such as lipid and glucose metabolism.

While the interaction between the PPARGC1A variant rs8192678 and the G6PC2 variant rs16856187 on GDM was not significant, with an FDR greater than 0.1, this interaction may still be considered exploratory due to the limited power of our study to detect such interactions at FDR thresholds with the current sample size. G6PC2 encodes the islet-specific glucose-6-phosphatase catalytic subunit-related protein (IGRP) and is crucial for blood glucose regulation and diabetes pathophysiology (43, 45). As a member of the G6PC protein family, G6PC2 catalyzes the conversion of glucose 6-phosphate into glucose and phosphate. Overexpression of full-length G6PC2 could increase glucose-6-phosphatase activity (46). PGC-1α regulates hepatic gluconeogenesis by co-activating HNF4α or Foxo1, which are key transcription factors for G6PC (47, 48). The rs8192678 (Gly482Ser) variant may alter PGC-1α stability, potentially suppressing G6PC2 expression and hepatic glucose output. However, rs16856187 is a downstream variant for G6PC2 and unlikely to play a functional role, though it has been linked to T2DM (49, 50). It may tag functional variants in ABCB11 (bile salt export pump) through LD. ABCB11 dysfunction may impair glucose homeostasis by altering bile acid-mediated FXR signaling (51).

The protective effect of the T allele of rs8192678 against GDM was not observed in populations from Scandinavia, Austria, and Italy (23–25). This indicates potential ethnic or population-specific differences in genetic factors affecting GDM risk. The varying minor allele frequencies of rs8192678 among populations likely explain the inconsistent associations with GDM, with the T allele frequencies being 44.25% in the East Asian population compared to 36.08% in the European population and 25.65% in the American population.^7^ Beyond the difference in T allele frequencies between Asian and European populations, the protective effect of rs8192678 (Gly482Ser) in the Asian population might be influenced by co-inherited variants in mitochondrial biogenesis pathways, such as TFAM rs1937, which are more prevalent in the Asian population than in the European population.^8^ These variants may affect PGC-1α’s role in placental metabolism. Environmental factors, such as BPA or dietary elements, may also modulate this effect. Our study reveals a more than 2-fold higher median BPA level (4.9 ng/mL) compared to the European population (1.8 ng/mL) (52). Higher BPA exposure may unmask genetic protection in contexts of metabolic stress (53, 54). Dietary modifiers, such as soy isoflavones (high in Asian diets), can compete with BPA for ERβ binding, potentially altering the protective effect of the rs8192678-T allele (55). Another possibility involves linkage disequilibrium (LD). In LD-based indirect correlation analysis, if a disease-causing locus and genetic markers (polymorphic alleles) exhibit strong LD, they can be compared to those in healthy individuals to determine the relative risk of disease-causing loci in the affected population. If the LD between the SNP and the causal loci is weaker in the European population, it may result in a less detectable association (56). If the effect size is very low in the European population, it could only be identified by increasing the statistical power through a larger sample size.

Our study found that GCK rs2971672 (A > C) was related to GDM, with the CC genotype linked to a reduced risk of GDM. Although no prior research directly linked this variant to GDM, this variant has been previously associated with elevated blood glucose and lipid metabolism (57–59). This intron variant has a minor C allele frequency that varies across populations, ranging from 41.15 to 61.88%.^9^ GCK encodes a hexokinase family protein essential for glucose-stimulated insulin secretion and glycogen synthesis (60). It employs multiple promoters and alternative splicing during its transcription process, leading to distinct isoforms of its coding protein. These isoforms exhibit tissue-specific expression in the pancreas and liver, allowing for precise regulation of glucose metabolism and insulin secretion in response to physiological needs. Although the exact functional implications of the rs2971672 variant are still unclear, it may affect gene transcription and enzyme activity, influencing metabolic processes (61). Although the interaction between GCK rs2971672 and the GPI missense variant rs8191371 T > C on GDM was not significant with an FDR greater than 0.1, this interaction may still be considered exploratory due to our limited power to detect interactions at FDR thresholds with the current sample size. As far as we know, the encoded product of GPI functions as a glycolytic enzyme (glucose-6-phosphate isomerase) that interconverts glucose-6-phosphate and fructose-6-phosphate. Further researches are needed to elucidate these relationships and their potential impact on GDM risk. GCK catalyzes the initial step of glycolysis, while GPI (glucose-6-phosphate isomerase) catalyzes the second step (17, 62). Altered GPI activity may lead to the accumulation of G6P (63), inhibiting hexokinase and disrupting insulin secretion (64). However, the function of rs2971672 remains unknown, as does that of rs8191371. Therefore, future studies should test PGC-1α binding to the G6PC2 promoter in hepatocyte models and measure glycolytic flux in β-cells co-expressing GCK/GPI variants. This corresponding text has been added to the discussion section.

In addition to genetic factors, environmental health factors significantly influence the occurrence and development of GDM. Exposure to endocrine disruptors has been linked to metabolic diseases, such as diabetes, and can disrupt glucose homeostasis. These substances can interfere with the synthesis, activity, and elimination of natural hormones that regulate glucose metabolism. By altering hormonal balance, endocrine disruptors may impact insulin sensitivity and glucose regulation, contributing to the development of metabolic disorders (65). Of concern, it was linked to altered metabolic pathways and an increased risk of GDM. These disruptors can impact the immunological and metabolic status of women during pregnancy by inducing cellular and molecular changes in maternal biological fluids and at the maternal–fetal interface (66). In this study, we examined urinary BPA concentrations in early pregnancy among 155 cases and controls to evaluate their association with GDM, considering potential genetic effects. Our findings revealed that the odds of GDM more than doubled with each unit increase in the natural square root-transformed urine BPA concentration, even after adjusting for maternal genetic effect, triglycerides, TSH, parity, gravidity, and age. This association remained consistent across different genetic models based on the PPARGC1A rs8192678 variant. Differently, previous studies have produced inconsistent results regarding the link between first-trimester BPA levels—whether in urine or serum—and GDM. Some cohort studies found no association (67–69), while a nested case–control study identified a positive correlation between first-trimester BPA and GDM risk among non-Asian/Pacific Islanders (70). Maternal early-pregnancy BPA exposure has been associated with glucose level, potentially exhibiting a non-linear relationship, although findings have been inconsistent (68, 69, 71–73). Previous research suggests that BPA may act through several pathways, such as the receptor pathways, disruption of the neuroendocrine system, modulation of immune and inflammatory responses, and epigenetic mechanisms (74). Low doses of BPA intake during gestation and early development have been shown to cause islet insulin hypersecretion in rat offspring for up to 1 year after exposure (75). Differentiated mature adipocytes exposed to BPA exhibited insulin resistance, with approximately a 25% reduction in insulin-stimulated glucose uptake (76). Ariemma et al. reported a 3.5-fold increase in the expression of peroxisome proliferator-activated receptor gamma (PPARγ) and a hyperregulation of inflammatory factors after 3 weeks of BPA exposure in 3 T3-L1 adipocytes, noting that BPA led to lipid accumulation and impaired insulin function, significantly reducing insulin-stimulated glucose utilization (77). LaRocca et al. found a correlation between placental miR-142-3p levels and first-trimester urinary phenols (78), while women later diagnosed with GDM exhibited higher first-trimester serum levels of miR-142-3p (79).

The subsequent marginal effect analysis revealed that the rs8192678-T allele’s protective effect exhibited a quasi-U-shaped distribution at varying BPA levels by primary marginal analysis and bootstrap validation (Supplementary Table S16). This association may be due to BPA’s non-monotonic dose–response effects (80). BPA exposure may influence hepatic ERα/ERβ homeostasis. While at a lower dose it promotes Akt phosphorylation, BPA at the higher dose attenuates ERK1/2 phosphorylation, suggesting potential alteration in insulin sensitivity (81). In this context, the rs8192678 (Gly482Ser) may change PGC-1α protein’s conformation and stability, synergistically altering placental mitochondrial function and modifying oxidative stress. In large cohorts, U-shaped BPA–T2DM/children’s blood pressure associations have been reported, which supports our finding (82).

Serum TG and TSH levels have been linked to an elevated risk of GDM (9, 10), while our study found consistent results for TG but inconsistent observations regarding TSH. Meanwhile, in the marginal effect analysis, the disparity in GDM probability between the TT genotype and the CC genotype of PPARGC1A rs8192678 decreased as serum TSH levels increased. As far as we know, adipose tissue TSH played a role in the maintenance of adipocyte mitochondrial function and regulating energy balance and adiposity by inhibiting the browning of white fat (83, 84). Both visceral and subcutaneous adipose tissue TSHB gene expression was positively correlated with the expression of mitochondrial function, such as PPARGC1A (84).

The protective effect of the PPARGC1A rs8192678-T allele decreases with higher TSH levels (Primary: dy/dx = −0.119 (−0.258 ~ 0.018), *p* = 0.09; validation: dy/dx = −0.119 (−0.277–0.037), *p* = 0.133) (Supplementary Table S16). This suggests that TSH-mediated thyroid hormone suppression counteracts genetic benefits. Elevated TSH can lower circulating free thyroxine (FT4) through the hypothalamic–pituitary–thyroid axis and downregulate thyroid receptor β-dependent PPARGC1A expression, potentially affecting tissue-specific mitochondrial function (85). The decreased FT4 would change thermal efficiency during energy conversion by regulating the expression of uncoupling proteins (UCPs) to improve (86, 87). Nevertheless, further external validation and the establishment of precise cut-offs for BPA and TSH levels to modulate the protective effect are necessary, ideally through studies with larger cohorts.

The primary strength of our study is revealing how the genetic effect of PPARGC1A rs8192678 on GDM slightly changes with urinary BPA and serum TSH level in a nested case–control design, which is based on a prospective cohort of nearly 3,000 pregnant women and highly efficient for avoiding reverse causation. Additionally, we accessed each participant’s OGTT data through the medical records system, with GDM diagnoses made by doctors according to IADPSG criteria. Another key strength is our use of both WES and candidate gene strategies to screen for potential SNPs, allowing us to visualize the molecular structures of the original and mutant amino acids of PPARGC1A and predict their functions. We also assessed the protein’s phase separation capability, finding evidence suggesting it may possess this ability, though further validation is needed.

While this study provides novel insights into gene–environment interactions in GDM pathogenesis, several limitations warrant acknowledgment. First, although we expanded adjustment to include pre-pregnancy BMI, gravidity, and parity—strengthening causal inference—residual confounding by unmeasured factors (e.g., dietary patterns and socioeconomic status) remains possible (52, 88, 89). Second, thyroid hormone interpretation is constrained by unavailable data on iodine supplementation or medications. However, all participants resided in iodine-sufficient regions (Shanghai) with universal salt iodization, minimizing population-level confounding. Third, exploratory and subgroup analyses were limited by sample size in genotype-exposure strata. We addressed this through rigorous bootstrap validation, confirming robust primary associations: rs8192678 TT vs. CC aOR = 0.417 (0.220–0.790), rs8192678 CT + TT vs. CC aOR = 0.593 (0.376–0.935), rs8192678 TT vs. CC + CT aOR = 0.524 (0.198–0.919) for exploratory analysis; BPA aOR = 2.295 (95% CI: 1.361–3.867) for subgroup analysis (Supplementary Table S17). Fourth, focusing solely on BPA overlooks mixtures of endocrine disruptors. Reassuringly, coexposure to BPA alternatives (BPS/BPF) is low in Chinese pregnant women (<10% detection; Spearman’s *r* = 0.10–0.17 vs. BPA), reducing confounding potential (89). Finally, the proposed RNF34-PGC-1α mechanism for rs8192678’s protective effect requires experimental validation. Future studies should construct the Gly482Ser mutant and validate changes in binding with RNF34 using co-immunoprecipitation (40).

## 5 Conclusion

In summary, our nested case–control study highlights the potential roles of genetic variants in GDM and identifies associations between GDM and the missense variant of PPARGC1A rs8192678, as well as the variant of GCK rs2971672. Sub-population analysis indicates that PPARGC1A rs8192678, BPA concentration, and serum TG and TSH levels are correlated with GDM. Marginal effect analysis further indicates the protective effect of PPARGC1A rs8192678 on GDM mildly varied with urinary BPA and serum TSH levels, even after controlling for potential confounders. However, further external validation and *in vitro* experiments are needed to confirm our findings.

## Data Availability

The datasets presented in this study can be found in online repositories. The names of the repository/repositories and accession number(s) can be found at: https://www.biosino.org/node/analysis/detail/OEZ00020943, OEZ00020943.

## Footnotes

^1^ http://sift.jcvi.org/

^2^ http://genetics.bwh.harvard.edu/pph2/

^3^ http://www.mutationtaster.org/

^4^ http://provean.jcvi.org/index.php

^5^ cadd.gs.washington.edu

^6^ http://metascape.org

^7^ http://www.mulinlab.org/vportal/apir.html?q=rs8192678&g=hg19

^8^ http://www.mulinlab.org/vportal/apir.html?q=rs1937&g=hg19

^9^ http://www.mulinlab.org/vportal/apir.html?q=rs2971672&g=hg19

## Glossary

GDM: Gestational diabetes mellitus
TG: Triglyceride
TSH: Thyroid-stimulating hormone
BPA: Bisphenol A
EDCs: Endocrine-disrupting chemicals
SNP: Single-nucleotide polymorphisms
T2D: Type 2 diabetes
PPARGC1A/PGC-1α: Peroxisome proliferator-activated receptor-γcoactivator-1α
WES: Whole exome sequencing
FBG: Fasting blood glucose
OGTT: oral glucose tolerance test
SNV: Single nucleotide variant
PS: Phase separation
SD: Standard deviation
MCODE: Molecular Complex Detection
OR: Odds ratio
CI: Confidence interval
SaPS: Self-assembling phase-separating predictor
PdPS: Ppartner-dependent phase-separating predictor
catGRANULE: Granule-forming propensity: predictor
PLAAC: Prion-like domain predictor
PScore: π-contact predictor
ESpritz: IDR predictor
SEG: low-complexity region predictor
Hydropathy: hydropathy prediction from CIDER
DeepCoil: Coiled-coil domain predictor
DeepPhase: Immunofluorescence image-based droplet-forming propensity predictor
MODY: Maturity-onset diabetes of the young
RNF34: Ring Finger Protein 34
PPARγ: Peroxisome proliferator-activated receptor gamma.
AME: Average marginal effect

## References

[1] Wang H Li N Chivese T Werfalli M Sun H Yuen L . IDF diabetes atlas: estimation of global and regional gestational diabetes mellitus prevalence for 2021 by International Association of Diabetes in pregnancy study group’s criteria. Diabetes Res Clin Pract. (2022) 183:109050. doi: 10.1016/j.diabres.2021.109050, PMID: 34883186

[2] Vounzoulaki E Khunti K Abner SC Tan BK Davies MJ Gillies CL. Progression to type 2 diabetes in women with a known history of gestational diabetes: systematic review and meta-analysis. BMJ. (2020) 369:m1361. doi: 10.1136/bmj.m1361, PMID: 32404325 PMC7218708

[3] Ye W Luo C Huang J Li C Liu Z Liu F. Gestational diabetes mellitus and adverse pregnancy outcomes: systematic review and meta-analysis. BMJ. (2022) 377:e067946. doi: 10.1136/bmj-2021-067946, PMID: 35613728 PMC9131781

[4] Lu J Zhang S Li W Leng J Wang L Liu H . Maternal gestational diabetes is associated with offspring’s hypertension. Am J Hypertens. (2019) 32:335–42. doi: 10.1093/ajh/hpz005, PMID: 30624576 PMC6420681

[5] McKenzie-Sampson S Paradis G Healy-Profitos J St-Pierre F Auger N. Gestational diabetes and risk of cardiovascular disease up to 25 years after pregnancy: a retrospective cohort study. Acta Diabetol. (2018) 55:315–22. doi: 10.1007/s00592-017-1099-2, PMID: 29327149

[6] Ijas H Koivunen S Raudaskoski T Kajantie E Gissler M Vaarasmaki M. Independent and concomitant associations of gestational diabetes and maternal obesity to perinatal outcome: a register-based study. PLoS One. (2019) 14:e0221549. doi: 10.1371/journal.pone.0221549, PMID: 31465425 PMC6715199

[7] Gunderson EP Sun B Catov JM Carnethon M Lewis CE Allen NB . Gestational diabetes history and glucose tolerance after pregnancy associated with coronary artery calcium in women during midlife: the CARDIA study. Circulation. (2021) 143:974–87. doi: 10.1161/CIRCULATIONAHA.120.047320, PMID: 33517667 PMC7940578

[8] Pazhohan A Rezaee Moradali M Pazhohan N. Association of first-trimester maternal lipid profiles and triglyceride-glucose index with the risk of gestational diabetes mellitus and large for gestational age newborn. J Matern Fetal Neonatal Med. (2019) 32:1167–75. doi: 10.1080/14767058.2017.1402876, PMID: 29157043

[9] Ryckman KK Spracklen CN Smith CJ Robinson JG Saftlas AF. Maternal lipid levels during pregnancy and gestational diabetes: a systematic review and meta-analysis. BJOG. (2015) 122:643–51. doi: 10.1111/1471-0528.13261, PMID: 25612005

[10] Huang K Su S Wang X Hu M Zhao R Gao S . Association between maternal thyroid function in early pregnancy and gestational diabetes: a prospective cohort study. J Clin Endocrinol Metab. (2024) 109:e780–7. doi: 10.1210/clinem/dgad518, PMID: 37647889 PMC10795920

[11] Eberle C Stichling S. Environmental health influences in pregnancy and risk of gestational diabetes mellitus: a systematic review. BMC Public Health. (2022) 22:1572. doi: 10.1186/s12889-022-13965-5, PMID: 35982427 PMC9389831

[12] Diabetes Genetics Initiative of Broad Institute of Harvard and MIT, Lund University, and Novartis Institutes of BioMedical Research Saxena R Voight BF Lyssenko V Burtt NP de Bakker PI . Genome-wide association analysis identifies loci for type 2 diabetes and triglyceride levels. Science. (2007) 316:1331–6. doi: 10.1126/science.1142358, PMID: 17463246

[13] Grarup N Rose CS Andersson EA Andersen G Nielsen AL Albrechtsen A . Studies of association of variants near the HHEX, CDKN2A/B, and IGF2BP2 genes with type 2 diabetes and impaired insulin release in 10,705 Danish subjects: validation and extension of genome-wide association studies. Diabetes. (2007) 56:3105–11. doi: 10.2337/db07-0856 17827400

[14] Palmer CN Maglio C Pirazzi C Burza MA Adiels M Burch L . Paradoxical lower serum triglyceride levels and higher type 2 diabetes mellitus susceptibility in obese individuals with the PNPLA3 148M variant. PLoS One. (2012) 7:e39362. doi: 10.1371/journal.pone.0039362, PMID: 22724004 PMC3377675

[15] Mahajan A Wessel J Willems SM Zhao W Robertson NR Chu AY . Refining the accuracy of validated target identification through coding variant fine-mapping in type 2 diabetes. Nat Genet. (2018) 50:559–71. doi: 10.1038/s41588-018-0084-1, PMID: 29632382 PMC5898373

[16] Zhang Y Li S Nie H Wang X Li X Wen J . The rs17782313 polymorphism near MC4R gene confers a high risk of obesity and hyperglycemia, while PGC1α rs8192678 polymorphism is weakly correlated with glucometabolic disorder: a systematic review and meta-analysis. Front Endocrinol (Lausanne). (2023) 14:1210455. doi: 10.3389/fendo.2023.1210455, PMID: 37621650 PMC10445758

[17] Abu-Aqel Y Alnesf A Aigha II Islam Z Kolatkar PR Teo A . Glucokinase (GCK) in diabetes: from molecular mechanisms to disease pathogenesis. Cell Mol Biol Lett. (2024) 29:120. doi: 10.1186/s11658-024-00640-3, PMID: 39245718 PMC11382428

[18] Ek J Andersen G Urhammer SA Gaede PH Drivsholm T Borch-Johnsen K . Mutation analysis of peroxisome proliferator-activated receptor-gamma coactivator-1 (PGC-1) and relationships of identified amino acid polymorphisms to type II diabetes mellitus. Diabetologia. (2001) 44:2220–6. doi: 10.1007/s001250100032, PMID: 11793024

[19] Hara K Tobe K Okada T Kadowaki H Akanuma Y Ito C . A genetic variation in the PGC-1 gene could confer insulin resistance and susceptibility to type II diabetes. Diabetologia. (2002) 45:740–3. doi: 10.1007/s00125-002-0803-z, PMID: 12107756

[20] Muller YL Bogardus C Pedersen O Baier L. A Gly482Ser missense mutation in the peroxisome proliferator-activated receptor gamma coactivator-1 is associated with altered lipid oxidation and early insulin secretion in Pima Indians. Diabetes. (2003) 52:895–8. doi: 10.2337/diabetes.52.3.895, PMID: 12606537

[21] Yang Y Mo X Chen S Lu X Gu D. Association of peroxisome proliferator-activated receptor gamma coactivator 1 alpha (PPARGC1A) gene polymorphisms and type 2 diabetes mellitus: a meta-analysis. Diabetes Metab Res Rev. (2011) 27:177–84. doi: 10.1002/dmrr.1158, PMID: 21294239

[22] Galipeau M Jouvet N Courty E Vandenbeek R Khan NP Bouyakdan K . 299-OR: molecular and physiological consequences of the diabetes-related PGC1A Gly482Ser polymorphism. Diabetes. (2022) 71 (Supplement_1):299–OR. doi: 10.2337/db22-299-OR, PMID: 36409792

[23] Shaat N Lernmark A Karlsson E Ivarsson S Parikh H Berntorp K . A variant in the transcription factor 7-like 2 (TCF7L2) gene is associated with an increased risk of gestational diabetes mellitus. Diabetologia. (2007) 50:972–9. doi: 10.1007/s00125-007-0623-2, PMID: 17342473

[24] Leipold H Knoefler M Gruber C Huber A Haslinger P Worda C. Peroxisome proliferator-activated receptor gamma coactivator-1alpha gene variations are not associated with gestational diabetes mellitus. J Soc Gynecol Investig. (2006) 13:104–7. doi: 10.1016/j.jsgi.2005.12.004, PMID: 16443502

[25] Franzago M Fraticelli F Nicolucci A Celentano C Liberati M Stuppia L . Molecular analysis of a genetic variants panel related to nutrients and metabolism: association with susceptibility to gestational diabetes and cardiometabolic risk in affected women. J Diabetes Res. (2017) 2017:4612623. doi: 10.1155/2017/4612623, PMID: 28133617 PMC5241477

[26] Bi Y Wang W Xu M Wang T Lu J Xu Y . Diabetes genetic risk score modifies effect of bisphenol A exposure on deterioration in glucose metabolism. J Clin Endocrinol Metab. (2016) 101:143–50. doi: 10.1210/jc.2015-3039, PMID: 26523527

[27] Ahmad N Shah SA Abdul Gafor AH Abdul Murad NA Kamaruddin MA Abd Jalal N . Gene-environment interaction in chronic kidney disease among people with type 2 diabetes from the Malaysian cohort project: a case-control study. Diabet Med. (2020) 37:1890–901. doi: 10.1111/dme.14257, PMID: 32012348

[28] Lu D Feng C Wang D Lin Y Ip HS She J . Analysis of twenty phenolic compounds in human urine: hydrochloric acid hydrolysis, solid-phase extraction based on K2CO 3-treated silica, and gas chromatography tandem mass spectrometry. Anal Bioanal Chem. (2015) 407:4131–41. doi: 10.1007/s00216-015-8598-1, PMID: 25903021

[29] Zhao H Li J Ma X Huo W Xu S Cai Z. Simultaneous determination of bisphenols, benzophenones and parabens in human urine by using UHPLC-TQMS. Chin Chem Lett. (2018) 29:102–6. doi: 10.1016/j.cclet.2017.06.013

[30] Borghese MM Huang R S MP Gaudreau E Gagné S Ashley-Martin J . A descriptive analysis of first trimester urinary concentrations of 14 bisphenol analogues in the MIREC Canadian pregnancy cohor. Int J Hyg Environ Health. (2023) 253:114225. doi: 10.1016/j.ijheh.2023.114225, PMID: 37542835

[31] Barrett ES Sathyanarayana S Mbowe O Thurston SW Redmon JB RHN N . First-trimester urinary bisphenol A concentration in relation to anogenital distance, an androgen-sensitive measure of reproductive development, in infant girls. Environ Health Perspect. (2017) 125:077008. doi: 10.1289/EHP875, PMID: 28728138 PMC5744699

[32] Zhou Y Zhou B Pache L Chang M Khodabakhshi AH Tanaseichuk O . Metascape provides a biologist-oriented resource for the analysis of systems-level datasets. Nat Commun. (2019) 10:1523. doi: 10.1038/s41467-019-09234-6, PMID: 30944313 PMC6447622

[33] Shi YY He L. SHEsis, a powerful software platform for analyses of linkage disequilibrium, haplotype construction, and genetic association at polymorphism loci. Cell Res. (2005) 15:97–8. doi: 10.1038/sj.cr.7290272, PMID: 15740637

[34] Shen J Li Z Chen J Song Z Zhou Z Shi Y. SHEsisPlus, a toolset for genetic studies on polyploid species. Sci Rep. (2016) 6:24095. doi: 10.1038/srep24095, PMID: 27048905 PMC4822172

[35] Williams R. Using the margins command to estimate and interpret adjusted predictions and marginal effects. Stata J. (2012) 12:308–31. doi: 10.1177/1536867X1201200209

[36] Jaaskelainen T Klemetti MM. Genetic risk factors and gene-lifestyle interactions in gestational diabetes. Nutrients. (2022) 14:4799. doi: 10.3390/nu14224799, PMID: 36432486 PMC9694797

[37] Bhatta P Bermano G Williams HC Knott RM. Meta-analysis demonstrates Gly482Ser variant of PPARGC1A is associated with components of metabolic syndrome within Asian populations. Genomics. (2020) 112:1795–803. doi: 10.1016/j.ygeno.2019.10.011, PMID: 31678594

[38] Bhat A Koul A Rai E Sharma S Dhar MK Bamezai RN. PGC-1alpha Thr394Thr and Gly482Ser variants are significantly associated with T2DM in two north Indian populations: a replicate case-control study. Hum Genet. (2007) 121:609–14. doi: 10.1007/s00439-007-0352-0, PMID: 17390150

[39] Andrulionyte L Zacharova J Chiasson JL Laakso M S.-N.S. Group. Common polymorphisms of the PPAR-gamma2 (Pro12Ala) and PGC-1alpha (Gly482Ser) genes are associated with the conversion from impaired glucose tolerance to type 2 diabetes in the STOP-NIDDM trial. Diabetologia. (2004) 47:2176–84. doi: 10.1007/s00125-004-1577-2 15592662

[40] Huang M Claussnitzer M Saadat A Coral DE Kalamajski S Franks PW. Engineered allele substitution at PPARGC1A rs8192678 alters human white adipocyte differentiation, lipogenesis, and PGC-1alpha content and turnover. Diabetologia. (2023) 66:1289–305. doi: 10.1007/s00125-023-05915-6, PMID: 37171500 PMC10244287

[41] Eid S Sas KM Abcouwer SF Feldman EL Gardner TW Pennathur S . New insights into the mechanisms of diabetic complications: role of lipids and lipid metabolism. Diabetologia. (2019) 62:1539–49. doi: 10.1007/s00125-019-4959-1, PMID: 31346658 PMC6679814

[42] Taghvaei S Saremi L Babaniamansour S. Computational analysis of Gly482Ser single-nucleotide polymorphism in PPARGC1A gene associated with CAD, NAFLD, T2DM, obesity, hypertension, and metabolic diseases. PPAR Res. (2021) 5:5544233. doi: 10.1155/2021/5544233 PMC8360745 34394332

[43] Wei P Guo J Xue W Zhao Y Yang J Wang J. RNF34 modulates the mitochondrial biogenesis and exercise capacity in muscle and lipid metabolism through ubiquitination of PGC-1 in Drosophila. Acta Biochim Biophys Sin Shanghai. (2018) 50:1038–46. doi: 10.1093/abbs/gmy106, PMID: 30247505

[44] Wei P Pan D Mao C Wang YX. RNF34 is a cold-regulated E3 ubiquitin ligase for PGC-1alpha and modulates brown fat cell metabolism. Mol Cell Biol. (2012) 32:266–75. doi: 10.1128/MCB.05674-11, PMID: 22064484 PMC3255768

[45] Tan LS Lau HH Abdelalim EM Khoo CM O’Brien RM Tai ES . The role of glucose-6-phosphatase activity in glucose homeostasis and its potential for diabetes therapy. Trends Mol Med. (2024) 31:152–64. doi: 10.1016/j.molmed.2024.09.005 39426930

[46] Ebert DH Bischof LJ Streeper RS Chapman SC Svitek CA Goldman JK . Structure and promoter activity of an islet-specific glucose-6-phosphatase catalytic subunit-related gene. Diabetes. (1999) 48:543–51. doi: 10.2337/diabetes.48.3.543, PMID: 10078554

[47] Lee J Salazar Hernández MA Auen T Mucka P Lee J Ozcan U. PGC-1α functions as a co-suppressor of XBP1s to regulate glucose metabolism. Mol Metab. (2018) 7:119–31. doi: 10.1016/j.molmet.2017.10.010, PMID: 29129613 PMC5784318

[48] Matsumoto M Pocai A Rossetti L Depinho RA Accili D. Impaired regulation of hepatic glucose production in mice lacking the forkhead transcription factor Foxo1 in liver. Cell Metab. (2007) 6:208–16. doi: 10.1016/j.cmet.2007.08.006, PMID: 17767907

[49] Hu C Zhang R Wang C Ma X Wang C Fang Q . A genetic variant of G6PC2 is associated with type 2 diabetes and fasting plasma glucose level in the Chinese population. Diabetologia. (2009) 52:451–6. doi: 10.1007/s00125-008-1241-3, PMID: 19082990

[50] Takeuchi F Katsuya T Chakrewarthy S Yamamoto K Fujioka A Serizawa M . Common variants at the GCK, GCKR, G6PC2-ABCB11 and MTNR1B loci are associated with fasting glucose in two Asian populations. Diabetologia. (2010) 53:299–308. doi: 10.1007/s00125-009-1595-1, PMID: 19937311

[51] Yan Y Niu Z Sun C Li P Shen S Liu S . Hepatic thyroid hormone signalling modulates glucose homeostasis through the regulation of GLP-1 production via bile acid-mediated FXR antagonism. Nat Commun. (2022) 13:6408. doi: 10.1038/s41467-022-34258-w, PMID: 36302774 PMC9613917

[52] Covaci A Den Hond E Geens T Govarts E Koppen G Frederiksen H . Urinary BPA measurements in children and mothers from six European member states: overall results and determinants of exposure. Environ Res. (2015) 141:77–85. doi: 10.1016/j.envres.2014.08.008, PMID: 25440295

[53] Xiang Z Wang H Zhu K Liu R Zhao S Fan H . Phenol exposure, polygenic risk score, and dyslexia in Chinese children: gene-environment interaction. Environ Pollut. (2025) 379:126536. doi: 10.1016/j.envpol.2025.126536, PMID: 40425062

[54] Choi YJ Lee YA Hong YC Cho J Lee KS Shin CH . Effect of prenatal bisphenol A exposure on early childhood body mass index through epigenetic influence on the insulin-like growth factor 2 receptor (IGF2R) gene. Environ Int. (2020) 143:105929. doi: 10.1016/j.envint.2020.105929, PMID: 32645488

[55] Hwang KA Kang NH Yi BR Lee HR Park MA Choi KC. Genistein, a soy phytoestrogen, prevents the growth of BG-1 ovarian cancer cells induced by 17β-estradiol or bisphenol A via the inhibition of cell cycle progression. Int J Oncol. (2013) 42:733–40. doi: 10.3892/ijo.2012.1719, PMID: 23229410

[56] Lu Y Loos RJ. Obesity genomics: assessing the transferability of susceptibility loci across diverse populations. Genome Med. (2013) 5:55. doi: 10.1186/gm459, PMID: 23806069 PMC3706771

[57] Kanai M Akiyama M Takahashi A Matoba N Momozawa Y Ikeda M . Genetic analysis of quantitative traits in the Japanese population links cell types to complex human diseases. Nat Genet. (2018) 50:390–400. doi: 10.1038/s41588-018-0047-6, PMID: 29403010

[58] Klarin D Damrauer SM Cho K Sun YV Teslovich TM Honerlaw J . Genetics of blood lipids among ~300,000 multi-ethnic participants of the million veteran program. Nat Genet. (2018) 50:1514–23. doi: 10.1038/s41588-018-0222-9, PMID: 30275531 PMC6521726

[59] Hong MG Karlsson R Magnusson PK Lewis MR Isaacs W Zheng LS . A genome-wide assessment of variability in human serum metabolism. Hum Mutat. (2013) 34:515–24. doi: 10.1002/humu.22267, PMID: 23281178

[60] Matschinsky FM Magnuson MA Zelent D Jetton TL Doliba N Han Y . The network of glucokinase-expressing cells in glucose homeostasis and the potential of glucokinase activators for diabetes therapy. Diabetes. (2006) 55:1–12. doi: 10.2337/diabetes.55.01.06.db05-0926, PMID: 16380470

[61] Vaz-Drago R Custodio N Carmo-Fonseca M. Deep intronic mutations and human disease. Hum Genet. (2017) 136:1093–111. doi: 10.1007/s00439-017-1809-4, PMID: 28497172

[62] Wang Q Liu J Chen Z Zheng J Wang Y Dong J. Targeting metabolic reprogramming in hepatocellular carcinoma to overcome therapeutic resistance: a comprehensive review. Biomed Pharmacother. (2024) 170:116021. doi: 10.1016/j.biopha.2023.116021, PMID: 38128187

[63] Davogustto GE Salazar RL Vasquez HG Karlstaedt A Dillon WP Guthrie PH . Metabolic remodeling precedes mTORC1-mediated cardiac hypertrophy. J Mol Cell Cardiol. (2021) 158:115–27. doi: 10.1016/j.yjmcc.2021.05.016, PMID: 34081952 PMC8630806

[64] Jeukendrup AE. Regulation of fat metabolism in skeletal muscle. Ann N Y Acad Sci. (2002) 967:217–35. doi: 10.1111/j.1749-6632.2002.tb04278.x 12079850

[65] Moreno-Gomez-Toledano R Delgado-Marin M Cook-Calvete A Gonzalez-Cucharero C Alcharani N Jimenez-Guirado B . New environmental factors related to diabetes risk in humans: emerging bisphenols used in synthesis of plastics. World J Diabetes. (2023) 14:1301–13. doi: 10.4239/wjd.v14.i8.1301, PMID: 37664470 PMC10473949

[66] Merrill AK Sobolewski M Susiarjo M. Exposure to endocrine disrupting chemicals impacts immunological and metabolic status of women during pregnancy. Mol Cell Endocrinol. (2023) 577:112031. doi: 10.1016/j.mce.2023.112031, PMID: 37506868 PMC10592265

[67] Shapiro GD Dodds L Arbuckle TE Ashley-Martin J Fraser W Fisher M . Exposure to phthalates, bisphenol A and metals in pregnancy and the association with impaired glucose tolerance and gestational diabetes mellitus: the MIREC study. Environ Int. (2015) 83:63–71. doi: 10.1016/j.envint.2015.05.016, PMID: 26101084

[68] Zhang W Xia W Liu W Li X Hu J Zhang B . Exposure to bisphenol A substitutes and gestational diabetes mellitus: a prospective cohort study in China. Front Endocrinol (Lausanne). (2019) 10:262. doi: 10.3389/fendo.2019.00262, PMID: 31114544 PMC6503732

[69] Yang J Wang H Du H Xu L Liu S Yi J . Serum bisphenol A, glucose homeostasis, and gestational diabetes mellitus in Chinese pregnant women: a prospective study. Environ Sci Pollut Res Int. (2021) 28:12546–54. doi: 10.1007/s11356-020-11263-4, PMID: 33083951

[70] Zhu Y Hedderson MM Calafat AM Alexeeff SE Feng J Quesenberry CP . Urinary phenols in early to midpregnancy and risk of gestational diabetes mellitus: a longitudinal study in a multiracial cohort. Diabetes. (2022) 71:2539–51. doi: 10.2337/db22-0028, PMID: 36227336 PMC9750951

[71] Bellavia A Cantonwine DE Meeker JD Hauser R Seely EW McElrath TF . Pregnancy urinary bisphenol-a concentrations and glucose levels across BMI categories. Environ Int. (2018) 113:35–41. doi: 10.1016/j.envint.2018.01.012, PMID: 29421405 PMC6583793

[72] Chiu YH Minguez-Alarcon L Ford JB Keller M Seely EW Messerlian C . Trimester-specific urinary bisphenol A concentrations and blood glucose levels among pregnant women from a fertility clinic. J Clin Endocrinol Metab. (2017) 102:1350–7. doi: 10.1210/jc.2017-00022, PMID: 28323984 PMC5460734

[73] Fisher BG Frederiksen H Andersson AM Juul A Thankamony A Ong KK . Serum phthalate and triclosan levels have opposing associations with risk factors for gestational diabetes mellitus. Front Endocrinol (Lausanne). (2018) 9:99. doi: 10.3389/fendo.2018.00099, PMID: 29593656 PMC5859030

[74] Ma Y Liu H Wu J Yuan L Wang Y Du X . The adverse health effects of bisphenol A and related toxicity mechanisms. Environ Res. (2019) 176:108575. doi: 10.1016/j.envres.2019.108575, PMID: 31299621

[75] Manukyan L Dunder L Lind PM Bergsten P Lejonklou MH. Developmental exposure to a very low dose of bisphenol A induces persistent islet insulin hypersecretion in Fischer 344 rat offspring. Environ Res. (2019) 172:127–36. doi: 10.1016/j.envres.2019.02.009, PMID: 30782532

[76] De Filippis E Li T Rosen ED. Exposure of adipocytes to bisphenol-a in vitro interferes with insulin action without enhancing adipogenesis. PLoS One. (2018) 13:e0201122. doi: 10.1371/journal.pone.0201122, PMID: 30133442 PMC6104924

[77] Ariemma F D’Esposito V Liguoro D Oriente F Cabaro S Liotti A . Low-dose bisphenol-a impairs adipogenesis and generates dysfunctional 3T3-L1 adipocytes. PLoS One. (2016) 11:e0150762. doi: 10.1371/journal.pone.0150762, PMID: 26942597 PMC4778877

[78] LaRocca J Binder AM McElrath TF Michels KB. First-trimester urine concentrations of phthalate metabolites and phenols and placenta miRNA expression in a cohort of U.S. women. Environ Health Perspect. (2016) 124:380–7. doi: 10.1289/ehp.1408409, PMID: 26090578 PMC4786977

[79] Juchnicka I Kuzmicki M Niemira M Bielska A Sidorkiewicz I Zbucka-Kretowska M . miRNAs as predictive factors in early diagnosis of gestational diabetes mellitus. Front Endocrinol (Lausanne). (2022) 13:839344. doi: 10.3389/fendo.2022.839344, PMID: 35340328 PMC8948421

[80] Vandenberg LN Colborn T Hayes TB Heindel JJ Jacobs DR Jr Lee DH . Hormones and endocrine-disrupting chemicals: low-dose effects and nonmonotonic dose responses. Endocr Rev. (2012) 33:378–455. doi: 10.1210/er.2011-1050, PMID: 22419778 PMC3365860

[81] Mukherjee U Samanta A Biswas S Das S Ghosh S Mandal DK . Bisphenol A-induced oxidative stress, hepatotoxicity and altered estrogen receptor expression in *Labeo bata*: impact on metabolic homeostasis and inflammatory response. Ecotoxicol Environ Saf. (2020) 202:110944. doi: 10.1016/j.ecoenv.2020.110944, PMID: 32800225

[82] Duan Y Yao Y Wang B Han L Wang L Sun H . Association of urinary concentrations of bisphenols with type 2 diabetes mellitus: a case-control study. Environ Pollut. (2018) 243:1719–26. doi: 10.1016/j.envpol.2018.09.093, PMID: 30408859

[83] Comas F Lluch A Sabater M Latorre J Ortega F Ricart W . Adipose tissue TSH as a new modulator of human adipocyte mitochondrial function. Int J Obes (Lond). (2019) 43:1611–9. doi: 10.1038/s41366-018-0203-1, PMID: 30206337

[84] Zhang J Wu H Ma S Gao L Yu C Jing F . TSH promotes adiposity by inhibiting the browning of white fat. Adipocyte. (2020) 9:264–78. doi: 10.1080/21623945.2020.1783101, PMID: 32579056 PMC7469524

[85] Sinha RA Singh BK Yen PM. Direct effects of thyroid hormones on hepatic lipid metabolism. Nat Rev Endocrinol. (2018) 14:259–69. doi: 10.1038/nrendo.2018.10, PMID: 29472712 PMC6013028

[86] Mullur R Liu YY Brent GA. Thyroid hormone regulation of metabolism. Physiol Rev. (2014) 94:355–82. doi: 10.1152/physrev.00030.2013, PMID: 24692351 PMC4044302

[87] Sinha RA Yen PM. Metabolic messengers: thyroid hormones. Nat Metab. (2024) 6:639–50. doi: 10.1038/s42255-024-00986-0, PMID: 38671149 PMC7615975

[88] Chen WJ Robledo C Davis EM Goodman JR Xu C Hwang J . Assessing urinary phenol and paraben mixtures in pregnant women with and without gestational diabetes mellitus: a case-control study. Environ Res. (2022) 214:113897. doi: 10.1016/j.envres.2022.113897, PMID: 35839910 PMC9514543

[89] Wang Z Miao M Xu J Chen Y Liang H Yang L . Gestational exposure to bisphenol analogues and kisspeptin levels in pregnant women and their children: a pregnancy-birth cohort study. Sci Total Environ. (2022) 848:157720. doi: 10.1016/j.scitotenv.2022.157720 35914601

