# Supplementary material for: Genetic susceptibility to gestational diabetes and its mild modification by bisphenol A and thyroid-stimulating hormone: findings from a South Chinese pregnancy cohort
Source: Front Nutr. 2025 Dec 10;12:1652265. doi: 10.3389/fnut.2025.1652265 (PMC12729077; doi:10.3389/fnut.2025.1652265)
Supplement: Supplementary file 1 [file Image_1.PDF]

Figure S1

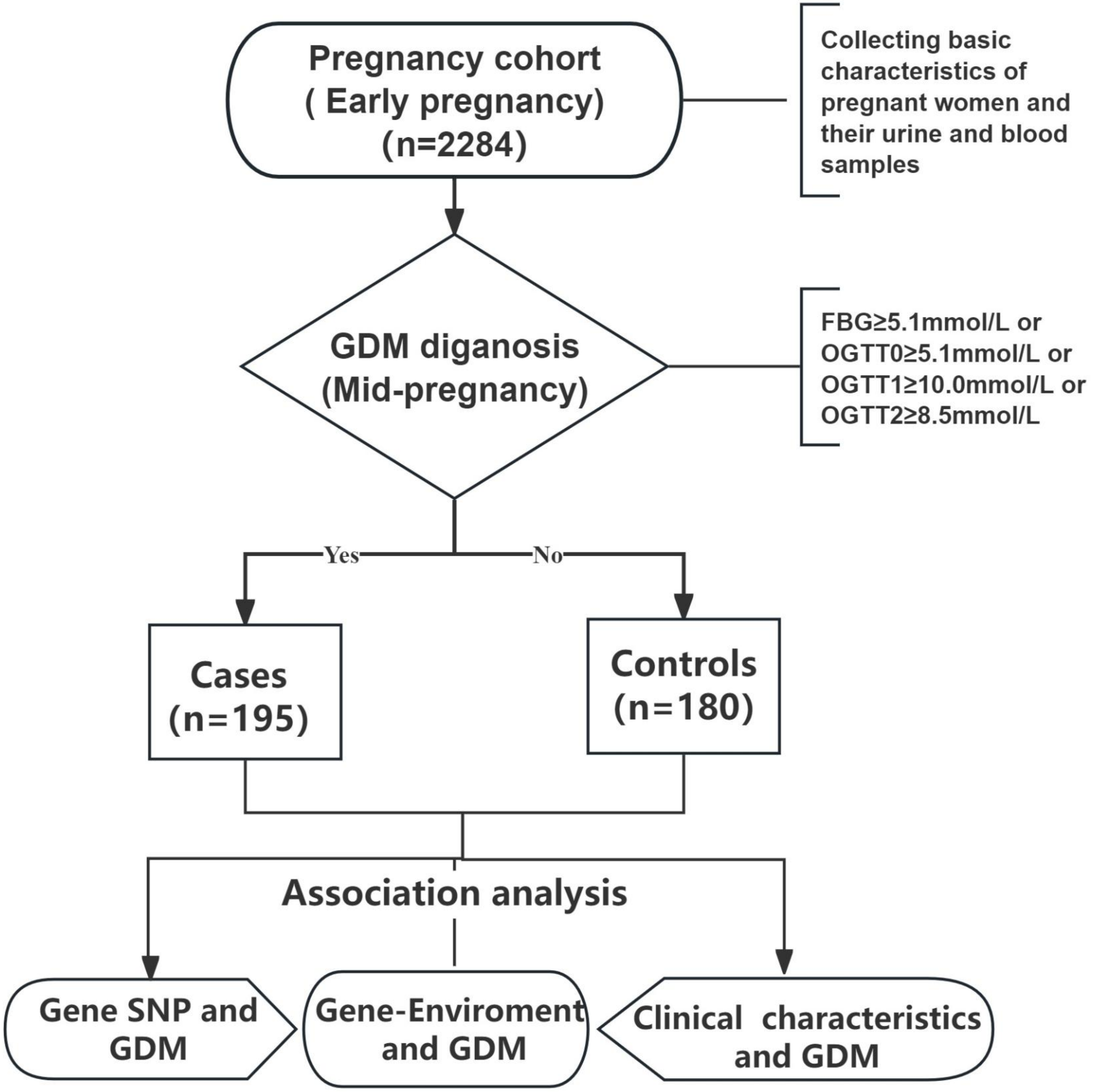

Pregnancy cohort
(Early pregnancy)
(n=2284)
Collecting basic characteristics of pregnant women and their urine and blood samples
GDM diganosis
(Mid-pregnancy)
FBG≥5.1mmol/L or
OGTT0≥5.1mmol/L or
OGTT1≥10.0mmol/L or
OGTT2≥8.5mmol/L
Yes
No
Cases
(n=195)
Controls
(n=180)
Association analysis
Gene SNP and GDM
Gene-Enviroment and GDM
Clinical characteristics and GDM

Figure S2

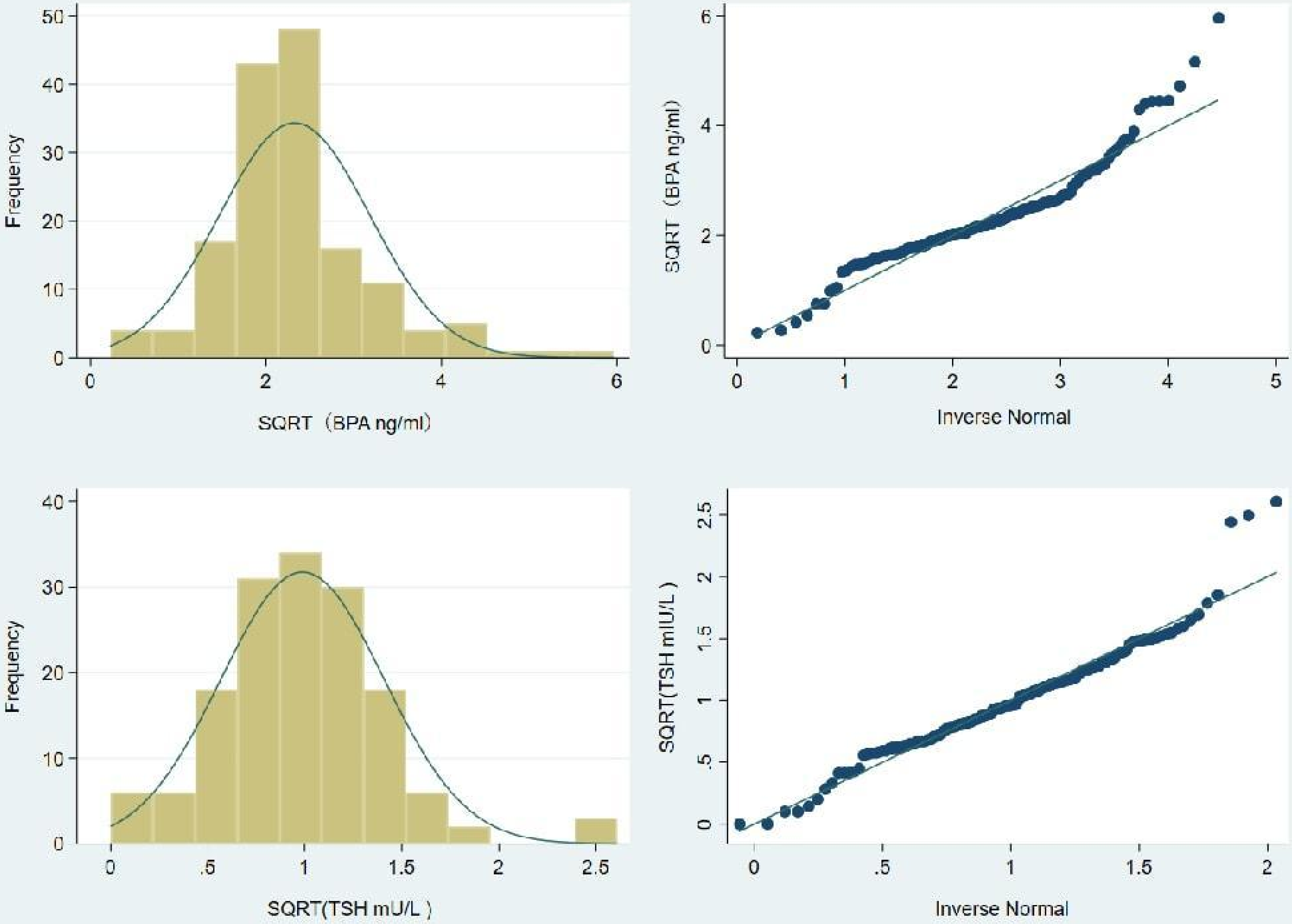
Frequency
SQRT（BPA ng/ml)
SQRT（BPA ng/ml)
Inverse Normal
Frequency
SQRT(TSH mU/L)
SQRT(TSH mIU/L)
Inverse Normal

Figure S3

(a)

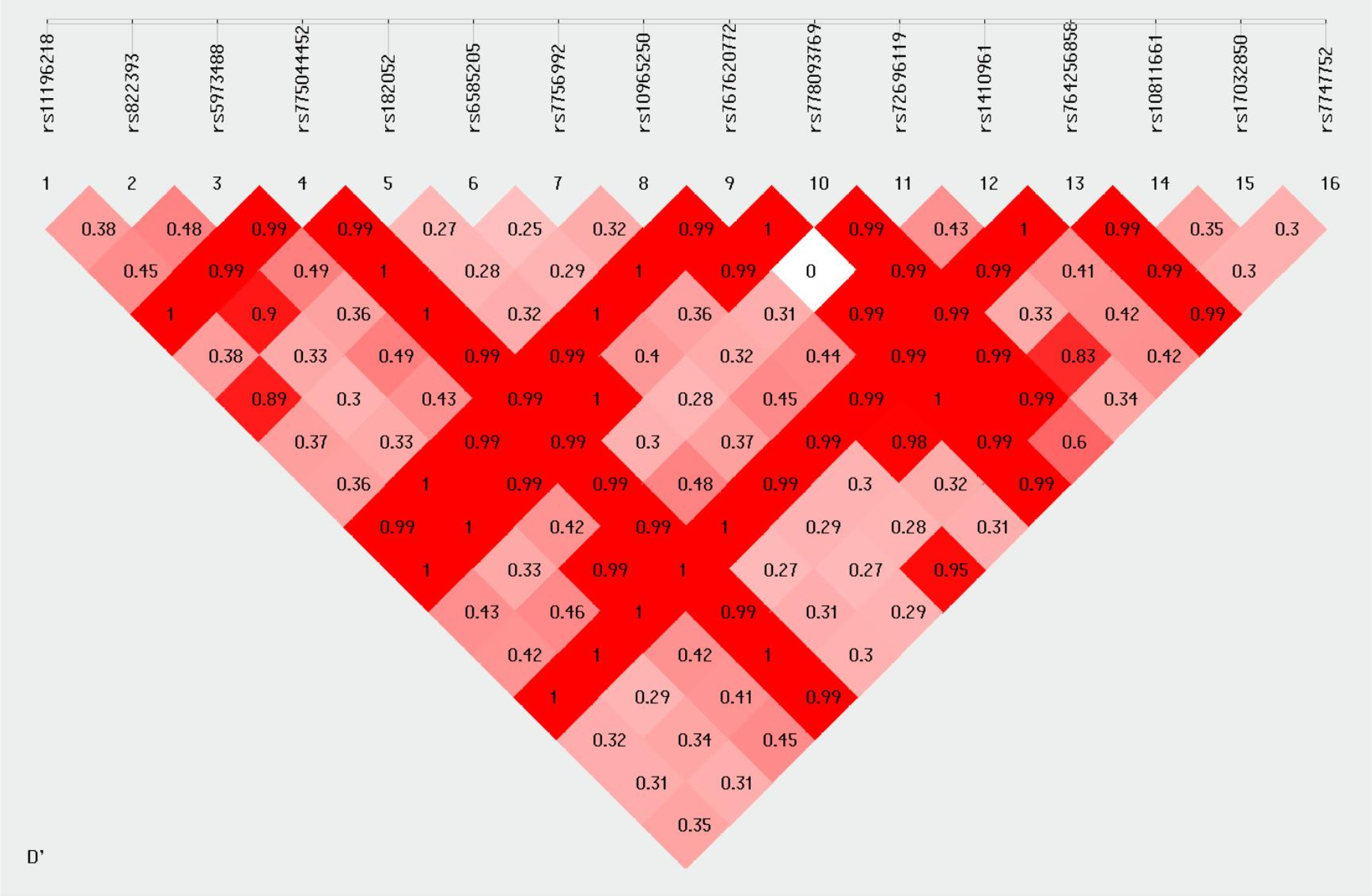

rs11196218
rs822393
rs5973488
rs775044452
rs182052
rs6585205
rs7756992
rs10965250
rs767620772
rs778093769
rs72696119
rs1410961
rs764256858
rs10811661
rs17032850
rs7747752
1
2
3
4
5
6
7
8
9
10
11
12
13
14
15
16
D'

(b)

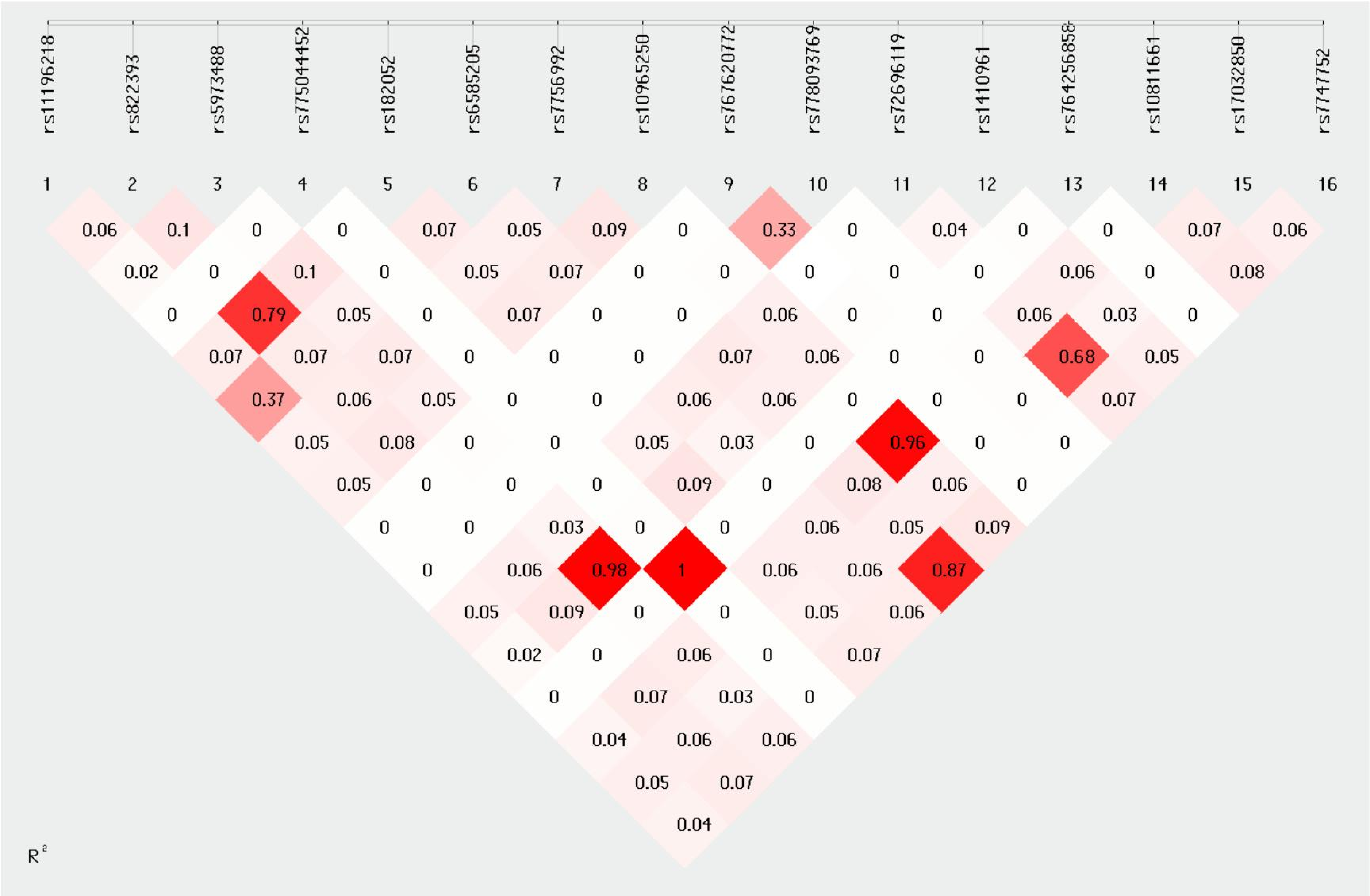

rs11196218
rs822393
rs5973488
rs775044452
rs182052
rs6585205
rs7756992
rs10965250
rs767620772
rs778093769
rs72696119
rs1410961
rs764256858
rs10811661
rs17032850
rs7747752
1
2
3
4
5
6
7
8
9
10
11
12
13
14
15
16
R²

Figure S4

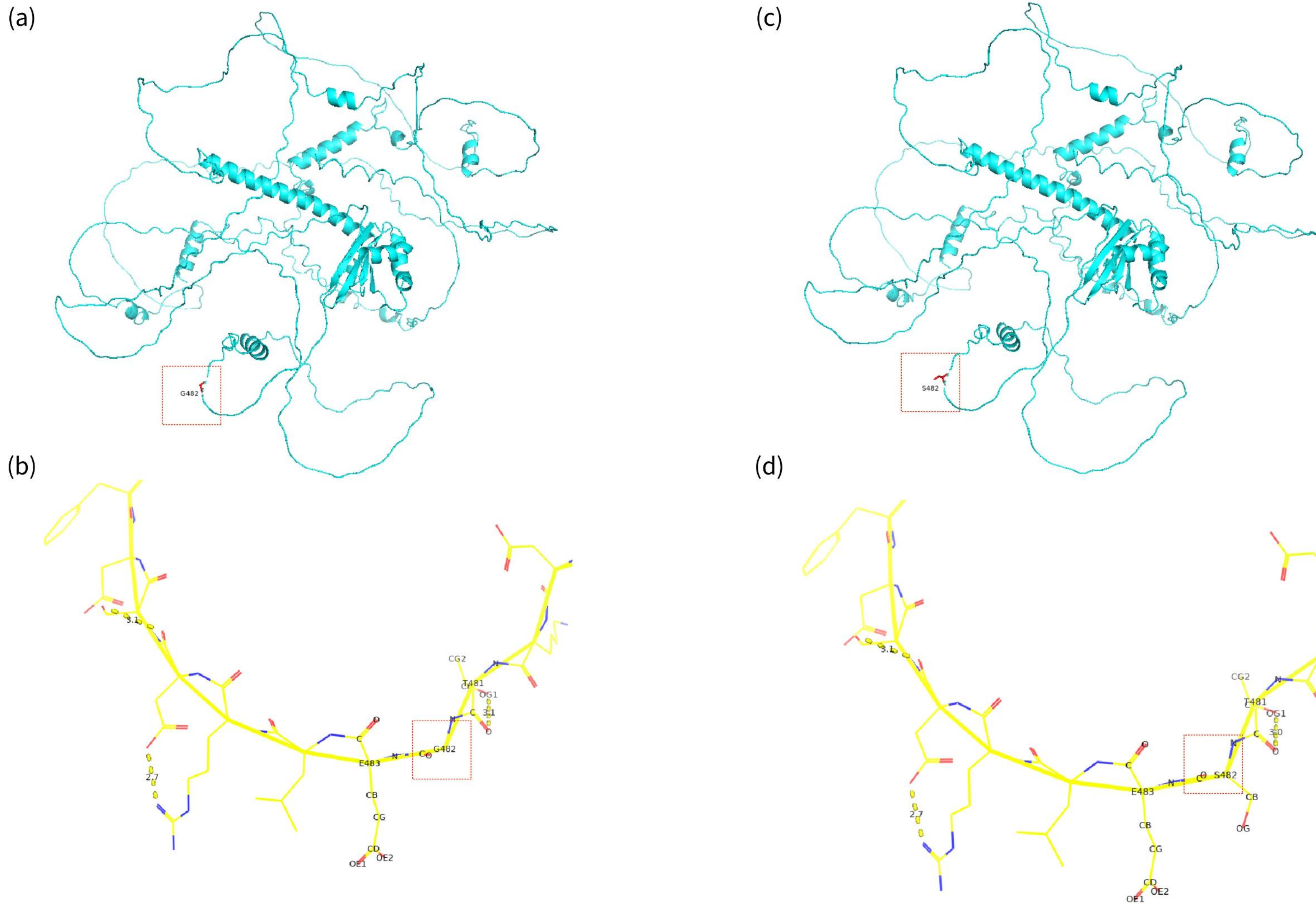
(a)
G482
(b)
3.1
2.7
CG2
T481
OG1
3.1
N
C
O
G482
E483
CB
CG
CD
OE1
OE2
(c)
S482
(d)
3.1
2.7
CG2
T481
OG1
3.0
S482
E483
CB
OG
CG
CD
OE1
OE2

Figure S5

(a)

Protein Information					
Uniprot Entry	Q9UBK2	Entry name	PRGC1_HUMAN	Gene name	PPARGC1A LEM6 PGC1 PGC1A PPARGC1
Length	798	Status	reviewed	Organism	Homo sapiens (Human)

SePred Scores	Score (8-feature)	Rank (8-feature)	Score (10-feature)	Rank (10-feature)
PS-Self score (Proteins that can self-assemble to form condensates)	0.364	0.757	0.435	0.824
PS-Part score (Proteins whose phase separation behaviors are regulated by protein or nucleic acid partner components)	0.840	0.978	0.830	0.937

* The 8-feature model incorporates Hydropathy, FCR, IDR, LCR, PScore, PLAAC, catGRANULE, and DeepCoil. The 10-feature model incorporates the 8 features described above plus Phos frequency and DeepPhase. This model is only available for human proteins.

Related Scores and ranks in proteome

	catGRANULE	PLAAC	Pscore	ESpritz (DisProt)	Hydropathy	SEG	Charged residue	Phos frequency	DeepPhase	DeepCoil
Score	0.733	-0.083	3.460	0.035	0.377	0.199	0.294	0.026	0.299	0.000
Rank	0.783	0.851	0.922	0.584	0.027	0.867	0.882	0.752	0.085	/

* The ranking of feature values was evaluated in the proteome of the corresponding species. 1-ranking was shown for each feature value (The highest Rank score is 1 and the lowest Rank score is 0).

Radar map for the rank of each feature

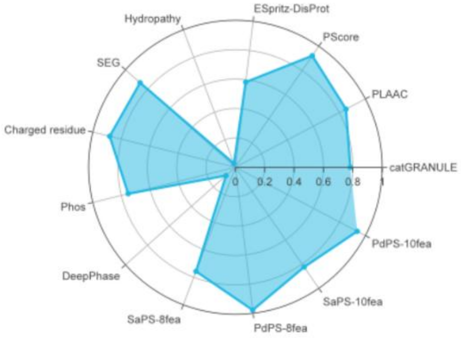

Immunofluorescence image
https://doi.org/10.1093/bib/bbaa187

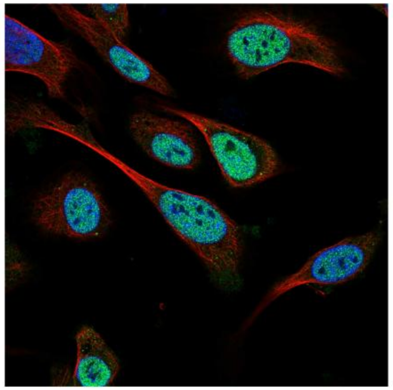

(b)

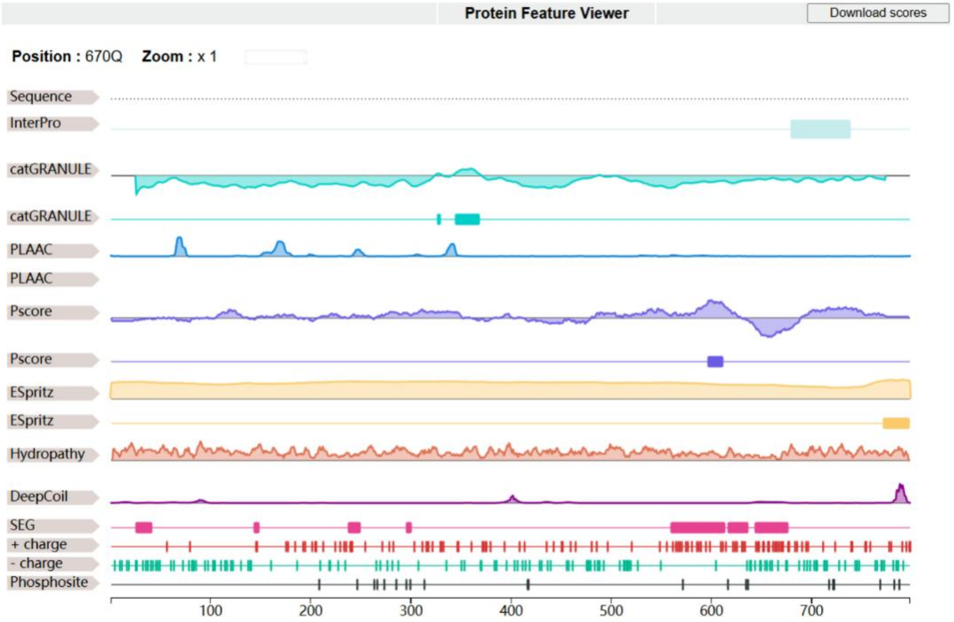

(c)

Protein Sequence Viewer

MAWDMCNQDS ESVWSDIECA ALVGEDQPLC PDLPELDLSE LDVNDLDTDS FLGGLKWCSD QSEIISNQYN
NEPSNIFEKI DEENEANLLA VLTETLDSLP VDEDGLPSFD ALTDGDVTTD NEASPSSMPD GTPPPQEAEE
PSLLKKLLLA PANTQLSYNE CSGLSTQNHA NHNHRIRTNP AIVKTENSWS NKAKSICQQQ KPQRRPCSEL
LKYLTTNDDP PHTKPTENRN SSRDKCTSKK KSHTQSQSQH LQAKPTTLSL PLTPESPNDP KGSPFENKTI
ERTLSVELSG TAGLTPPTTP PHKANQDNPF RASPKLKSSC KTVVPPPSKK PRYSESSGTQ GNNSTKKGPE
QSELYAQLSK SSVLTGGHEE RKTKRPSLRL FGDHDYCQSI NSKTEILINI SQELQDSRQL ENKDVSSDWQ
GQICSSTDSD QCYLRETLEA SKQVSPCSTR KQLQDQEIRA ELNKHFGHPS QAVFDDEADK TGELRDSDFS
NEQFSKLPMF INSGLAMDGL FDDSEDESDK LSYPWDGTQS YSLFNVSPSC SSFNSPCRDS VSPPKSLFSQ
RPQRMRSRSR SFSRHRSCSR SPYSRSRSRS PGSRSSSRSC YYYESSHYRH RTHRNSPLYV RSRSRSPYSR
RPRYDSYEEY QHERLKREEY RREYEKRESE RAKQRERQRQ KAIEERRVIY VGKIRPDTTR TELRDRFEVF
GEIEECTVNL RDDGDSYGFI TYRYTCDAFA ALENGYTLRR SNETDFELYF CGRKQFFKSN YADLDSNSDD
FDPASTKSKY DSLDFDSLLK EAQRSLRR

alphatic	GAVLMI
aromatic	FYW
postivecharge	KRH
negativecharge	DE
uncharge	STCPNQ

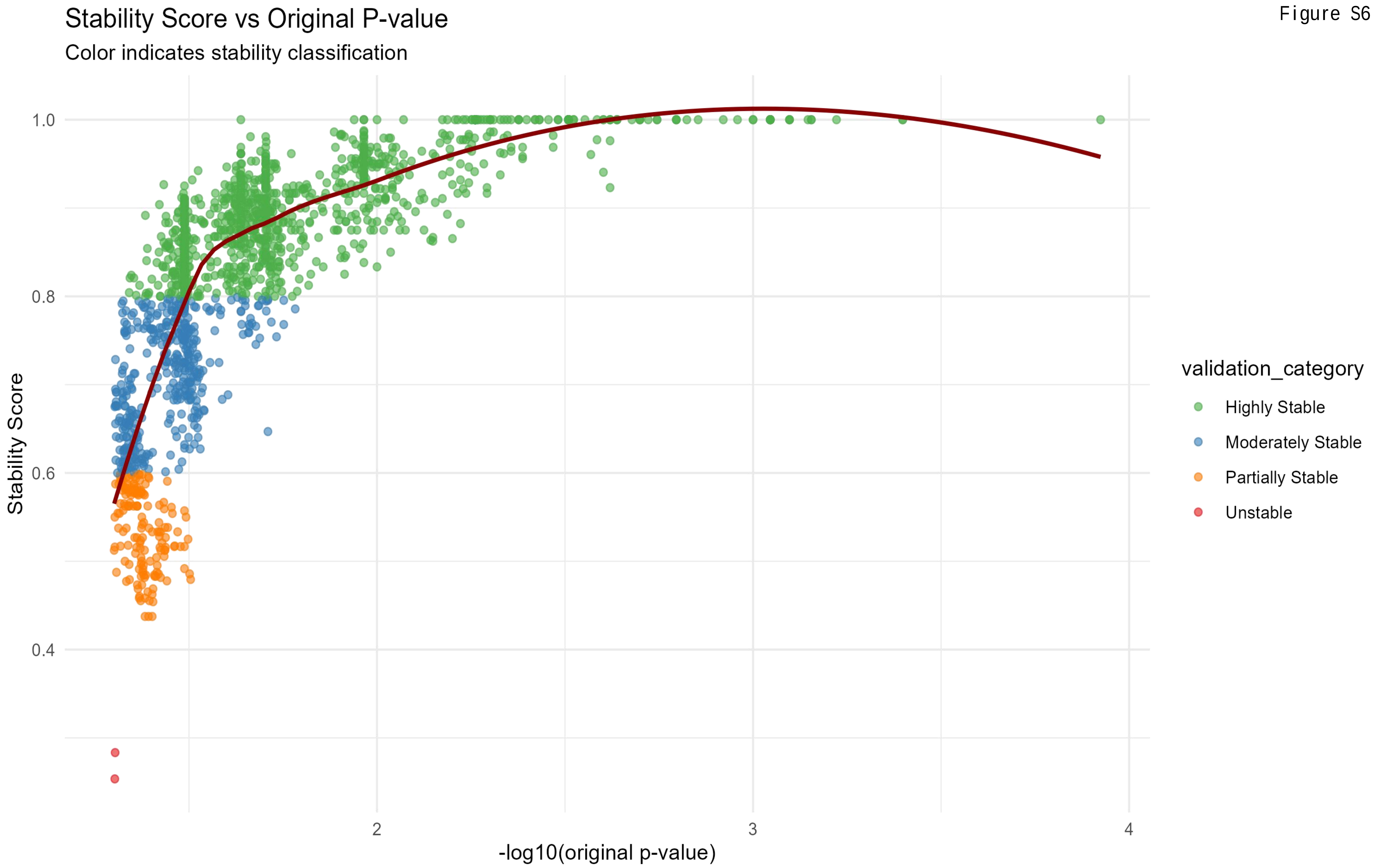
Stability Score vs Original P-value
Color indicates stability classification
Stability Score
1.0
0.8
0.6
0.4
2
3
4
-log10(original p-value)
validation_category
Highly Stable
Moderately Stable
Partially Stable
Unstable

Figure S1 .Flowchart of the nested case control study.

Figure S2. Histogram and Q-Q plot for BPA and TSH concentrations after square transformation.

Figure S3. D’and R2 for linkage disequilibrium analysis of 16 variants among the 63 candidate SNVs: The two variants with both D’ > 0.8 and R2 >0.33 were defined as having a strong relationship in dark red.

Figure S4.Schematic structure of the original and mutant amino acids of PPARGC1A p.Gly482Ser. (a) Protein structure for the original amino acid (G482); (b) Measurement of the hydrogen bond near G482; (c)Protein structure for the mutant amino acid (S482); (d) Measurement of the hydrogen bond near S482. PPARGC1A p.Gly482Ser were within the red marked region.

Figure S5. The predicted results provided by PhaSePred for PPARGC1A. (a) Schematic view of the information includes the protein information and the PhaSePred and related scores, (b) the protein feature viewer (blue dotted box), and (c) the protein sequence viewer.

Figure S6. Graph depicting stable scores versus original p-values for the 1770 significant variants identified via whole-exome sequencing.
