# Supplementary material for: Genetic susceptibility to gestational diabetes and its mild modification by bisphenol A and thyroid-stimulating hormone: findings from a South Chinese pregnancy cohort
Source: Front Nutr. 2025 Dec 10;12:1652265. doi: 10.3389/fnut.2025.1652265 (PMC12729077; doi:10.3389/fnut.2025.1652265)
Supplement: Supplementary file 2 [file Table_1.DOCX]

**Supplementary tables**

Table S1. Paired primers of 63candidate SNVs for sequenom massarray genotyping from whole exome sequencing results and literature

| TERM | SNP_ID | 2nd-PCRP | 1st-PCRP | AMP_LEN | UEP_DIR | UEP_MASS | UEP_SEQ | EXT1_CALL | EXT1_MASS | EXT1_SEQ | EXT2_CALL | EXT2_MASS | EXT2_SEQ |
| --- | --- | --- | --- | --- | --- | --- | --- | --- | --- | --- | --- | --- | --- |
| iPLEX | rs11196218 | ACGTTGGATGAATAAGTGTGCAAACGAGGG | ACGTTGGATGCTCTTAACCAACATGGCTTG | 109 | R | 4569 | AAACCTTCTCGGGTG | G | 4816.2 | AAACCTTCTCGGGTGC | A | 4896.1 | AAACCTTCTCGGGTGT |
| iPLEX | rs822396 | ACGTTGGATGGTCTCTGGAGTTTTCTGGCT | ACGTTGGATGACAGTGTTCCCTTAGGGTAG | 95 | R | 4711.1 | tCTCCTCCCCCTTTGT | G | 4958.2 | tCTCCTCCCCCTTTGTC | A | 5038.2 | tCTCCTCCCCCTTTGTT |
| iPLEX | rs8191371 | ACGTTGGATGCTCACATTGCCAAAACCCTG | ACGTTGGATGCAGTTTTCGGCACTCATACC | 104 | F | 4744.1 | TCCTCCCTGTTCATCA | C | 4991.3 | TCCTCCCTGTTCATCAC | T | 5071.2 | TCCTCCCTGTTCATCAT |
| iPLEX | rs117535913 | ACGTTGGATGCCATCTTAGCCAAGATCTCC | ACGTTGGATGGTTTGCTGATCACTGCGTTC | 98 | R | 5164.4 | ACGTGCCAAGACCATTA | G | 5411.6 | ACGTGCCAAGACCATTAC | A | 5491.5 | ACGTGCCAAGACCATTAT |
| iPLEX | rs148962208 | ACGTTGGATGGGCATGAGCGCCTTCACCC | ACGTTGGATGGGTCTGCACGGATCACTTTG | 93 | R | 5196.4 | caCGGGTGAACCTCAGT | G | 5443.6 | caCGGGTGAACCTCAGTC | A | 5523.5 | caCGGGTGAACCTCAGTT |
| iPLEX | rs163184 | ACGTTGGATGAGGTGGTAAAGTGTCTGTGG | ACGTTGGATGTTTGCTCAGTAACGGACTGG | 136 | R | 5321.5 | CCTCTCTCTTTACTCCAA | G | 5568.7 | CCTCTCTCTTTACTCCAAC | T | 5592.7 | CCTCTCTCTTTACTCCAAA |
| iPLEX | rs16856187 | ACGTTGGATGCACACCGTGAATGAACCTTC | ACGTTGGATGCTCCAGAAGGCCATTTAGTG | 117 | R | 5357.5 | AACCCCATACTAAACTCC | C | 5644.7 | AACCCCATACTAAACTCCG | A | 5684.6 | AACCCCATACTAAACTCCT |
| iPLEX | rs13342232 | ACGTTGGATGTTCTCCCGCCGTCGAGTCTT | ACGTTGGATGTGAGGTGGAGGGTGATCGC | 171 | R | 5716.7 | cgcaCGCCTTGCAGCTTCT | G | 5963.9 | cgcaCGCCTTGCAGCTTCTC | A | 6043.8 | cgcaCGCCTTGCAGCTTCTT |
| iPLEX | rs822393 | ACGTTGGATGAAAGCATGACACGGAGCTTC | ACGTTGGATGTGTCAGCGTTCTCATGTTCC | 105 | F | 5749.7 | accCGGAGCTTCTCAGACT | C | 5996.9 | accCGGAGCTTCTCAGACTC | T | 6076.8 | accCGGAGCTTCTCAGACTT |
| iPLEX | rs202019027 | ACGTTGGATGCTGACCTTCAGAGACTCCAC | ACGTTGGATGGCAGGACAGCCCCATCTTC | 89 | F | 5862.8 | accaAGCGGAAGCTGGTAT | C | 6110 | accaAGCGGAAGCTGGTATC | T | 6189.9 | accaAGCGGAAGCTGGTATT |
| iPLEX | rs4402960 | ACGTTGGATGAGCAGTAAGGTAGGATGGAC | ACGTTGGATGCCTTATCTGGGGCATGTTTG | 88 | F | 5932.9 | GTAGGATGGACAGTAGATT | G | 6220.1 | GTAGGATGGACAGTAGATTG | T | 6260 | GTAGGATGGACAGTAGATTT |
| iPLEX | rs2224391 | ACGTTGGATGAAAATGGCAGCCTCCAGTCG | ACGTTGGATGGTCACCTGTAATTGTAGGCG | 114 | R | 6158 | ggatTCGCGCACAAGTGTTA | G | 6405.2 | ggatTCGCGCACAAGTGTTAC | C | 6445.2 | ggatTCGCGCACAAGTGTTAG |
| iPLEX | rs738409 | ACGTTGGATGTCACAGGCCTTGGTATGTTC | ACGTTGGATGAGAGAAAGCCGACTTACCAC | 109 | F | 6370.1 | CTTGGTATGTTCCTGCTTCAT | C | 6617.3 | CTTGGTATGTTCCTGCTTCATC | G | 6657.3 | CTTGGTATGTTCCTGCTTCATG |
| iPLEX | rs16861194 | ACGTTGGATGTTGAAGTTGGTGCTGGCATC | ACGTTGGATGGCAAGCCACACATTCTGATG | 90 | F | 6526.2 | ggggtCCTTGCTGGGGTCGTA | A | 6797.4 | ggggtCCTTGCTGGGGTCGTAA | G | 6813.4 | ggggtCCTTGCTGGGGTCGTAG |
| iPLEX | rs2304472 | ACGTTGGATGCTTTCTCAAAGTCCGATCCG | ACGTTGGATGAGAAATGGATGACTTCCTAC | 97 | R | 6573.3 | ccCCACTCCGATTTTGATCTTC | T | 6844.5 | ccCCACTCCGATTTTGATCTTCA | A | 6900.4 | ccCCACTCCGATTTTGATCTTCT |
| iPLEX | rs766756088 | ACGTTGGATGAGCCATGAGGGCTCGTTTCA | ACGTTGGATGTTCATGCCCTCCTTTCTCCG | 104 | F | 6718.4 | gaggtCTCGTTTCAGCTGCTCA | C | 6965.5 | gaggtCTCGTTTCAGCTGCTCAC | T | 7045.5 | gaggtCTCGTTTCAGCTGCTCAT |
| iPLEX | rs5973488 | ACGTTGGATGCCACATCAAGGCCAAATATC | ACGTTGGATGTGATGAGAGCCACTTCTCTG | 94 | R | 6760.4 | ggGGCCAAATATCATCTCTAGG | G | 7007.6 | ggGGCCAAATATCATCTCTAGGC | A | 7087.5 | ggGGCCAAATATCATCTCTAGGT |
| iPLEX | rs4737009 | ACGTTGGATGTCTGAGTGGCGATAAACCAG | ACGTTGGATGTCTGGGCACCACTGATAAGG | 100 | R | 6935.5 | gctATAAACCAGCCTGCCTTTCT | G | 7182.7 | gctATAAACCAGCCTGCCTTTCTC | A | 7262.6 | gctATAAACCAGCCTGCCTTTCTT |
| iPLEX | rs10882066 | ACGTTGGATGCACAGTCAAATCAGGATGGC | ACGTTGGATGTGCCCGGCCAATAGTTTGAG | 112 | R | 7127.7 | gTAGTACAGCATGGTAAATGTTA | G | 7374.8 | gTAGTACAGCATGGTAAATGTTAC | A | 7454.8 | gTAGTACAGCATGGTAAATGTTAT |
| iPLEX | rs775044452 | ACGTTGGATGTGGTGTAGAAGCCGATGATG | ACGTTGGATGGCTGCTAACTACCTGCAGTC | 96 | F | 7295.7 | ccgagGATGTTCTTCATGATCTCC | A | 7566.9 | ccgagGATGTTCTTCATGATCTCCA | G | 7583 | ccgagGATGTTCTTCATGATCTCCG |
| iPLEX | rs182052 | ACGTTGGATGCTTCTAAGATGCATGGAACC | ACGTTGGATGTCCACCTTACTGACCCTAAC | 111 | F | 7342.8 | ctgaATGGAACCATTCTGAATTTT | A | 7614 | ctgaATGGAACCATTCTGAATTTTA | G | 7630 | ctgaATGGAACCATTCTGAATTTTG |
| iPLEX | rs111678678 | ACGTTGGATGCATCATTGAGACCATGGAGC | ACGTTGGATGTGAGAGTAAGAGCCTTATGG | 97 | F | 7418.8 | ggggtAAGCAGTTTCACGCCAAGT | C | 7666 | ggggtAAGCAGTTTCACGCCAAGTC | T | 7745.9 | ggggtAAGCAGTTTCACGCCAAGTT |
| iPLEX | rs10923931 | ACGTTGGATGTTTTGCAGGAGTAGGGTCAC | ACGTTGGATGGACTGTGCCCATAATCTTAC | 112 | F | 7534.9 | aattTTGCTCCATCCTCTGGCTTCA | G | 7822.1 | aattTTGCTCCATCCTCTGGCTTCAG | T | 7862 | aattTTGCTCCATCCTCTGGCTTCAT |
| iPLEX | rs2021966 | ACGTTGGATGCTTTTGCAGCAACTATCAAG | ACGTTGGATGCCCTTTCCTATCTTATGAGC | 126 | R | 7708.1 | TGCAGCAACTATCAAGAAAGATATG | G | 7955.2 | TGCAGCAACTATCAAGAAAGATATGC | A | 8035.1 | TGCAGCAACTATCAAGAAAGATATGT |
| iPLEX | rs2300587 | ACGTTGGATGGGTCAGTTATATTTTGCCCC | ACGTTGGATGGCAGGGATGCTTGAGTTTAC | 101 | R | 7910.1 | ggggaCCCATTTCTTTTATCCTTTTG | T | 8181.3 | ggggaCCCATTTCTTTTATCCTTTTGA | C | 8197.3 | ggggaCCCATTTCTTTTATCCTTTTGG |
| iPLEX | rs6585205 | ACGTTGGATGCTCTTGCCATTCCTGGTTTC | ACGTTGGATGTATTGACCCAACTTGGTCCC | 94 | R | 7993.2 | ggggaCATTCCTGGTTTCATCTAAGT | G | 8240.4 | ggggaCATTCCTGGTTTCATCTAAGTC | T | 8264.4 | ggggaCATTCCTGGTTTCATCTAAGTA |
| iPLEX | rs7756992 | ACGTTGGATGGACAATTAATATTCCCCCCTG | ACGTTGGATGGCAAAAGGACTGATAATGAGC | 98 | F | 8133.3 | tacTATTCCCCCCTGTATTTTAGTTTT | A | 8404.5 | tacTATTCCCCCCTGTATTTTAGTTTTA | G | 8420.5 | tacTATTCCCCCCTGTATTTTAGTTTTG |
| iPLEX | rs12573128 | ACGTTGGATGCATTCTTCTGAGGTATGGAC | ACGTTGGATGCAGGTAACTTGCTCAAGAGG | 109 | R | 8353.5 | tGTATGGACTTAAATTAGCTAATTAGG | G | 8600.6 | tGTATGGACTTAAATTAGCTAATTAGGC | A | 8680.6 | tGTATGGACTTAAATTAGCTAATTAGGT |
| iPLEX | rs150536710 | ACGTTGGATGAGTGGTCACAGGTAAACTCC | ACGTTGGATGTCACACTGCTGTGTTTCTCC | 113 | F | 8524.5 | cccttGTCACAGGTAAACTCCATGTTGA | C | 8771.7 | cccttGTCACAGGTAAACTCCATGTTGAC | T | 8851.6 | cccttGTCACAGGTAAACTCCATGTTGAT |
| iPLEX | rs10965250 | ACGTTGGATGCACTCTTCCACTGACTTTGC | ACGTTGGATGCAAAATCTAAAGCGTTGTC | 158 | R | 8564.6 | TCTTATTTTAATAACTGATTTATTGTTG | G | 8811.8 | TCTTATTTTAATAACTGATTTATTGTTGC | A | 8891.7 | TCTTATTTTAATAACTGATTTATTGTTGT |
| iPLEX | rs767620772 | ACGTTGGATGTGTGGAAGGCGGAGAACAAG | ACGTTGGATGACATGACCCGTGGCTACCTG | 122 | F | 4535 | ATTGTGCCCTTGTCA | C | 4782.1 | ATTGTGCCCTTGTCAC | T | 4862.1 | ATTGTGCCCTTGTCAT |
| iPLEX | rs778093769 | ACGTTGGATGATTCAGGAGGCCTTACTACG | ACGTTGGATGTCACAAACTCATGGAGACGC | 106 | R | 4409.9 | CCCTGCTCACACCTC | T | 4681.1 | CCCTGCTCACACCTCA | C | 4697.1 | CCCTGCTCACACCTCG |
| iPLEX | rs2303929 | ACGTTGGATGTTCTTCCTCACAGCCAGAGC | ACGTTGGATGTGAAGTTCGTCTTCCTCCTG | 98 | F | 4610 | AGAGCTTGGGCCCTG | A | 4881.2 | AGAGCTTGGGCCCTGA | G | 4897.2 | AGAGCTTGGGCCCTGG |
| iPLEX | rs225014 | ACGTTGGATGTCTTCTCCTGGGTACCATTG | ACGTTGGATGATTCCAGTGTGGTGCATGTC | 89 | F | 4775.1 | TTGTCACCTCCTTCTG | C | 5022.3 | TTGTCACCTCCTTCTGC | T | 5102.2 | TTGTCACCTCCTTCTGT |
| iPLEX | rs533349497 | ACGTTGGATGATTGGTGGGTTTGTAGCTCC | ACGTTGGATGTTTCAGGTCTCCACCATCGG | 119 | R | 4809.1 | CAGTCTCTTGCTCACG | G | 5056.3 | CAGTCTCTTGCTCACGC | A | 5136.2 | CAGTCTCTTGCTCACGT |
| iPLEX | RS9368197 | ACGTTGGATGGAAGCTATAAACATAGGCAC | ACGTTGGATGAAAGTAGGCCCTGGGTTATC | 97 | F | 4946.2 | AGGCACAAGGTTATGT | G | 5233.4 | AGGCACAAGGTTATGTG | T | 5273.3 | AGGCACAAGGTTATGTT |
| iPLEX | rs6413453 | ACGTTGGATGGGGTCCCTCAGATTAAACTC | ACGTTGGATGGTTCCAGCCTTCTTGATCAG | 117 | R | 5330.5 | TCTAATCCCCTCACCTAT | G | 5577.7 | TCTAATCCCCTCACCTATC | A | 5657.6 | TCTAATCCCCTCACCTATT |
| iPLEX | rs201588284 | ACGTTGGATGACCAGCATGTGGCGATCGAG | ACGTTGGATGATTGCTTTCTGCAGCACAAC | 126 | R | 5451.6 | CCGAGCTCAGTGACTTTC | G | 5698.7 | CCGAGCTCAGTGACTTTCC | A | 5778.6 | CCGAGCTCAGTGACTTTCT |
| iPLEX | RS937301 | ACGTTGGATGTTACAGACGTGATTCATCGC | ACGTTGGATGCTTCCTTTACAGTTGCCCTC | 88 | R | 5485.6 | GCGCCTGGCCTACAAGTA | G | 5732.8 | GCGCCTGGCCTACAAGTAC | A | 5812.7 | GCGCCTGGCCTACAAGTAT |
| iPLEX | rs189506473 | ACGTTGGATGACGTGTCTCAAACCAGTGTC | ACGTTGGATGTCCAGGTGTTTCTTGCTATC | 101 | F | 5614.7 | ccccGCCCTTCCATCCAAA | A | 5885.9 | ccccGCCCTTCCATCCAAAA | G | 5901.9 | ccccGCCCTTCCATCCAAAG |
| iPLEX | rs1801282 | ACGTTGGATGTGTATCAGTGAAGGAATCGC | ACGTTGGATGCAAACCCCTATTCCATGCTG | 102 | R | 5843.8 | aTGAAGGAATCGCTTTCTG | G | 6091 | aTGAAGGAATCGCTTTCTGC | C | 6131 | aTGAAGGAATCGCTTTCTGG |
| iPLEX | rs72696119 | ACGTTGGATGTCGCTGGTGCACTTCTCTCT | ACGTTGGATGTGTCTGTATGCTCTCTCGAC | 110 | R | 5951.9 | tTGCACTTCTCTCTCTTTCT | G | 6199.1 | tTGCACTTCTCTCTCTTTCTC | C | 6239.1 | tTGCACTTCTCTCTCTTTCTG |
| iPLEX | rs2971672 | ACGTTGGATGAACCCATGATCCAGAGTGAG | ACGTTGGATGGCAGGCCATAGGTAGTATTG | 125 | R | 5997.9 | cgGCCCTCTAAATAATCCCT | C | 6285.1 | cgGCCCTCTAAATAATCCCTG | A | 6325 | cgGCCCTCTAAATAATCCCTT |
| iPLEX | rs1801159 | ACGTTGGATGTGCGCTAGCAAGACCAAAAG | ACGTTGGATGCTCCTATTGATCTGGTGGAC | 97 | F | 6168.1 | AGCAAGACCAAAAGGATTTA | C | 6415.2 | AGCAAGACCAAAAGGATTTAC | T | 6495.2 | AGCAAGACCAAAAGGATTTAT |
| iPLEX | rs3792267 | ACGTTGGATGAAGTCAAGGCTTAGCCTCAC | ACGTTGGATGTTTCTGTGTGTGGGCAGAGG | 103 | R | 6375.2 | aggaTTAGCCTCACCTTCAAA | G | 6622.4 | aggaTTAGCCTCACCTTCAAAC | A | 6702.3 | aggaTTAGCCTCACCTTCAAAT |
| iPLEX | rs1410961 | ACGTTGGATGAGGTTGCACAGGTCTCCAAG | ACGTTGGATGAGCTTCCTTCAGTTTGCCAG | 103 | F | 6537.2 | gggtAGGCTCTGGAGAAGACC | C | 6784.4 | gggtAGGCTCTGGAGAAGACCC | T | 6864.3 | gggtAGGCTCTGGAGAAGACCT |
| iPLEX | rs1387153 | ACGTTGGATGCAACTCTGTCTGTGGAATGC | ACGTTGGATGCTCTCTCTAGAGCTCACAAC | 118 | R | 6654.4 | atCAAATTAACTTACTGCCCTG | G | 6901.5 | atCAAATTAACTTACTGCCCTGC | T | 6925.6 | atCAAATTAACTTACTGCCCTGA |
| iPLEX | rs7895340 | ACGTTGGATGGTGTGACAGTTCTAGACACC | ACGTTGGATGTGGACCATGCTGGGAAATTC | 106 | F | 6752.4 | TTCTAGACACCTAGAGAGTAAA | A | 7023.6 | TTCTAGACACCTAGAGAGTAAAA | G | 7039.6 | TTCTAGACACCTAGAGAGTAAAG |
| iPLEX | rs764256858 | ACGTTGGATGAGGAGCCTCTTGACTTGACA | ACGTTGGATGTCTTACTTACCTCTGTTGAC | 105 | R | 6823.5 | CTGGGACTAAGAGAATTATATG | G | 7070.7 | CTGGGACTAAGAGAATTATATGC | A | 7150.6 | CTGGGACTAAGAGAATTATATGT |
| iPLEX | rs776254567 | ACGTTGGATGGAACTCAATTGCTATGCAGG | ACGTTGGATGACCTTTTCAAGGCTATATCC | 95 | R | 6831.4 | tgggaAGATGCTTGCCTGTAAG | T | 7102.7 | tgggaAGATGCTTGCCTGTAAGA | C | 7118.7 | tgggaAGATGCTTGCCTGTAAGG |
| iPLEX | rs1804764 | ACGTTGGATGACTGCCCCATAGAGACCCAA | ACGTTGGATGCTCAGCTTCCATCCATCTTC | 109 | R | 6984.6 | tcctCATAGAGACCCAAGTTCTG | G | 7231.7 | tcctCATAGAGACCCAAGTTCTGC | T | 7255.8 | tcctCATAGAGACCCAAGTTCTGA |
| iPLEX | rs3779536 | ACGTTGGATGTCTTGCATGCCTCAAAGGTG | ACGTTGGATGATACACAGACATCCCAAGGC | 105 | R | 7185.7 | ggacAAGGTGAGGTAGGTTCCTG | G | 7432.9 | ggacAAGGTGAGGTAGGTTCCTGC | T | 7456.9 | ggacAAGGTGAGGTAGGTTCCTGA |
| iPLEX | rs138772212 | ACGTTGGATGCCCAGATCACTTACAATGGC | ACGTTGGATGTGTTCAGACAGAACGCCAAG | 91 | R | 7345.8 | gggagTACAATGGCCTTTTCCCGC | G | 7593 | gggagTACAATGGCCTTTTCCCGCC | A | 7672.9 | gggagTACAATGGCCTTTTCCCGCT |
| iPLEX | rs780094 | ACGTTGGATGCCCGGCCTCAACAAATGTAT | ACGTTGGATGGATGGTGACTTATTCTGCTC | 107 | F | 7377.9 | taAACAAATGTATTGATCAGCAAA | C | 7625 | taAACAAATGTATTGATCAGCAAAC | T | 7705 | taAACAAATGTATTGATCAGCAAAT |
| iPLEX | rs4845617 | ACGTTGGATGCTCTCTACACACACTGCGAG | ACGTTGGATGTCATGTGCGAGTGGGAAGTC | 119 | R | 7553.9 | gggctCTCGGCTCCTCTCCCTCTGG | G | 7801.1 | gggctCTCGGCTCCTCTCCCTCTGGC | C | 7841.1 | gggctCTCGGCTCCTCTCCCTCTGGG |
| iPLEX | rs10811661 | ACGTTGGATGAGATCAGGAGGGTAATAGAC | ACGTTGGATGGTCAATAAGCGTTCTTGCCC | 103 | R | 7737 | ggatGGTAATAGACTTACTGTCATG | T | 8008.3 | ggatGGTAATAGACTTACTGTCATGA | C | 8024.3 | ggatGGTAATAGACTTACTGTCATGG |
| iPLEX | rs1501299 | ACGTTGGATGTCCCTGTGTCTAGGCCTTAG | ACGTTGGATGCTCTTTCATCACAGACCTCC | 98 | R | 7970.2 | ccCTAGGCCTTAGTTAATAATGAATG | G | 8217.4 | ccCTAGGCCTTAGTTAATAATGAATGC | T | 8241.4 | ccCTAGGCCTTAGTTAATAATGAATGA |
| iPLEX | rs17032850 | ACGTTGGATGCTATCCACAGACTCTTTGGG | ACGTTGGATGTAACAGGCACCAACTGAGTC | 99 | R | 8059.3 | ggcatTGGACTCTAGGTTAAGAAATA | G | 8306.4 | ggcatTGGACTCTAGGTTAAGAAATAC | C | 8346.5 | ggcatTGGACTCTAGGTTAAGAAATAG |
| iPLEX | rs8192678 | ACGTTGGATGTACTGAAATCACTGTCCCTC | ACGTTGGATGGAACAAGCACTTCGGTCATC | 106 | F | 8130.3 | tacttAAATCACTGTCCCTCAGTTCAC | C | 8377.5 | tacttAAATCACTGTCCCTCAGTTCACC | T | 8457.4 | tacttAAATCACTGTCCCTCAGTTCACT |
| iPLEX | rs160632 | ACGTTGGATGGATTCTTCTAGACAGTTCCG | ACGTTGGATGTTCAGATGGAGAAGTGGCAG | 98 | F | 8168.3 | agtgCCGTTCACTTTCATTTTCTGACC | C | 8415.5 | agtgCCGTTCACTTTCATTTTCTGACCC | T | 8495.4 | agtgCCGTTCACTTTCATTTTCTGACCT |
| iPLEX | rs6517656 | ACGTTGGATGTCAGGCTGCTAATCACTAAC | ACGTTGGATGTGCTAATTGCTGCAATAAGG | 101 | F | 8556.6 | agtCTGCTAATCACTAACTAGATGAATC | A | 8827.8 | agtCTGCTAATCACTAACTAGATGAATCA | G | 8843.8 | agtCTGCTAATCACTAACTAGATGAATCG |
| iPLEX | rs6415788 | ACGTTGGATGTGTTCAAGACCGAACGCCTG | ACGTTGGATGTGCGCATGGGCATGGTAAG | 141 | R | 8648.6 | gactaCGCTCCCTCCTCTGCCGCCGCCCC | G | 8895.8 | gactaCGCTCCCTCCTCTGCCGCCGCCCCC | T | 8919.8 | gactaCGCTCCCTCCTCTGCCGCCGCCCCA |
| iPLEX | rs7747752 | ACGTTGGATGCTCCCCTAAGATCAATATCC | ACGTTGGATGCTCTCTTTCTCCCCTTGTTG | 102 | R | 8702.7 | ccccgCAATATCCTGATACTCTACTCACA | G | 8949.9 | ccccgCAATATCCTGATACTCTACTCACAC | C | 8989.9 | ccccgCAATATCCTGATACTCTACTCACAG |

Table S2. Results of clinical relevance by damaging or probably damaging prediction.

| SNP | Group | type | Chr | fisher P | mut | Func | Gene | Func | 1000g2015aug_eas | ExAC_EAS | gnomAD_exome_EAS |
| --- | --- | --- | --- | --- | --- | --- | --- | --- | --- | --- | --- |
| rs201117022 | High | SNP | chr9 | 0.010835913 | C/T | exonic | AQP7 | nonsynonymous SNV | . | 0.0177 | 0.0002 |
| rs201994479 | High | SNP | chr12 | 0.03250774 | G/T | exonic | PRB2 | nonsynonymous SNV | . | 0.0001 | 0.00006047 |
| rs3744692 | High | SNP | chr17 | 0.03250774 | C/T | exonic | ALDH3A1 | nonsynonymous SNV | 0.0625 | 0.0584 | 0.0568 |
| rs537664066 | High | SNP | chr3 | 0.03250774 | C/A | exonic | MUC4 | nonsynonymous SNV | . | 0 | 0.0001 |
| rs144896967 | High | SNP | chr16 | 0.047987616 | C/T | exonic | NPIPA5 | nonsynonymous SNV | 0.0268 | 0.0003 | 0.0005 |

Table S3. Top 50 of 1770 significantly differentiated SNVs between cases and controls (P < 0.05)

| Mut_type | CHROM | POS | SNV | Share_num | Mut | Func | Gene | Fisher P |
| --- | --- | --- | --- | --- | --- | --- | --- | --- |
| SNP | chr12 | 54394497 | rs2241820 | 10/10 | C/T | exonic | HOXC9 | 0.000119076 |
| SNP | chr19 | 4229635 | rs400089 | 8/9 | A/G | intronic | EBI3 | 0.000335578 |
| SNP | chr22 | 31673111 | rs3747153 | 6/9 | A/G | exonic | LIMK2 | 0.000714456 |
| SNP | chr22 | 31673116 | rs3747154 | 6/9 | A/G | exonic | LIMK2 | 0.000714456 |
| SNP | chr22 | 31685256 | rs2073861 | 6/9 | C/G | intronic | PIK3IP1 | 0.000714456 |
| SNP | chr22 | 31722886 | rs2240424 | 6/9 | T/G | exonic | PATZ1 | 0.000714456 |
| SNP | chr1 | 111441829 | rs1803259 | 10/7 | C/T | UTR3 | CD53 | 0.000714456 |
| SNP | chr1 | 161479745 | rs1801274 | 4/10 | A/G | exonic | FCGR2A | 0.000714456 |
| InDel | chr12 | 19626313 | rs10631217 | 0/8 | T/TAA | intronic | AEBP2 | 0.000714456 |
| SNP | chr12 | 19626318 | rs57423687 | 0/8 | T/A | intronic | AEBP2 | 0.000714456 |
| SNP | chr2 | 69040500 | rs2280310 | 10/6 | G/A | exonic | ARHGAP25 | 0.000714456 |
| SNP | chr5 | 160763616 | rs2303055 | 8/0 | T/G | intronic | GABRB2 | 0.000714456 |
| SNP | chr7 | 102240301 | rs370047247 | 0/8 | A/T | intronic | RASA4;RASA4B | 0.000714456 |
| InDel | chr12 | 19626314 | rs546038420 | 0/7 | T/TTA | intronic | AEBP2 | 0.000714456 |
| SNP | chr12 | 53926262 | rs7307804 | 7/8 | G/A | intronic | ATF7 | 0.001093334 |
| SNP | chr15 | 51529112 | rs700518 | 5/10 | T/C | exonic | CYP19A1 | 0.001093334 |
| SNP | chr2 | 111774116 | rs4849340 | 9/10 | G/A | intronic | ACOXL | 0.001093334 |
| SNP | chr3 | 38926912 | rs6599266 | 9/2 | C/G | intronic | SCN11A | 0.001093334 |
| SNP | chr4 | 153332380 | rs12644477 | 9/1 | T/G | UTR3 | FBXW7 | 0.001093334 |
| SNP | chr5 | 96518792 | rs3734010 | 10/6 | T/G | intronic | RIOK2 | 0.001093334 |
| SNP | chr9 | 33750748 | rs10971678 | 10/4 | G/T | intronic | PRSS3 | 0.001093334 |
| SNP | chr12 | 69646914 | rs2305641 | 1/8 | G/A | exonic | CPSF6 | 0.001309836 |
| SNP | chr3 | 155628877 | rs10936021 | 8/1 | T/G | intronic | GMPS | 0.001309836 |
| SNP | chr3 | 155639959 | rs3772117 | 8/1 | A/G | intronic | GMPS | 0.001309836 |
| SNP | chr2 | 75923413 | rs7560262 | 1/9 | T/C | exonic | GCFC2 | 0.001580463 |
| SNP | chr2 | 127977550 | rs6430931 | 10/8 | G/C | intergenic | CYP27C1;ERCC3 | 0.001580463 |
| SNP | chrX | 35820265 | rs5928990 | 8/9 | C/T | UTR5 | MAGEB16 | 0.001905216 |
| SNP | chrX | 35820425 | rs1410961 | 8/9 | C/T | exonic | MAGEB16 | 0.001905216 |
| SNP | chrX | 35820696 | rs1410962 | 8/9 | G/A | exonic | MAGEB16 | 0.001905216 |
| SNP | chrX | 35820795 | rs5973488 | 8/9 | A/G | exonic | MAGEB16 | 0.001905216 |
| SNP | chrX | 35821055 | rs4829390 | 8/9 | A/G | exonic | MAGEB16 | 0.001905216 |
| SNP | chrX | 35821056 | rs4829391 | 8/9 | T/A | exonic | MAGEB16 | 0.001905216 |
| SNP | chrX | 35821127 | rs4829392 | 8/9 | C/T | exonic | MAGEB16 | 0.001905216 |
| SNP | chrX | 35821302 | rs4829393 | 8/9 | C/G | UTR3 | MAGEB16 | 0.001905216 |
| SNP | chrX | 35821308 | rs4829394 | 8/9 | C/T | UTR3 | MAGEB16 | 0.001905216 |
| SNP | chr4 | 187093018 | rs4241814 | 9/2 | G/T | intronic | FAM149A | 0.001970166 |
| SNP | chr10 | 119036625 | rs363279 | 9/3 | T/C | intronic | SLC18A2 | 0.002186668 |
| SNP | chr15 | 51783981 | rs12101858 | 3/10 | G/C | intronic | DMXL2 | 0.002186668 |
| SNP | chr14 | 55907172 | rs8019270 | 5/10 | C/G | exonic | TBPL2 | 0.002457295 |
| SNP | chr16 | 69008037 | rs3743678 | 8/1 | C/T | exonic | TANGO6 | 0.002500595 |
| SNP | chr3 | 9406836 | rs1054975 | 7/9 | T/C | exonic | THUMPD3 | 0.002500595 |
| SNP | chr3 | 9407022 | rs2648536 | 7/9 | T/C | intronic | THUMPD3 | 0.002500595 |
| SNP | chr5 | 96498783 | rs8654 | 10/5 | G/A | exonic | RIOK2 | 0.002500595 |
| SNP | chr5 | 96503523 | rs160632 | 10/5 | C/T | exonic | RIOK2 | 0.002500595 |
| InDel | chr1 | 149239970 | rs367955970 | 0/7 | TG/T | intergenic | NBPF25P;FAM231D | 0.003095975 |
| SNP | chr1 | 240656797 | rs11806449 | 5/10 | G/A | ncRNA_intronic | MIR1273E | 0.003095975 |
| SNP | chr1 | 248814052 | rs28533004 | 7/10 | T/A | exonic | OR2T27 | 0.003095975 |
| InDel | chr11 | 102709461 | rs3025072 | 0/7 | CAG/C | intronic | MMP3 | 0.003095975 |
| SNP | chr12 | 40882632 | rs200982622 | 10/3 | A/G | exonic | MUC19 | 0.003095975 |
| SNP | chr15 | 99901749 | rs3817147 | 10/4 | C/G | intronic | LRRC28 | 0.003095975 |

Table S4. A total of 308 exonic missense SNVs were annotated on 246 genes (P < 0.05).

| ABCA7,ABTB2,ACSF3,ACSM3,ADAMTS16,ADGRE1,ADGRG4,AFF3,ALDH1B1,ALDH3A1,AMOTL2,AMZ1,ANAPC1,ANKDD1B,ANKIB1,ANXA8L1,AQP7,ARHGAP25,ARHGEF28,ARMS2,BANK1,BCAS1,BCAS3,BCDIN3D,BEST4,BMP2K,BRAT1,BTN3A2,C10orf95,C11orf21,C6orf183,C8A,CALML5,CCDC144NL,CCDC180,CCDC33,CD164L2,CDC6,CELA3A,CEP112,CEP170,CEP85,CFAP47,CHGB,CHRNA2,CNBD2,COL22A1,CROCC2,CWC27,CYB5R2,CYP39A1,CYP3A7,CYP3A7-CYP3A51P,CYP4V2,DCAF13,DISC1,DNAAF3,DNAH1,DNAH17,DNHD1,DPYD,DRAXIN,DSCAML1,DTHD1,DYNC2H1,EARS2,EEF2K,EFCAB8,EPS8L1,EXD3,FANCE,FAT2,FBXO16,FCGBP,FCGR2A,FKBP10,FLCN,FLVCR1,FSCN3,GAL3ST4,GCFC2,GDF5,GDF5OS,GGA2,GRIN3B,GTPBP10,H1FNT,HASPIN,HEATR5A,HGC6.3,HOXC11,IFT140,IL15RA,IQGAP2,IQSEC3,ITGAE,ITSN2,KBTBD13,KCTD3,KIAA0319,KRT25,KRT40,KRTAP10-5,KRTAP7-1,LECT2,LILRB1,LIMK2,LMBR1,LNPEP,LOC100129307,LRRC1,LRRC41,LRRC56,LRRC74A,LYRM4,MADCAM1,MAGEB16,MAGEC1,METTL18,MLX,MMP15,MRGPRE,MROH2A,MTERF1,MTHFD1,MTMR4,MUC12,MUC15,MUC16,MUC17,MUC20,MUC3A,MUC4,MZF1,NBPF10,NBPF19,NBPF20,NCKAP5L,NID2,NLRP10,NLRP12,NOTCH2NL,NPC1,NPIPA5,NSUN4,NUP210,NUTM2B,OR10A2,OR10A4,OR11G2,OR2T27,OR4D11,OR51A2,OR51A7,OR52N2,OR52N5,OR56B1,OR6A2,OR8B3,P2RY4,P3H4,PATZ1,PCDHGA11,PCDHGA7,PCDHGB2,PDE4DIP,PDIA6,PGLYRP3,PIGN,PIGQ,PLA2G7,PMS2,PNLIPRP1,POLN,POM121L2,POTEC,POU5F1B,PRB2,PROK1,PRSS12,PSCA,RAD23B,RBM47,RIMBP3,RIOK2,RTL4,SDCCAG8,SENP7,SERPINB8,SFTPA1,SFTPA2,SLC16A9,SLC25A5,SLC2A9,SLC35G4,SLC4A2,SLC6A12,SLC9C1,SLCO5A1,SMCHD1,SNED1,SOAT2,SON,SPARCL1,SPATA17,SPOCD1,SRA1,SSPO,SUCLG2,TACC3,TARP,TBC1D26,TBPL2,TCP10,TCTEX1D1,TDRD6,TEKT5,TELO2,TGOLN2,THTPA,THUMPD1,TIRAP,TMEM247,TMPRSS6,TNN,TOR2A,TRPA1,TTLL8,TTN,UQCC1,USP17L8,USP29,WDR18,WDR27,WDR31,WNT16,ZFHX2,ZNF19,ZNF208,ZNF225,ZNF365,ZNF44,ZNF568,ZNF625,ZNF728,ZXDA |
| --- |

Table S5. Gene ontology and pathway analysis results of 330 genes of 467 significant exonic missense variants (P < 0.05)

| Category | Description | counts | % | Hits | Best log P |
| --- | --- | --- | --- | --- | --- |
| GO Biological Processes | cilium movement involved in cell motility | 8 | 3.29 | DNAH17\|DNAH1\|DYNC2H1\|DNHD1\|TEKT5\|TTLL8\|SLC9C1\|CFAP47 | -5.33 |
| GO Biological Processes | cilium or flagellum-dependent cell motility | 8 | 3.29 | DNAH17\|DNAH1\|DYNC2H1\|DNHD1\|TEKT5\|TTLL8\|SLC9C1\|CFAP47 | -5.33 |
| GO Biological Processes | cilium-dependent cell motility | 8 | 3.29 | DNAH17\|DNAH1\|DYNC2H1\|DNHD1\|TEKT5\|TTLL8\|SLC9C1\|CFAP47 | -5.33 |
| GO Biological Processes | cilium movement | 8 | 3.29 | DNAH17\|DNAH1\|DYNC2H1\|DNHD1\|TEKT5\|TTLL8\|SLC9C1\|CFAP47 | -5.33 |
| GO Biological Processes | cilium assembly | 10 | 4.12 | DNAH17\|IFT140\|DNAH1\|DISC1\|DYNC2H1\|DNHD1\|TEKT5\|TTLL8\|CFAP47\|DNAAF3 | -5.33 |
| GO Biological Processes | cilium organization | 10 | 4.12 | DNAH17\|IFT140\|DNAH1\|DISC1\|DYNC2H1\|DNHD1\|TEKT5\|TTLL8\|CFAP47\|DNAAF3 | -5.33 |
| GO Biological Processes | microtubule-based movement | 10 | 4.12 | DNAH17\|IFT140\|DNAH1\|DYNC2H1\|DNHD1\|TEKT5\|TTLL8\|DYNLT5\|SLC9C1\|CFAP47 | -5.33 |
| GO Biological Processes | motile cilium assembly | 4 | 1.65 | DNAH1\|DNHD1\|CFAP47\|DNAAF3 | -5.33 |
| GO Biological Processes | plasma membrane bounded cell projection assembly | 10 | 4.12 | DNAH17\|IFT140\|DNAH1\|DISC1\|DYNC2H1\|DNHD1\|TEKT5\|TTLL8\|CFAP47\|DNAAF3 | -5.33 |
| GO Biological Processes | flagellated sperm motility | 5 | 2.06 | DNAH1\|DNHD1\|TTLL8\|SLC9C1\|CFAP47 | -5.33 |
| GO Biological Processes | sperm motility | 5 | 2.06 | DNAH1\|DNHD1\|TTLL8\|SLC9C1\|CFAP47 | -5.33 |
| GO Biological Processes | cell projection assembly | 10 | 4.12 | DNAH17\|IFT140\|DNAH1\|DISC1\|DYNC2H1\|DNHD1\|TEKT5\|TTLL8\|CFAP47\|DNAAF3 | -5.33 |
| GO Biological Processes | microtubule bundle formation | 5 | 2.06 | DNAH17\|DNAH1\|NCKAP5L\|CFAP47\|DNAAF3 | -5.33 |
| GO Biological Processes | axonemal dynein complex assembly | 3 | 1.23 | DNAH17\|DNAH1\|DNAAF3 | -5.33 |
| GO Biological Processes | sperm flagellum assembly | 3 | 1.23 | DNAH1\|DNHD1\|CFAP47 | -5.33 |
| GO Biological Processes | axoneme assembly | 4 | 1.65 | DNAH17\|DNAH1\|CFAP47\|DNAAF3 | -5.33 |
| GO Biological Processes | embryonic limb morphogenesis | 7 | 2.88 | HOXC11\|AFF3\|GDF5\|IFT140\|FLVCR1\|LMBR1\|DYNC2H1 | -4.22 |
| GO Biological Processes | embryonic appendage morphogenesis | 7 | 2.88 | HOXC11\|AFF3\|GDF5\|IFT140\|FLVCR1\|LMBR1\|DYNC2H1 | -4.22 |
| GO Biological Processes | appendage morphogenesis | 7 | 2.88 | HOXC11\|AFF3\|GDF5\|IFT140\|FLVCR1\|LMBR1\|DYNC2H1 | -4.22 |
| GO Biological Processes | limb morphogenesis | 7 | 2.88 | HOXC11\|AFF3\|GDF5\|IFT140\|FLVCR1\|LMBR1\|DYNC2H1 | -4.22 |
| GO Biological Processes | appendage development | 7 | 2.88 | HOXC11\|AFF3\|GDF5\|IFT140\|FLVCR1\|LMBR1\|DYNC2H1 | -4.22 |
| GO Biological Processes | limb development | 7 | 2.88 | HOXC11\|AFF3\|GDF5\|IFT140\|FLVCR1\|LMBR1\|DYNC2H1 | -4.22 |
| GO Biological Processes | embryonic skeletal system morphogenesis | 5 | 2.06 | HOXC11\|MTHFD1\|IFT140\|FLVCR1\|DSCAML1 | -4.22 |
| GO Biological Processes | embryonic digit morphogenesis | 4 | 1.65 | HOXC11\|IFT140\|FLVCR1\|LMBR1 | -4.22 |
| GO Biological Processes | embryonic morphogenesis | 12 | 4.94 | HOXC11\|AFF3\|MMP15\|MTHFD1\|GDF5\|IFT140\|FLVCR1\|WNT16\|DSCAML1\|LMBR1\|DYNC2H1\|ZNF568 | -4.22 |
| GO Biological Processes | embryonic skeletal system development | 5 | 2.06 | HOXC11\|MTHFD1\|IFT140\|FLVCR1\|DSCAML1 | -4.22 |
| GO Biological Processes | detection of stimulus | 16 | 6.58 | TTN\|OR6A2\|TRPA1\|DISC1\|PGLYRP3\|OR51A7\|OR4D11\|OR10A4\|OR10A2\|OR56B1\|OR52N5\|OR52N2\|OR8B3\|OR11G2\|OR51A2\|OR2T27 | -4.21 |
| GO Biological Processes | detection of chemical stimulus involved in sensory perception | 13 | 5.35 | OR6A2\|TRPA1\|OR51A7\|OR4D11\|OR10A4\|OR10A2\|OR56B1\|OR52N5\|OR52N2\|OR8B3\|OR11G2\|OR51A2\|OR2T27 | -4.21 |
| GO Biological Processes | detection of stimulus involved in sensory perception | 14 | 5.76 | OR6A2\|TRPA1\|DISC1\|OR51A7\|OR4D11\|OR10A4\|OR10A2\|OR56B1\|OR52N5\|OR52N2\|OR8B3\|OR11G2\|OR51A2\|OR2T27 | -4.21 |
| GO Biological Processes | detection of chemical stimulus involved in sensory perception of smell | 12 | 4.94 | OR6A2\|OR51A7\|OR4D11\|OR10A4\|OR10A2\|OR56B1\|OR52N5\|OR52N2\|OR8B3\|OR11G2\|OR51A2\|OR2T27 | -4.21 |
| GO Biological Processes | detection of chemical stimulus | 13 | 5.35 | OR6A2\|TRPA1\|OR51A7\|OR4D11\|OR10A4\|OR10A2\|OR56B1\|OR52N5\|OR52N2\|OR8B3\|OR11G2\|OR51A2\|OR2T27 | -4.21 |
| GO Biological Processes | sensory perception of smell | 12 | 4.94 | OR6A2\|OR51A7\|OR4D11\|OR10A4\|OR10A2\|OR56B1\|OR52N5\|OR52N2\|OR8B3\|OR11G2\|OR51A2\|OR2T27 | -4.21 |
| GO Biological Processes | sensory perception of chemical stimulus | 13 | 5.35 | OR6A2\|TRPA1\|OR51A7\|OR4D11\|OR10A4\|OR10A2\|OR56B1\|OR52N5\|OR52N2\|OR8B3\|OR11G2\|OR51A2\|OR2T27 | -4.21 |
| GO Biological Processes | microtubule cytoskeleton organization | 14 | 5.76 | LIMK2\|SON\|DNAH17\|PDE4DIP\|TACC3\|SDCCAG8\|DCAF13\|DNAH1\|DISC1\|NCKAP5L\|CEP85\|SPATA17\|CFAP47\|DNAAF3 | -4.00 |
| GO Biological Processes | cell-substrate adhesion | 7 | 2.88 | FAT2\|ITGAE\|MUC4\|MADCAM1\|NID2\|SNED1\|TNN | -3.11 |
| GO Biological Processes | cell-matrix adhesion | 6 | 2.47 | ITGAE\|MUC4\|MADCAM1\|NID2\|SNED1\|TNN | -3.11 |
| GO Biological Processes | male gamete generation | 14 | 5.76 | LIMK2\|RAD23B\|SLC4A2\|PATZ1\|DNAH1\|RIMBP3\|SPOCD1\|CNBD2\|DNHD1\|ADAMTS16\|TDRD6\|SLC9C1\|CFAP47\|H1-7 | -3.08 |
| GO Biological Processes | spermatogenesis | 13 | 5.35 | LIMK2\|RAD23B\|SLC4A2\|PATZ1\|DNAH1\|RIMBP3\|SPOCD1\|CNBD2\|DNHD1\|TDRD6\|SLC9C1\|CFAP47\|H1-7 | -3.08 |
| GO Biological Processes | regulation of sister chromatid segregation | 5 | 2.06 | CDC6\|TACC3\|RIOK2\|ANAPC1\|HASPIN | -2.81 |
| GO Biological Processes | regulation of chromosome segregation | 5 | 2.06 | CDC6\|TACC3\|RIOK2\|ANAPC1\|HASPIN | -2.81 |
| GO Biological Processes | cholesterol efflux | 3 | 1.23 | NPC1\|SOAT2\|ABCA7 | -2.80 |
| GO Biological Processes | sterol metabolic process | 5 | 2.06 | NPC1\|SOAT2\|CYP39A1\|CYB5R2\|CYP4V2 | -2.80 |
| GO Biological Processes | plasma lipoprotein particle organization | 3 | 1.23 | PLA2G7\|SOAT2\|ABCA7 | -2.80 |
| GO Biological Processes | protein-lipid complex organization | 3 | 1.23 | PLA2G7\|SOAT2\|ABCA7 | -2.80 |
| GO Biological Processes | cholesterol homeostasis | 4 | 1.65 | NPC1\|ACSM3\|SOAT2\|CYP39A1 | -2.80 |
| GO Biological Processes | sterol homeostasis | 4 | 1.65 | NPC1\|ACSM3\|SOAT2\|CYP39A1 | -2.80 |
| GO Biological Processes | regulation of cilium assembly | 4 | 1.65 | LIMK2\|IFT140\|SDCCAG8\|ADAMTS16 | -2.52 |
| GO Biological Processes | regulation of plasma membrane bounded cell projection organization | 12 | 4.94 | IL15RA\|LIMK2\|IFT140\|KIAA0319\|SDCCAG8\|ZNF365\|DISC1\|EEF2K\|EPS8L1\|TNN\|ADAMTS16\|DRAXIN | -2.52 |
| GO Biological Processes | regulation of cell projection organization | 12 | 4.94 | IL15RA\|LIMK2\|IFT140\|KIAA0319\|SDCCAG8\|ZNF365\|DISC1\|EEF2K\|EPS8L1\|TNN\|ADAMTS16\|DRAXIN | -2.52 |
| GO Biological Processes | supramolecular fiber organization | 12 | 4.94 | LIMK2\|TTN\|ARHGAP25\|P3H4\|IQGAP2\|EEF2K\|FSCN3\|NCKAP5L\|FKBP10\|KRT40\|KRT25\|KBTBD13 | -2.50 |
| GO Biological Processes | bone remodeling | 3 | 1.23 | GDF5\|P3H4\|WNT16 | -2.28 |
| GO Biological Processes | extracellular matrix organization | 7 | 2.88 | MMP15\|P3H4\|NID2\|FKBP10\|TMPRSS6\|COL22A1\|ADAMTS16 | -2.16 |
| GO Biological Processes | extracellular structure organization | 7 | 2.88 | MMP15\|P3H4\|NID2\|FKBP10\|TMPRSS6\|COL22A1\|ADAMTS16 | -2.16 |
| GO Biological Processes | external encapsulating structure organization | 7 | 2.88 | MMP15\|P3H4\|NID2\|FKBP10\|TMPRSS6\|COL22A1\|ADAMTS16 | -2.16 |
| GO Cellular Components | Golgi lumen | 7 | 2.88 | MUC3A\|MUC4\|MUC12\|MUC16\|MUC17\|MUC15\|MUC20 | -5.69 |
| GO Cellular Components | dynein complex | 6 | 2.47 | DNAH17\|DNAH1\|DISC1\|DYNC2H1\|DNHD1\|DYNLT5 | -5.33 |
| GO Cellular Components | axonemal dynein complex | 3 | 1.23 | DNAH17\|DNAH1\|DNHD1 | -5.33 |
| GO Cellular Components | 9+2 motile cilium | 7 | 2.88 | DNAH17\|DNAH1\|DYNC2H1\|DNHD1\|TEKT5\|TTLL8\|CFAP47 | -5.33 |
| GO Cellular Components | microtubule associated complex | 6 | 2.47 | DNAH17\|DNAH1\|DISC1\|DYNC2H1\|DNHD1\|DYNLT5 | -5.33 |
| GO Cellular Components | motile cilium | 8 | 3.29 | DNAH17\|DNAH1\|DYNC2H1\|DNHD1\|TEKT5\|TTLL8\|SLC9C1\|CFAP47 | -5.33 |
| GO Cellular Components | sperm flagellum | 6 | 2.47 | DNAH17\|DNAH1\|DNHD1\|TEKT5\|TTLL8\|CFAP47 | -5.33 |
| GO Cellular Components | axoneme | 6 | 2.47 | DNAH17\|IFT140\|DNAH1\|DYNC2H1\|DNHD1\|TTLL8 | -5.33 |
| GO Cellular Components | ciliary plasm | 6 | 2.47 | DNAH17\|IFT140\|DNAH1\|DYNC2H1\|DNHD1\|TTLL8 | -5.33 |
| GO Cellular Components | cilium | 14 | 5.76 | DNAH17\|IFT140\|CEP170\|SDCCAG8\|DNAH1\|DISC1\|DYNC2H1\|LRRC56\|DNHD1\|TEKT5\|TTLL8\|FLCN\|SLC9C1\|CFAP47 | -5.33 |
| GO Cellular Components | plasma membrane bounded cell projection cytoplasm | 7 | 2.88 | CHRNA2\|DNAH17\|IFT140\|DNAH1\|DYNC2H1\|DNHD1\|TTLL8 | -5.33 |
| GO Cellular Components | centrosome | 17 | 7.00 | FANCE\|LIMK2\|PDE4DIP\|IFT140\|CEP170\|TACC3\|SDCCAG8\|ZNF365\|DCAF13\|DISC1\|ITSN2\|NCKAP5L\|CEP85\|HASPIN\|CEP112\|FLCN\|CROCC2 | -4.00 |
| GO Cellular Components | centriole | 5 | 2.06 | IFT140\|CEP170\|SDCCAG8\|CEP85\|CROCC2 | -4.00 |
| GO Cellular Components | extracellular matrix | 12 | 4.94 | MMP15\|MUC4\|SERPINB8\|SPARCL1\|FCGBP\|NID2\|SSPOP\|SNED1\|TNN\|MUC17\|COL22A1\|ADAMTS16 | -3.11 |
| GO Cellular Components | external encapsulating structure | 12 | 4.94 | MMP15\|MUC4\|SERPINB8\|SPARCL1\|FCGBP\|NID2\|SSPOP\|SNED1\|TNN\|MUC17\|COL22A1\|ADAMTS16 | -3.11 |
| GO Cellular Components | spindle | 9 | 3.70 | SLC25A5\|CDC6\|LIMK2\|CEP170\|TACC3\|CEP85\|HASPIN\|SPATA17\|FLCN | -2.81 |
| GO Cellular Components | clathrin-coated endocytic vesicle | 4 | 1.65 | KIAA0319\|TGOLN2\|SFTPA1\|SFTPA2 | -2.48 |
| GO Cellular Components | basolateral plasma membrane | 7 | 2.88 | AQP7\|P2RY4\|SLC4A2\|SLC6A12\|SLC2A9\|SLCO5A1\|MUC20 | -2.33 |
| GO Cellular Components | basal plasma membrane | 7 | 2.88 | AQP7\|P2RY4\|SLC4A2\|SLC6A12\|SLC2A9\|SLCO5A1\|MUC20 | -2.33 |
| GO Molecular Functions | extracellular matrix constituent, lubricant activity | 3 | 1.23 | MUC3A\|MUC4\|MUC17 | -5.69 |
| GO Molecular Functions | minus-end-directed microtubule motor activity | 4 | 1.65 | DNAH17\|DNAH1\|DYNC2H1\|DNHD1 | -5.33 |
| GO Molecular Functions | dynein intermediate chain binding | 5 | 2.06 | DNAH17\|DNAH1\|DYNC2H1\|DNHD1\|DYNLT5 | -5.33 |
| GO Molecular Functions | dynein light intermediate chain binding | 4 | 1.65 | DNAH17\|DNAH1\|DYNC2H1\|DNHD1 | -5.33 |
| GO Molecular Functions | microtubule motor activity | 4 | 1.65 | DNAH17\|DNAH1\|DYNC2H1\|DNHD1 | -5.33 |
| GO Molecular Functions | ATP-dependent activity | 12 | 4.94 | CDC6\|PMS2\|ACSM3\|DNAH17\|ABCA7\|SMCHD1\|DNAH1\|TOR2A\|DYNC2H1\|DNHD1\|ACSF3\|NLRP10 | -5.33 |
| GO Molecular Functions | olfactory receptor activity | 12 | 4.94 | OR6A2\|OR51A7\|OR4D11\|OR10A4\|OR10A2\|OR56B1\|OR52N5\|OR52N2\|OR8B3\|OR11G2\|OR51A2\|OR2T27 | -4.21 |
| GO Molecular Functions | calcium ion binding | 16 | 6.58 | ADGRE1\|FAT2\|PNLIPRP1\|TTN\|SPARCL1\|NID2\|SNED1\|EEF2K\|ITSN2\|CALML5\|PCDHGB2\|PCDHGA11\|PCDHGA7\|FKBP10\|NOTCH2NLA\|EFCAB8 | -3.73 |
| GO Molecular Functions | acid-thiol ligase activity | 3 | 1.23 | ACSM3\|SUCLG2\|ACSF3 | -2.71 |
| GO Molecular Functions | ligase activity | 6 | 2.47 | MTHFD1\|ACSM3\|SUCLG2\|EARS2\|TTLL8\|ACSF3 | -2.71 |
| GO Molecular Functions | ligase activity, forming carbon-sulfur bonds | 3 | 1.23 | ACSM3\|SUCLG2\|ACSF3 | -2.71 |
| GO Molecular Functions | secondary active transmembrane transporter activity | 7 | 2.88 | SLC25A5\|SLC4A2\|SLC6A12\|SLC2A9\|SLCO5A1\|SLC16A9\|SLC9C1 | -2.33 |
| GO Molecular Functions | oxidoreductase activity, acting on paired donors, with incorporation or reduction of molecular oxygen, reduced flavin or flavoprotein as one donor, and incorporation of one atom of oxygen | 3 | 1.23 | CYP3A7\|CYP39A1\|CYP4V2 | -2.30 |
| KEGG Pathway | Olfactory transduction | 13 | 5.35 | OR6A2\|CALML5\|OR51A7\|OR4D11\|OR10A4\|OR10A2\|OR56B1\|OR52N5\|OR52N2\|OR8B3\|OR11G2\|OR51A2\|OR2T27 | -4.21 |
| KEGG Pathway | Fanconi anemia pathway | 4 | 1.65 | FANCE\|PMS2\|TELO2\|POLN | -3.03 |
| KEGG Pathway | beta-Alanine metabolism | 3 | 1.23 | ALDH3A1\|ALDH1B1\|DPYD | -2.71 |
| KEGG Pathway | Pertussis | 4 | 1.65 | CALML5\|TIRAP\|SFTPA1\|SFTPA2 | -2.48 |

| Original | SNV | Gene | Chr | Position | Mut | Func | Mut_type | Shared |
| --- | --- | --- | --- | --- | --- | --- | --- | --- |
| WES | rs2224391 | FARS2 (Varview), LYRM4 (Varview) | 6p25.1 | 5260936 | A/C | exonic | missense | 2/9 |
| WES | rs5973488 | MAGEB16 | Xp21.1 | 35820795 | A/G | exonic | missense | 8/9 |
| WES | rs2303929 | SLC4A2 | 7q36.1 | 150761314 | G/A | exonic | missense | 2/9 |
| WES | rs1801159 | DPYD | 1p21.3 | 97981395 | T/C | exonic | missense | 1/7 |
| WES | rs1410961 | MAGEB16 | Xp21.1 | 35820425 | C/T | exonic | missense | 8/9 |
| WES | rs3779536 | FSCN3 | 7q32.1 | 127233977 | G/T | exonic | missense | 7/0 |
| WES | rs160632 | RIOK2 (Varview), LIX1-AS1 (Varview) | 5q15 | 96503523 | C/T | exonic | missense | 10/5 |
| WES | rs767620772 | LPIN2 | 18p11.31 | 2922117 | T/C | exonic | missense | 1/0 |
| WES | rs8191371 | GPI | 19q13.11 | 34868776 | T/C | exonic | missense | 1/0 |
| WES | rs117535913 | KCNJ1 | 11q24.3 | 128709618 | G/A | exonic | missense | 1/0 |
| WES | rs2304472 | PMM2 | 16p13.2 | 8895699 | A/T | exonic | missense | 1/0 |
| WES | rs766756088 | UCP2 | 11q13.4 | 73686110 | T/C | exonic | missense | 1/0 |
| WES | rs775044452 | IFT140 | 16p13.3 | 1570217 | G/A | exonic | missense | 1/0 |
| WES | rs111678678 | TET2 | 4q24 | 106158215 | C/T | exonic | missense | 1/0 |
| WES | rs150536710 | ZZEF1 | 17p13.2 | 3955389 | C/T | exonic | missense | 1/0 |
| WES | rs778093769 | FANCA | 16q24.3 | 89845355 | C/T | exonic | missense | 1/0 |
| WES | rs533349497 | FABP6 | 5q33.3 | 159665644 | G/A | exonic | missense | 1/0 |
| WES | rs201588284 | RREB1 | 6p24.3 | 7189437 | G/A | exonic | missense | 1/0 |
| WES | rs189506473 | TSHR | 14q31.1 | 81606063 | G/A | exonic | missense | 1/0 |
| WES | rs764256858 | COBLL1 | 2q24.3 | 165584496 | G/A | exonic | missense | 1/0 |
| WES | rs776254567 | LRBA | 4q31.3 | 151827481 | C/T | exonic | missense | 1/0 |
| WES | rs148962208 | IRS1 | 2q36.3 | 227660220 | G/A | exonic | missense | 0/1 |
| WES | rs6415788 | GLIS3 | 9p24.2 | 4118111 | G/T | exonic | missense | 0/10 |
| WES | rs202019027 | FASN | 17q25.3 | 80043146 | C/T | exonic | missense | 1/0 |
| WES | rs738409 | PNPLA3 | 22q13.31 | 44324727 | C/G | exonic | missense | 5/5 |
| WES | rs138772212 | PPARGC1B | 5q32 | 149219663 | G/A | exonic | missense | 1/0 |
| Literature | rs4402960 | IGF2BP2 | 3q27.2 | 185511687 | G/T | intron | intron variant |  |
| Literature | rs2971672 | GCK | 7p13 | 44205906 | A/C | intron | intron variant |  |
| Literature | rs8192678 | PPARGC1A | 4p15.2 | 23815662 | C/T | exonic | missense |  |
| Literature | rs2300587 | GCK | 7p13 | 44218794 | T/C | intron | intron variant |  |
| Literature | rs17032850 | NFKB1 | 4q24 | 103507703 | G/C | intron | intron variant |  |
| Literature | rs163184 | KCNQ1 | 11p15.5 | 2847069 | T/G | intron | intron variant |  |
| Literature | rs2021966 | ENPP1 | 6q23.2 | 132150439 | A/G | intron | intron variant |  |
| Literature | rs72696119 | NFKB1 (Varview), LOC105377621 (Varview) | 4q24 | 103422504 | C/G |  | NFKB1 : 2KB upstream variant |  |
|  |  |  |  |  |  |  | LOC105377621 : intron variant |  |
| Literature | rs11196218 | TCF7L2 | 10q25.2 | 114840494 | G/A | intron |  |  |
| Literature | rs822396 | ADIPOQ | 3q27.3 | 186566877 | G/A | intron |  |  |
| Literature | rs16856187 | G6PC2 | 2q24.3 | 169770386 | A/C |  | UPSTREAM GENE VARIANT SPC25; INTERGENIC VARIANT ABCB11 |  |
| Literature | rs13342232 | SLC16A11 (Varview), LOC124903909 (Varview) | 17p13.1 | 6945940 | A/G |  | Synonymous Variant,Upstream Variant |  |
| Literature | rs822393 | ADIPOQ | 3q27.3 | 186566326 | C/T | intron |  |  |
| Literature | rs16861194 | ADIPOQ | 3q27.3 | 186559425 | A/G |  | ADIPOQ : 2KB Upstream Variant |  |
| Literature | rs4737009 | ANK1 | 8p11.21 | 41630405 | G/A | exonic | Missense Variant |  |
| Literature | rs10882066 | IDE | 11q23.33 | 94224746 | A/G | intron |  |  |
| Literature | rs182052 | ADIPOQ | 3q27.3 | 186560782 | G/A | intron |  |  |
| Literature | rs10923931 | NOTCH2 | 1p12 | 120517959 | G/T | intron |  |  |
| Literature | rs6585205 | TCF7L2 | 10q25.2 | 114859164 | G/T | intron |  |  |
| Literature | rs7756992 | CDKAL1 | 6p22.3 | 20679709 | A/G | intron |  |  |
| Literature | rs12573128 | TCF7L2 | 10q25.2 | 114730797 | A/G | intron |  |  |
| Literature | rs10965250 | CDKN2B | 9q21.3 | 22133284 | G/A |  | INTERGENIC VARIANT DMRTA1 INTERGENIC VARIANT RP11-145E5.5 |  |
| Literature | rs225014 | DIO2 | 14q31.1 | 80669580 | T/C | exonic | Missense Variant |  |
| Literature | rs9368197 | CDKAL1 | 6p22.3 | 20533383 | G/T |  | CDKAL1 : 2KB Upstream Variant |  |
| Literature | rs6413453 | APOA2 | 1q23.3 | 161192316 | G/A |  | Intron Variant:SPLICE REGION VARIANT |  |
| Literature | rs937301 | GIP | 17q21.32 | 47046276 | A/G |  | GIP : 2KB Upstream Variant |  |
| Literature | rs1801282 | PPARG | 3p25.2 | 12393125 | C/G | exonic | Missense Variant |  |
| Literature | rs3792267 | CAPN10 | 2q37.3 | 241531174 | G/A | intron |  |  |
| Literature | rs1387153 | MTNR1B | 11q14.3 | 92673828 | C/T |  | SNRPGP16 :DOWNSTREAM GENE VARIANT;  MTNR1B :INTERGENIC VARIANT |  |
| Literature | rs7895340 | TCF7L2 | 10q25.2 | 114801525 | G/A | intron |  |  |
| Literature | rs1804764 | RARRES2 | 7q36.1 | 150036077 | G/A/T | exonic | Missense Variant |  |
| Literature | rs780094 | GCKR | 2p23.3 | 27741237 | T/C | intron |  |  |
| Literature | rs4845617 | IL6R (Varview), IL6R-AS1 (Varview) | 1q21.3 | 154377898 | G/A |  | IL6R-AS1 : Intron Variant IL6R : 5 Prime UTR Variant |  |
| Literature | rs10811661 | CDKN2A/B | 9p21.3 | 22134094 | T/C |  | DMRTA1 :INTERGENIC VARIANT; RP11-145E5.5: INTERGENIC VARIANT |  |
| Literature | rs1501299 | ADIPOQ (Varview), ADIPOQ-AS1 (Varview) | 3q27.3 | 186571 | C/T | intron |  |  |
| Literature | rs6517656 | BACE2 | 21q22.3 | 42583738 | G/A | intron |  |  |
| Literature | rs7747752 | CDKAL1 | 6p22.3 | 20725423 | G/C | intron |  |  |

Table S6. Information of 26 SNVs from WES and 37 SNVs from literature for genotyping .

Table S7. Shesis results of genotypes and alleles of the 16 candidate SNVs for validation between GDM sub-type cases and Controls.

|  | SNV | P | FDR | OR [95% CI] | Allele frequency |  | P | FDR | Genotype frequency |  |  | Call rate | HWE-P |
| --- | --- | --- | --- | --- | --- | --- | --- | --- | --- | --- | --- | --- | --- |
| only FBG high VS control | rs8192678 | 0.044 | 0.336 | 0.610 [0.377~0.984] | T | C | 0.083 | 0.254 | T/C | C/C | T/T | 0.995 | 0.917 |
|  | Case |  |  |  | 32(0.355) | 58(0.644) |  |  | 24(0.533) | 17(0.377) | 4(0.088) |  |  |
|  | Control |  |  |  | 170(0.474) | 188(0.525) |  |  | 90(0.502) | 49(0.273) | 40(0.223) |  |  |
|  | rs2971672 | 0.124 | 0.363 | 0.680 [0.424~1.091] | A | C | 0.301 | 0.471 | A/C | C/C | A/A | 0.991 | 0.727 |
|  | Case |  |  |  | 55(0.611) | 35(0.388) |  |  | 21(0.466) | 7(0.155) | 17(0.377) |  |  |
|  | Control |  |  |  | 184(0.516) | 172(0.483) |  |  | 84(0.471) | 44(0.247) | 50(0.28) |  |  |
| only OGTT high VS control | rs8192678 | 0.044 | 0.28 | 0.694 [0.490~0.982] | T | C | 0.118 | 0.506 | T/C | C/C | T/T | 0.989 | 0.993 |
|  | Case |  |  |  | 81(0.385) | 129(0.614) |  |  | 51(0.485) | 39(0.371) | 15(0.142) |  |  |
|  | Control |  |  |  | 170(0.474) | 188(0.525) |  |  | 90(0.502) | 49(0.273) | 40(0.223) |  |  |
|  | rs2971672 | 0.014 | 0.213 | 0.645 [0.455~0.913] | A | C | 0.038 | 0.506 | A/C | C/C | A/A | 0.986 | 0.943 |
|  | Case |  |  |  | 131(0.623) | 79(0.376) |  |  | 41(0.39) | 19(0.18) | 45(0.428) |  |  |
|  | Control |  |  |  | 184(0.516) | 172(0.483) |  |  | 84(0.471) | 44(0.247) | 50(0.28) |  |  |

Table S8. Shesis results of genotypes and alleles of the candidate SNVs for validation.

| SNP | Allele association | | | | | |  | Genotype association | | | | | | Call rate | HWE-P | |
| --- | --- | --- | --- | --- | --- | --- | --- | --- | --- | --- | --- | --- | --- | --- | --- | --- |
|  | Fisher's p | OR [95% CI] | FDR | Allele frequency | | |  | Fisher's p | FDR | Genotype frequency | | | |  | Pearson's p | Fisher's p |
| rs11196218 | 0.458 | 0.878 [0.636~1.213] | 0.812 | G | A |  |  | 0.541 | 0.827 | G/A | G/G | A/A |  | 0.992 | 0.943 | 0.966 |
| Case |  |  |  | 284(0.739) | 100(0.26) |  |  |  |  | 76(0.395) | 104(0.541) | 12(0.062) |  |  |  |  |
| Control |  |  |  | 257(0.713) | 103(0.286) |  |  |  |  | 69(0.383) | 94(0.522) | 17(0.094) |  |  |  |  |
| rs822396 | 0.714 | 0.901 [0.558~1.453] | 0.852 | A | G |  |  | 0.217 | 0.74 | A/A | G/A | G/G |  | 0.96 | 0.826 | 0.828 |
| Case |  |  |  | 335(0.9) | 37(0.099) |  |  |  |  | 153(0.822) | 29(0.155) | 4(0.021) |  |  |  |  |
| Control |  |  |  | 310(0.89) | 38(0.109) |  |  |  |  | 137(0.787) | 36(0.206) | 1(0.005) |  |  |  |  |
| rs8191371 | 0.424 | 0.674 [0.305~1.489] | 0.75 | T | C |  |  | 0.414 | 0.74 | T/T | C/T |  |  | 0.922 | 0.843 | 0.999 |
| Case |  |  |  | 347(0.969) | 11(0.03) |  |  |  |  | 168(0.938) | 11(0.061) |  |  |  |  |  |
| Control |  |  |  | 319(0.955) | 15(0.044) |  |  |  |  | 152(0.91) | 15(0.089) |  |  |  |  |  |
| rs117535913 | 0.754 | 1.39 [0.389~4.968] | 0.839 | G | A |  |  | 0.752 | 0.827 | G/G | G/A |  |  | 1 | 0.966 | 0.999 |
| Case |  |  |  | 384(0.984) | 6(0.015) |  |  |  |  | 189(0.969) | 6(0.03) |  |  |  |  |  |
| Control |  |  |  | 356(0.988) | 4(0.011) |  |  |  |  | 176(0.977) | 4(0.022) |  |  |  |  |  |
| rs148962208 | 0.507 | 1.869 [0.463~7.529] | 0.754 | G | A |  |  | 0.505 | 0.8 | G/G | G/A |  |  | 0.997 | 0.97 | 0.999 |
| Case |  |  |  | 382(0.984) | 6(0.015) |  |  |  |  | 188(0.969) | 6(0.03) |  |  |  |  |  |
| Control |  |  |  | 357(0.991) | 3(0.008) |  |  |  |  | 177(0.983) | 3(0.016) |  |  |  |  |  |
| rs163184 | 0.768 | 0.953 [0.714~1.271] | 0.852 | G | T |  |  | 0.908 | 0.954 | G/G | T/T | G/T |  | 0.989 | 0.951 | 0.966 |
| Case |  |  |  | 179(0.468) | 203(0.531) |  |  |  |  | 42(0.219) | 54(0.282) | 95(0.497) |  |  |  |  |
| Control |  |  |  | 173(0.48) | 187(0.519) |  |  |  |  | 43(0.238) | 50(0.277) | 87(0.483) |  |  |  |  |
| rs16856187 | 0.528 | 1.115 [0.818~1.52] | 0.829 | C | A |  |  | 0.647 | 0.827 | C/A | A/A | C/C |  | 0.994 | 0.015 | NA |
| Case |  |  |  | 126(0.326) | 260(0.673) |  |  |  |  | 100(0.518) | 80(0.414) | 13(0.067) |  |  |  |  |
| Control |  |  |  | 109(0.302) | 251(0.697) |  |  |  |  | 85(0.472) | 83(0.461) | 12(0.066) |  |  |  |  |
| rs13342232 | 0.395 | 1.258 [0.777~2.036] | 0.75 | A | G |  |  | 0.06 | 0.406 | A/A | G/G | G/A |  | 0.944 | 0.566 | NA |
| Case |  |  |  | 330(0.882) | 44(0.117) |  |  |  |  | 149(0.796) | 6(0.032) | 32(0.171) |  |  |  |  |
| Control |  |  |  | 302(0.904) | 32(0.095) |  |  |  |  | 135(0.808) | 0(0) | 32(0.191) |  |  |  |  |
| rs822393 | 0.46 | 0.892 [0.668~1.192] | 0.812 | C | T |  |  | 0.527 | 0.827 | C/C | T/T | C/T |  | 0.994 | 0.272 | NA |
| Case |  |  |  | 222(0.575) | 164(0.424) |  |  |  |  | 58(0.3) | 29(0.15) | 106(0.549) |  |  |  |  |
| Control |  |  |  | 197(0.547) | 163(0.452) |  |  |  |  | 52(0.288) | 35(0.194) | 93(0.516) |  |  |  |  |
| rs202019027 | NA | NA [NA~NA] | NA | C |  |  |  | NA | NA | C/C |  |  |  | 0.994 | 1 | NA |
| Case |  |  |  | 386(1) |  |  |  |  |  | 193(1) |  |  |  |  |  |  |
| Control |  |  |  | 360(1) |  |  |  |  |  | 180(1) |  |  |  |  |  |  |
| rs4402960 | 0.147 | 0.78 [0.558~1.091] | 0.75 | G | T |  |  | 0.036 | 0.375 | G/G | G/T | T/T |  | 0.976 | 0.36 | NA |
| Case |  |  |  | 292(0.772) | 86(0.227) |  |  |  |  | 111(0.587) | 70(0.37) | 8(0.042) |  |  |  |  |
| Control |  |  |  | 257(0.725) | 97(0.274) |  |  |  |  | 100(0.564) | 57(0.322) | 20(0.112) |  |  |  |  |
| rs2224391 | 0.664 | 0.926 [0.658~1.304] | 0.852 | C | A |  |  | 0.069 | 0.406 | C/A | A/A | C/C |  | 0.928 | 0.403 | NA |
| Case |  |  |  | 86(0.245) | 264(0.754) |  |  |  |  | 52(0.297) | 106(0.605) | 17(0.097) |  |  |  |  |
| Control |  |  |  | 90(0.26) | 256(0.739) |  |  |  |  | 70(0.404) | 93(0.537) | 10(0.057) |  |  |  |  |
| rs738409 | 0.364 | 1.155 [0.859~1.554] | 0.75 | G | C |  |  | 0.619 | 0.827 | G/C | C/C | G/G |  | 0.968 | 0.997 | 0.999 |
| Case |  |  |  | 158(0.424) | 214(0.575) |  |  |  |  | 92(0.494) | 61(0.327) | 33(0.177) |  |  |  |  |
| Control |  |  |  | 138(0.389) | 216(0.61) |  |  |  |  | 84(0.474) | 66(0.372) | 27(0.152) |  |  |  |  |
| rs16861194 | 0.621 | 1.118 [0.758~1.648] | 0.839 | A | G |  |  | 0.859 | 0.921 | A/A | G/A | G/G |  | 0.992 | 0.788 | NA |
| Case |  |  |  | 321(0.827) | 67(0.172) |  |  |  |  | 134(0.69) | 53(0.273) | 7(0.036) |  |  |  |  |
| Control |  |  |  | 300(0.842) | 56(0.157) |  |  |  |  | 127(0.713) | 46(0.258) | 5(0.028) |  |  |  |  |
| rs2304472 | 0.369 | 0.525 [0.152~1.809] | 0.75 | A | T |  |  | 0.366 | 0.74 | A/A | A/T |  |  | 0.997 | 0.96 | 0.999 |
| Case |  |  |  | 384(0.989) | 4(0.01) |  |  |  |  | 190(0.979) | 4(0.02) |  |  |  |  |  |
| Control |  |  |  | 353(0.98) | 7(0.019) |  |  |  |  | 173(0.961) | 7(0.038) |  |  |  |  |  |
| rs766756088 | NA | NA [NA~NA] | NA | T |  |  |  | NA | NA | T/T |  |  |  | 1 | 1 | NA |
| Case |  |  |  | 390(1) |  |  |  |  |  | 195(1) |  |  |  |  |  |  |
| Control |  |  |  | 360(1) |  |  |  |  |  | 180(1) |  |  |  |  |  |  |
| rs5973488 | 0.735 | 1.065 [0.764~1.485] | 0.852 | G | A |  |  | 0.515 | 0.827 | G/G | G/A | A/A |  | 0.989 | 0.911 | 0.963 |
| Case |  |  |  | 288(0.742) | 100(0.257) |  |  |  |  | 108(0.556) | 72(0.371) | 14(0.072) |  |  |  |  |
| Control |  |  |  | 267(0.754) | 87(0.245) |  |  |  |  | 98(0.553) | 71(0.401) | 8(0.045) |  |  |  |  |
| rs4737009 | 0.768 | 1.05 [0.786~1.402] | 0.852 | G | A |  |  | 0.942 | 0.955 | G/G | G/A | A/A |  | 0.984 | 0.961 | 0.985 |
| Case |  |  |  | 189(0.489) | 197(0.51) |  |  |  |  | 46(0.238) | 97(0.502) | 50(0.259) |  |  |  |  |
| Control |  |  |  | 168(0.477) | 184(0.522) |  |  |  |  | 39(0.221) | 90(0.511) | 47(0.267) |  |  |  |  |
| rs10882066 | 0.404 | 1.249 [0.78~2] | 0.75 | A | G |  |  | 0.514 | 0.76 | A/A | G/A | G/G |  | 0.984 | 0.478 | NA |
| Case |  |  |  | 339(0.882) | 45(0.117) |  |  |  |  | 149(0.776) | 41(0.213) | 2(0.01) |  |  |  |  |
| Control |  |  |  | 320(0.903) | 34(0.096) |  |  |  |  | 143(0.807) | 34(0.192) | 0(0) |  |  |  |  |
| rs775044452 | 0.481 | NA [NA~NA] | 0.75 | G | A |  |  | 0.481 | 0.74 | G/G | G/A |  |  | 0.997 | 0.998 | 0.999 |
| Case |  |  |  | 388(1) | 0(0) |  |  |  |  | 194(1) | 0(0) |  |  |  |  |  |
| Control |  |  |  | 359(0.997) | 1(0.002) |  |  |  |  | 179(0.994) | 1(0.005) |  |  |  |  |  |
| rs182052 | 0.547 | 0.909 [0.677~1.222] | 0.839 | G | A |  |  | 0.702 | 0.827 | G/A | G/G | A/A |  | 0.957 | 0.892 | NA |
| Case |  |  |  | 214(0.575) | 158(0.424) |  |  |  |  | 96(0.516) | 59(0.317) | 31(0.166) |  |  |  |  |
| Control |  |  |  | 191(0.552) | 155(0.447) |  |  |  |  | 85(0.491) | 53(0.306) | 35(0.202) |  |  |  |  |
| rs111678678 | 0.753 | 1.405 [0.393~5.021] | 0.839 | C | T |  |  | 0.752 | 0.827 | C/C | C/T |  |  | 0.994 | 0.965 | 0.999 |
| Case |  |  |  | 380(0.984) | 6(0.015) |  |  |  |  | 187(0.968) | 6(0.031) |  |  |  |  |  |
| Control |  |  |  | 356(0.988) | 4(0.011) |  |  |  |  | 176(0.977) | 4(0.022) |  |  |  |  |  |
| rs10923931 | 0.854 | 0.929 [0.447~1.93] | 0.934 | G | T |  |  | 0.842 | 0.827 | G/G | G/T | T/T |  | 0.994 | 0.867 | 0.999 |
| Case |  |  |  | 371(0.961) | 15(0.038) |  |  |  |  | 178(0.922) | 15(0.077) | 0(0) |  |  |  |  |
| Control |  |  |  | 345(0.958) | 15(0.041) |  |  |  |  | 166(0.922) | 13(0.072) | 1(0.005) |  |  |  |  |
| rs2021966 | 0.139 | 0.775 [0.561~1.069] | 0.691 | G | A |  |  | 0.278 | 0.74 | G/G | G/A | A/A |  | 0.898 | 0.791 | NA |
| Case |  |  |  | 242(0.699) | 104(0.3) |  |  |  |  | 87(0.502) | 68(0.393) | 18(0.104) |  |  |  |  |
| Control |  |  |  | 211(0.643) | 117(0.356) |  |  |  |  | 68(0.414) | 75(0.457) | 21(0.128) |  |  |  |  |
| rs2300587 | 0.04 | 1.464 [1.028~2.084] | 0.302 | C | T |  |  | 0.107 | 0.462 | C/T | T/T | C/C |  | 0.992 | 0.917 | 0.958 |
| Case |  |  |  | 96(0.248) | 290(0.751) |  |  |  |  | 70(0.362) | 110(0.569) | 13(0.067) |  |  |  |  |
| Control |  |  |  | 66(0.184) | 292(0.815) |  |  |  |  | 54(0.301) | 119(0.664) | 6(0.033) |  |  |  |  |
| rs6585205 | 0.94 | 1.016 [0.76~1.359] | 0.942 | T | G |  |  | 0.834 | 0.921 | T/T | G/T | G/G |  | 0.984 | 0.875 | NA |
| Case |  |  |  | 170(0.445) | 212(0.554) |  |  |  |  | 38(0.198) | 94(0.492) | 59(0.308) |  |  |  |  |
| Control |  |  |  | 157(0.441) | 199(0.558) |  |  |  |  | 32(0.179) | 93(0.522) | 53(0.297) |  |  |  |  |
| rs7756992 | 0.278 | 0.839 [0.619~1.137] | 0.75 | G | A |  |  | 0.455 | 0.827 | G/A | G/G | A/A |  | 0.89 | 0.576 | NA |
| Case |  |  |  | 187(0.546) | 155(0.453) |  |  |  |  | 83(0.485) | 52(0.304) | 36(0.21) |  |  |  |  |
| Control |  |  |  | 164(0.503) | 162(0.496) |  |  |  |  | 74(0.453) | 45(0.276) | 44(0.269) |  |  |  |  |
| rs12573128 | 0.742 | 1.056 [0.764~1.459] | 0.852 | A | G |  |  | 0.947 | 0.955 | A/A | G/A | G/G |  | 0.981 | 0.982 | 0.999 |
| Case |  |  |  | 109(0.282) | 277(0.717) |  |  |  |  | 16(0.082) | 77(0.398) | 100(0.518) |  |  |  |  |
| Control |  |  |  | 95(0.271) | 255(0.728) |  |  |  |  | 13(0.074) | 69(0.394) | 93(0.531) |  |  |  |  |
| rs150536710 | 0.999 | 1.239 [0.275~5.577] | 0.876 | C | T |  |  | 0.999 | 0.892 | C/T | C/C |  |  | 0.997 | 0.98 | 0.999 |
| Case |  |  |  | 384(0.989) | 4(0.01) |  |  |  |  | 4(0.02) | 190(0.979) |  |  |  |  |  |
| Control |  |  |  | 357(0.991) | 3(0.008) |  |  |  |  | 3(0.016) | 177(0.983) |  |  |  |  |  |
| rs10965250 | 0.435 | 0.876 [0.645~1.19] | 0.785 | G | A |  |  | 0.587 | 0.827 | G/A | A/A | G/G |  | 0.877 | 0.824 | NA |
| Case |  |  |  | 178(0.529) | 158(0.47) |  |  |  |  | 84(0.5) | 37(0.22) | 47(0.279) |  |  |  |  |
| Control |  |  |  | 160(0.496) | 162(0.503) |  |  |  |  | 86(0.534) | 38(0.236) | 37(0.229) |  |  |  |  |
| rs767620772 | 0.481 | NA [NA~NA] | 0.75 | T | C |  |  | 0.481 | 0.74 | T/T | C/T |  |  | 0.997 | 0.998 | 0.999 |
| Case |  |  |  | 388(1) | 0(0) |  |  |  |  | 194(1) | 0(0) |  |  |  |  |  |
| Control |  |  |  | 359(0.997) | 1(0.002) |  |  |  |  | 179(0.994) | 1(0.005) |  |  |  |  |  |
| rs778093769 | 0.375 | 3.72 [0.413~33.442] | 0.75 | C | T |  |  | 0.999 | 0.827 | C/C | C/T | T/T |  | 1 | 1.71e-13 | 1 |
| Case |  |  |  | 386(0.989) | 4(0.01) |  |  |  |  | 192(0.984) | 2(0.01) | 1(0.005) |  |  |  |  |
| Control |  |  |  | 359(0.997) | 1(0.002) |  |  |  |  | 179(0.994) | 1(0.005) | 0(0) |  |  |  |  |
| rs2303929 | 0.747 | 0.945 [0.688~1.296] | 0.852 | G | A |  |  | 0.676 | 0.827 | G/A | G/G | A/A |  | 0.992 | 0.248 | NA |
| Case |  |  |  | 277(0.713) | 111(0.286) |  |  |  |  | 83(0.427) | 97(0.5) | 14(0.072) |  |  |  |  |
| Control |  |  |  | 250(0.702) | 106(0.297) |  |  |  |  | 84(0.471) | 83(0.466) | 11(0.061) |  |  |  |  |
| rs225014 | 0.606 | 1.086 [0.813~1.449] | 0.839 | C | T |  |  | 0.79 | 0.892 | C/C | C/T | T/T |  | 0.986 | 0.408 | NA |
| Case |  |  |  | 183(0.476) | 201(0.523) |  |  |  |  | 48(0.25) | 87(0.453) | 57(0.296) |  |  |  |  |
| Control |  |  |  | 177(0.497) | 179(0.502) |  |  |  |  | 46(0.258) | 85(0.477) | 47(0.264) |  |  |  |  |
| rs533349497 | 0.625 | 2.782 [0.288~26.877] | 0.75 | G | A |  |  | 0.999 | 0.827 | G/G | G/A | A/A |  | 1 | 7.25e-21 | 1 |
| Case |  |  |  | 387(0.992) | 3(0.007) |  |  |  |  | 193(0.989) | 1(0.005) | 1(0.005) |  |  |  |  |
| Control |  |  |  | 359(0.997) | 1(0.002) |  |  |  |  | 179(0.994) | 1(0.005) | 0(0) |  |  |  |  |
| rs9368197 | 0.601 | 1.081 [0.806~1.45] | 0.839 | G | T |  |  | 0.749 | 0.884 | G/T | G/G | T/T |  | 0.976 | 0.823 | NA |
| Case |  |  |  | 166(0.436) | 214(0.563) |  |  |  |  | 94(0.494) | 36(0.189) | 60(0.315) |  |  |  |  |
| Control |  |  |  | 147(0.417) | 205(0.582) |  |  |  |  | 91(0.517) | 28(0.159) | 57(0.323) |  |  |  |  |
| rs6413453 | 0.999 | 0.994 [0.675~1.464] | 0.977 | G | A |  |  | 1 | 0.989 | G/A | G/G | A/A |  | 0.992 | 0.192 | NA |
| Case |  |  |  | 324(0.835) | 64(0.164) |  |  |  |  | 48(0.247) | 138(0.711) | 8(0.041) |  |  |  |  |
| Control |  |  |  | 297(0.834) | 59(0.165) |  |  |  |  | 45(0.252) | 126(0.707) | 7(0.039) |  |  |  |  |
| rs201588284 | 0.11 | NA [NA~NA] | 0.501 | G | A |  |  | 0.11 | 0.406 | G/G | G/A |  |  | 0.997 | 0.994 | 0.999 |
| Case |  |  |  | 388(1) | 0(0) |  |  |  |  | 194(1) | 0(0) |  |  |  |  |  |
| Control |  |  |  | 357(0.991) | 3(0.008) |  |  |  |  | 177(0.983) | 3(0.016) |  |  |  |  |  |
| rs937301 | 0.934 | 0.985 [0.713~1.359] | 0.942 | A | G |  |  | 0.081 | 0.427 | A/A | G/A | G/G |  | 0.994 | 0.563 | NA |
| Case |  |  |  | 281(0.727) | 105(0.272) |  |  |  |  | 100(0.518) | 81(0.419) | 12(0.062) |  |  |  |  |
| Control |  |  |  | 261(0.725) | 99(0.275) |  |  |  |  | 101(0.561) | 59(0.327) | 20(0.111) |  |  |  |  |
| rs189506473 | 0.5 | NA [NA~NA] | 0.75 | G | A |  |  | 0.499 | 0.681 | G/G | G/A |  |  | 1 | 0.996 | 1 |
| Case |  |  |  | 388(0.994) | 2(0.005) |  |  |  |  | 193(0.989) | 2(0.01) |  |  |  |  |  |
| Control |  |  |  | 360(1) | 0(0) |  |  |  |  | 180(1) | 0(0) |  |  |  |  |  |
| rs1801282 | 0.53 | 0.791 [0.426~1.466] | 0.812 | C | G |  |  | 0.62 | 0.827 | C/C | G/C | G/G |  | 0.989 | 0.973 | 0.999 |
| Case |  |  |  | 366(0.948) | 20(0.051) |  |  |  |  | 173(0.896) | 20(0.103) | 0(0) |  |  |  |  |
| Control |  |  |  | 333(0.935) | 23(0.064) |  |  |  |  | 156(0.876) | 21(0.117) | 1(0.005) |  |  |  |  |
| rs72696119 | 0.081 | 1.338 [0.965~1.856] | 0.503 | C | G |  |  | 0.09 | 0.431 | C/C | G/C | G/G |  | 0.802 | 0.195 | NA |
| Case |  |  |  | 175(0.56) | 137(0.439) |  |  |  |  | 50(0.32) | 75(0.48) | 31(0.198) |  |  |  |  |
| Control |  |  |  | 183(0.631) | 107(0.368) |  |  |  |  | 64(0.441) | 55(0.379) | 26(0.179) |  |  |  |  |
| rs2971672 | 0.007 | 0.665 [0.496~0.89] | 0.077 | C | A |  |  | 0.029 | 0.372 | C/A | A/A | C/C |  | 0.989 | 0.333 | NA |
| Case |  |  |  | 148(0.383) | 238(0.616) |  |  |  |  | 84(0.435) | 77(0.398) | 32(0.165) |  |  |  |  |
| Control |  |  |  | 172(0.483) | 184(0.516) |  |  |  |  | 84(0.471) | 50(0.28) | 44(0.247) |  |  |  |  |
| rs1801159 | 0.213 | 1.232 [0.888~1.709] | 0.75 | T | C |  |  | 0.068 | 0.406 | T/T | C/C | C/T |  | 0.989 | 0.75 | NA |
| Case |  |  |  | 276(0.715) | 110(0.284) |  |  |  |  | 104(0.538) | 21(0.108) | 68(0.352) |  |  |  |  |
| Control |  |  |  | 269(0.755) | 87(0.244) |  |  |  |  | 99(0.556) | 8(0.044) | 71(0.398) |  |  |  |  |
| rs3792267 | 0.999 | 0.973 [0.594~1.592] | 0.942 | G | A |  |  | 0.309 | 0.74 | G/G | G/A | A/A |  | 0.978 | 0.603 | NA |
| Case |  |  |  | 346(0.905) | 36(0.094) |  |  |  |  | 159(0.832) | 28(0.146) | 4(0.02) |  |  |  |  |
| Control |  |  |  | 318(0.903) | 34(0.096) |  |  |  |  | 143(0.812) | 32(0.181) | 1(0.005) |  |  |  |  |
| rs1410961 | 0.931 | 1.022 [0.73~1.43] | 0.942 | T | C |  |  | 0.397 | 0.805 | T/T | C/T | C/C |  | 0.968 | 0.974 | 0.992 |
| Case |  |  |  | 281(0.747) | 95(0.252) |  |  |  |  | 107(0.569) | 67(0.356) | 14(0.074) |  |  |  |  |
| Control |  |  |  | 263(0.751) | 87(0.248) |  |  |  |  | 96(0.548) | 71(0.405) | 8(0.045) |  |  |  |  |
| rs1387153 | 0.375 | 1.148 [0.859~1.535] | 0.75 | C | T |  |  | 0.646 | 0.827 | C/T | T/T | C/C |  | 0.978 | 0.334 | NA |
| Case |  |  |  | 193(0.505) | 189(0.494) |  |  |  |  | 87(0.455) | 51(0.267) | 53(0.277) |  |  |  |  |
| Control |  |  |  | 190(0.539) | 162(0.46) |  |  |  |  | 82(0.465) | 40(0.227) | 54(0.306) |  |  |  |  |
| rs7895340 | 0.999 | 1.061 [0.381~2.958] | 0.942 | G | A |  |  | 1 | 0.954 | G/G | G/A |  |  | 0.997 | 0.937 | 0.999 |
| Case |  |  |  | 380(0.979) | 8(0.02) |  |  |  |  | 186(0.958) | 8(0.041) |  |  |  |  |  |
| Control |  |  |  | 353(0.98) | 7(0.019) |  |  |  |  | 173(0.961) | 7(0.038) |  |  |  |  |  |
| rs764256858 | 0.482 | NA [NA~NA] | 0.75 | G | A |  |  | 0.482 | 0.74 | G/G | G/A |  |  | 0.994 | 0.998 | 0.999 |
| Case |  |  |  | 386(1) | 0(0) |  |  |  |  | 193(1) | 0(0) |  |  |  |  |  |
| Control |  |  |  | 359(0.997) | 1(0.002) |  |  |  |  | 179(0.994) | 1(0.005) |  |  |  |  |  |
| rs776254567 | 0.357 | 0.309 [0.032~2.985] | 0.75 | C | T |  |  | 0.356 | 0.74 | C/C | C/T |  |  | 0.994 | 0.991 | 0.999 |
| Case |  |  |  | 385(0.997) | 1(0.002) |  |  |  |  | 192(0.994) | 1(0.005) |  |  |  |  |  |
| Control |  |  |  | 357(0.991) | 3(0.008) |  |  |  |  | 177(0.983) | 3(0.016) |  |  |  |  |  |
| rs1804764 | NA | NA [NA~NA] | NA | G |  |  |  | NA | NA | G/G |  |  |  | 0.997 | 1 | NA |
| Case |  |  |  | 388(1) |  |  |  |  |  | 194(1) |  |  |  |  |  |  |
| Control |  |  |  | 360(1) |  |  |  |  |  | 180(1) |  |  |  |  |  |  |
| rs3779536 | 0.686 | 1.146 [0.674~1.948] | 0.839 | G | T |  |  | 0.393 | 0.825 | G/G | G/T | T/T |  | 0.997 | 0.615 | 0.878 |
| Case |  |  |  | 355(0.914) | 33(0.085) |  |  |  |  | 161(0.829) | 33(0.17) | 0(0) |  |  |  |  |
| Control |  |  |  | 333(0.925) | 27(0.075) |  |  |  |  | 154(0.855) | 25(0.138) | 1(0.005) |  |  |  |  |
| rs138772212 | 0.125 | NA [NA~NA] | 0.425 | G | A |  |  | 0.124 | 0.406 | G/G | G/A |  |  | 0.994 | 0.991 | 0.999 |
| Case |  |  |  | 384(0.989) | 4(0.01) |  |  |  |  | 190(0.979) | 4(0.02) |  |  |  |  |  |
| Control |  |  |  | 358(1) | 0(0) |  |  |  |  | 179(1) | 0(0) |  |  |  |  |  |
| rs780094 | 0.602 | 0.923 [0.689~1.236] | 0.839 | T | C |  |  | 0.852 | 0.921 | T/T | C/T | C/C |  | 0.965 | 0.988 | 0.999 |
| Case |  |  |  | 202(0.534) | 176(0.465) |  |  |  |  | 53(0.28) | 96(0.507) | 40(0.211) |  |  |  |  |
| Control |  |  |  | 178(0.514) | 168(0.485) |  |  |  |  | 46(0.265) | 86(0.497) | 41(0.236) |  |  |  |  |
| rs4845617 | 0.763 | NA [NA~NA] | 0.839 | A | G | C |  | 0.705 | 0.827 | A/A | G/A | G/C | G/G | 0.97 | 0.618 | NA |
| Case |  |  |  | 198(0.518) | 183(0.479) | 1(0.002) |  |  |  | 47(0.246) | 104(0.544) | 1(0.005) | 39(0.204) |  |  |  |
| Control |  |  |  | 174(0.502) | 172(0.497) | 0(0) |  |  |  | 43(0.248) | 88(0.508) | 0(0) | 42(0.242) |  |  |  |
| rs10811661 | 0.185 | 0.819 [0.613~1.093] | 0.75 | C | T |  |  | 0.266 | 0.74 | C/T | C/C | T/T |  | 0.989 | 0.868 | NA |
| Case |  |  |  | 171(0.447) | 211(0.552) |  |  |  |  | 93(0.486) | 39(0.204) | 59(0.308) |  |  |  |  |
| Control |  |  |  | 179(0.497) | 181(0.502) |  |  |  |  | 97(0.538) | 41(0.227) | 42(0.233) |  |  |  |  |
| rs1501299 | 0.736 | 0.935 [0.672~1.301] | 0.852 | G | T |  |  | 0.478 | 0.827 | G/T | T/T | G/G |  | 0.984 | 0.915 | NA |
| Case |  |  |  | 286(0.748) | 96(0.251) |  |  |  |  | 66(0.345) | 15(0.078) | 110(0.575) |  |  |  |  |
| Control |  |  |  | 262(0.735) | 94(0.264) |  |  |  |  | 72(0.404) | 11(0.061) | 95(0.533) |  |  |  |  |
| rs17032850 | 0.035 | 1.384 [1.029~1.862] | 0.302 | G | C |  |  | 0.111 | 0.462 | G/C | C/C | G/G |  | 0.989 | 0.26 | NA |
| Case |  |  |  | 221(0.569) | 167(0.43) |  |  |  |  | 89(0.458) | 39(0.201) | 66(0.34) |  |  |  |  |
| Control |  |  |  | 229(0.646) | 125(0.353) |  |  |  |  | 73(0.412) | 26(0.146) | 78(0.44) |  |  |  |  |
| rs8192678 | 0.005 | 0.658 [0.491~0.881] | 0.077 | C | T |  |  | 0.02 | 0.312 | C/T | C/C | T/T |  | 0.992 | 0.987 | 0.999 |
| Case |  |  |  | 242(0.626) | 144(0.373) |  |  |  |  | 90(0.466) | 76(0.393) | 27(0.139) |  |  |  |  |
| Control |  |  |  | 188(0.525) | 170(0.474) |  |  |  |  | 90(0.502) | 49(0.273) | 40(0.223) |  |  |  |  |
| rs160632 | 0.238 | 0.832 [0.623~1.111] | 0.75 | C | T |  |  | 0.394 | 0.805 | C/C | C/T | T/T |  | 0.989 | 0.67 | NA |
| Case |  |  |  | 170(0.44) | 216(0.559) |  |  |  |  | 34(0.176) | 102(0.528) | 57(0.295) |  |  |  |  |
| Control |  |  |  | 173(0.485) | 183(0.514) |  |  |  |  | 41(0.23) | 91(0.511) | 46(0.258) |  |  |  |  |
| rs6517656 | 0.436 | 0.75 [0.407~1.384] | 0.75 | G | A |  |  | 0.25 | 0.74 | G/G | G/A | A/A |  | 0.981 | 0.958 | 0.999 |
| Case |  |  |  | 364(0.947) | 20(0.052) |  |  |  |  | 173(0.901) | 18(0.093) | 1(0.005) |  |  |  |  |
| Control |  |  |  | 328(0.931) | 24(0.068) |  |  |  |  | 152(0.863) | 24(0.136) | 0(0) |  |  |  |  |
| rs6415788 | 0.999 | 1.481 [0.245~8.945] | 0.852 | T | G |  |  | 1 | 0.827 | T/T | G/T |  |  | 0.661 | 0.981 | 0.999 |
| Case |  |  |  | 247(0.988) | 3(0.012) |  |  |  |  | 122(0.976) | 3(0.024) |  |  |  |  |  |
| Control |  |  |  | 244(0.991) | 2(0.008) |  |  |  |  | 121(0.983) | 2(0.016) |  |  |  |  |  |
| rs7747752 | 0.506 | 1.114 [0.834~1.487] | 0.812 | C | G |  |  | 0.593 | 0.827 | C/C | G/C | G/G |  | 0.981 | 0.916 | NA |
| Case |  |  |  | 197(0.515) | 185(0.484) |  |  |  |  | 48(0.251) | 101(0.528) | 42(0.219) |  |  |  |  |
| Control |  |  |  | 173(0.488) | 181(0.511) |  |  |  |  | 43(0.242) | 87(0.491) | 47(0.265) |  |  |  |  |

Table S9 Loci chosen for haplotype analysis: rs11196218, rs822393, rs5973488, rs775044452, rs182052, rs6585205, rs7756992, rs10965250, rs767620772, rs778093769, rs72696119, rs1410961, rs764256858, rs10811661, rs17032850, rs7747752

| Haplotype | Case(freq) | Control(freq) | Chi2 | Fisher's p | Pearson's p | OR [95% CI] | Holm | SidakSS | SidakSD | FDR_BH | FDR_BY |
| --- | --- | --- | --- | --- | --- | --- | --- | --- | --- | --- | --- |
| GTGGAGAGTCGTGTCG | 9(0.033) | 7(0.028) | 0.118 | 0.804 | 0.73 | 1.191 [0.438~3.232] | 0.73 | 1 | 0.73 | 0.73 | 1 |
| GTGGAGGGTCCTGTGC | 7(0.026) | 15(0.06) | 3.698 | 0.081 | 0.054 | 0.42 [0.169~1.043] | 0.217 | 0.999 | 0.2 | 0.055 | 0.306 |
| ACGGGTGGTCCTGTGC | 23(0.086) | 11(0.044) | 3.493 | 0.078 | 0.061 | 1.988 [0.955~4.139] | 0.217 | 0.999 | 0.2 | 0.062 | 0.344 |
| GCGGGGAATCGTGCCG | 11(0.041) | 6(0.024) | 1.125 | 0.333 | 0.288 | 1.712 [0.626~4.679] | 0.577 | 1 | 0.494 | 0.29 | 1 |
| GCGGGGAATCCTGCGG | 5(0.018) | 15(0.06) | 6.001 | 0.021 | 0.014 | 0.298 [0.107~0.83] | 0.071 | 0.864 | 0.069 | 0.014 | 0.081 |

Table S10. Gene interaction results of the candidate SNVs for validation (P<0.05) .

| SNP set | | Nonmissing | Case Interaction | Control Interaction | diff | p | Holm | SidakSS | SidakSD | FDR_BH | FDR_BY |
| --- | --- | --- | --- | --- | --- | --- | --- | --- | --- | --- | --- |
| rs11196218 | rs150536710 | 372 | -0.002 | -0.01 | 0.008 | 0.032 | 1 | 1 | 1 | 0.933 | 1 |
| rs11196218 | rs4845617 | 361 | -0.019 | -0.006 | -0.012 | 0.018 | 1 | 0.999 | 0.999 | 0.933 | 1 |
| rs822396 | rs738409 | 351 | -0.014 | -0.032 | 0.018 | 0.023 | 1 | 1 | 1 | 0.933 | 1 |
| rs822396 | rs1801282 | 357 | -0.003 | -0.031 | 0.028 | 0.023 | 1 | 1 | 1 | 0.933 | 1 |
| rs8191371 | rs201588284 | 346 | 0 | -0.001 | 0.001 | 0.035 | 1 | 1 | 1 | 0.933 | 1 |
| rs8191371 | rs2971672 | 343 | -0.002 | -0.035 | 0.033 | 0.006 | 1 | 0.999 | 0.999 | 0.933 | 1 |
| rs117535913 | rs4402960 | 366 | -0.003 | -0.013 | 0.009 | 0.002 | 1 | 0.987 | 0.987 | 0.933 | 1 |
| rs117535913 | rs138772212 | 373 | -5.44e-04 | 0 | -5.44e-04 | 0.048 | 1 | 1 | 1 | 0.933 | 1 |
| rs148962208 | rs4402960 | 366 | -0.002 | -0.009 | 0.007 | 0.027 | 1 | 1 | 1 | 0.933 | 1 |
| rs148962208 | rs138772212 | 372 | -5.50e-04 | 0 | -5.50e-04 | 0.045 | 1 | 1 | 1 | 0.933 | 1 |
| rs163184 | rs201588284 | 371 | 0 | -0.004 | 0.004 | 0.041 | 1 | 1 | 1 | 0.933 | 1 |
| rs16856187 | rs3792267 | 366 | -0.023 | -0.005 | -0.017 | 0.024 | 1 | 1 | 1 | 0.933 | 1 |
| rs16856187 | rs1410961 | 362 | -0.004 | -0.028 | 0.023 | 0.037 | 1 | 1 | 1 | 0.933 | 1 |
| rs16856187 | rs8192678 | 371 | -0.011 | -0.003 | -0.007 | 0.037 | 1 | 1 | 1 | 0.933 | 1 |
| rs13342232 | rs7756992 | 323 | -0.012 | -0.002 | -0.009 | 0.036 | 1 | 1 | 1 | 0.933 | 1 |
| rs13342232 | rs225014 | 349 | -0.012 | -1.20e-05 | -0.012 | 0.007 | 1 | 0.999 | 0.999 | 0.933 | 1 |
| rs13342232 | rs1387153 | 348 | -0.034 | -0.001 | -0.033 | 0.011 | 1 | 0.999 | 0.999 | 0.933 | 1 |
| rs13342232 | rs776254567 | 352 | -0.009 | -0.003 | -0.005 | 0.006 | 1 | 0.999 | 0.999 | 0.933 | 1 |
| rs13342232 | rs160632 | 351 | -0.018 | -4.93e-05 | -0.018 | 0.017 | 1 | 0.999 | 0.999 | 0.933 | 1 |
| rs822393 | rs1501299 | 368 | -0.088 | -0.02 | -0.068 | 0.003 | 1 | 0.999 | 0.999 | 0.933 | 1 |
| rs4402960 | rs9368197 | 359 | -0.011 | -0.002 | -0.008 | 0.021 | 1 | 1 | 1 | 0.933 | 1 |
| rs4402960 | rs4845617 | 355 | -0.022 | -0.005 | -0.017 | 0.022 | 1 | 1 | 1 | 0.933 | 1 |
| rs4402960 | rs1501299 | 360 | -0.018 | -0.005 | -0.012 | 0.019 | 1 | 1 | 1 | 0.933 | 1 |
| rs2224391 | rs10882066 | 344 | -0.021 | -0.001 | -0.02 | 0.035 | 1 | 1 | 1 | 0.933 | 1 |
| rs2224391 | rs1387153 | 342 | -0.02 | -0.003 | -0.016 | 0.045 | 1 | 1 | 1 | 0.933 | 1 |
| rs738409 | rs2304472 | 363 | -0.002 | -0.019 | 0.016 | 0.045 | 1 | 1 | 1 | 0.933 | 1 |
| rs738409 | rs2300587 | 361 | -7.88e-04 | -0.012 | 0.012 | 0.038 | 1 | 1 | 1 | 0.933 | 1 |
| rs738409 | rs7756992 | 326 | -0.012 | -0.002 | -0.009 | 0.04 | 1 | 1 | 1 | 0.933 | 1 |
| rs16861194 | rs10923931 | 370 | -0.005 | -0.01 | 0.005 | 0.047 | 1 | 1 | 1 | 0.933 | 1 |
| rs16861194 | rs7895340 | 371 | -0.002 | -0.013 | 0.011 | 0.024 | 1 | 1 | 1 | 0.933 | 1 |
| rs16861194 | rs138772212 | 370 | -0.007 | 0 | -0.007 | 0.005 | 1 | 0.999 | 0.999 | 0.933 | 1 |
| rs5973488 | rs6413453 | 368 | -0.001 | -0.046 | 0.045 | 0.005 | 1 | 0.999 | 0.999 | 0.933 | 1 |
| rs5973488 | rs3779536 | 370 | -0.003 | -0.023 | 0.02 | 0.035 | 1 | 1 | 1 | 0.933 | 1 |
| rs10882066 | rs10965250 | 328 | -0.029 | -0.002 | -0.026 | 0.029 | 1 | 1 | 1 | 0.933 | 1 |
| rs10882066 | rs2303929 | 366 | -0.015 | -0.001 | -0.013 | 0.046 | 1 | 1 | 1 | 0.933 | 1 |
| rs10882066 | rs776254567 | 368 | -0.001 | -0.028 | 0.027 | 0.045 | 1 | 1 | 1 | 0.933 | 1 |
| rs10882066 | rs6415788 | 245 | -0.027 | -0.004 | -0.023 | 0.043 | 1 | 1 | 1 | 0.933 | 1 |
| rs182052 | rs3779536 | 359 | -0.008 | -0.037 | 0.028 | 0.012 | 1 | 0.999 | 0.999 | 0.933 | 1 |
| rs182052 | rs1501299 | 356 | -0.091 | -0.024 | -0.066 | 0.027 | 1 | 1 | 1 | 0.933 | 1 |
| rs111678678 | rs2303929 | 370 | -0.026 | -0.001 | -0.024 | 0.018 | 1 | 0.999 | 0.999 | 0.933 | 1 |
| rs111678678 | rs138772212 | 372 | -6.62e-04 | 0 | -6.62e-04 | 0.026 | 1 | 1 | 1 | 0.933 | 1 |
| rs10923931 | rs72696119 | 301 | -0.006 | -0.021 | 0.014 | 0.02 | 1 | 1 | 1 | 0.933 | 1 |
| rs10923931 | rs7895340 | 373 | -0.003 | -0.008 | 0.005 | 0.039 | 1 | 1 | 1 | 0.933 | 1 |
| rs10923931 | rs4845617 | 362 | -0.005 | -0.036 | 0.03 | 0.01 | 1 | 0.999 | 0.999 | 0.933 | 1 |
| rs2021966 | rs780094 | 326 | -0.006 | -0.03 | 0.023 | 0.016 | 1 | 0.999 | 0.999 | 0.933 | 1 |
| rs2021966 | rs6517656 | 330 | -0.004 | -0.015 | 0.01 | 0.019 | 1 | 1 | 0.999 | 0.933 | 1 |
| rs2300587 | rs150536710 | 371 | -0.016 | -0.006 | -0.009 | 0.022 | 1 | 1 | 1 | 0.933 | 1 |
| rs2300587 | rs3779536 | 371 | -1.85e-04 | -0.017 | 0.016 | 0.035 | 1 | 1 | 1 | 0.933 | 1 |
| rs6585205 | rs12573128 | 362 | -0.013 | -0.054 | 0.041 | 0.049 | 1 | 1 | 1 | 0.933 | 1 |
| rs6585205 | rs10965250 | 325 | -0.001 | -0.012 | 0.011 | 0.049 | 1 | 1 | 1 | 0.933 | 1 |
| rs6585205 | rs1801159 | 366 | -0.004 | -0.021 | 0.016 | 0.018 | 1 | 0.999 | 0.999 | 0.933 | 1 |
| rs7756992 | rs201588284 | 334 | 0 | -0.005 | 0.005 | 0.033 | 1 | 1 | 1 | 0.933 | 1 |
| rs7756992 | rs1387153 | 333 | -0.023 | -0.006 | -0.017 | 0.023 | 1 | 1 | 1 | 0.933 | 1 |
| rs7756992 | rs7895340 | 334 | -4.33e-04 | -0.027 | 0.026 | 0.016 | 1 | 0.999 | 0.999 | 0.933 | 1 |
| rs12573128 | rs1801159 | 366 | -0.018 | -0.006 | -0.011 | 0.039 | 1 | 1 | 1 | 0.933 | 1 |
| rs12573128 | rs17032850 | 366 | -0.007 | -0.035 | 0.027 | 0.026 | 1 | 1 | 1 | 0.933 | 1 |
| rs767620772 | rs201588284 | 374 | 0 | -9.36e-05 | 9.36e-05 | 0.047 | 1 | 1 | 1 | 0.933 | 1 |
| rs2303929 | rs776254567 | 370 | -0.004 | -0.002 | -0.002 | 0.045 | 1 | 1 | 1 | 0.933 | 1 |
| rs9368197 | rs138772212 | 364 | -0.015 | 0 | -0.015 | 0.026 | 1 | 1 | 1 | 0.933 | 1 |
| rs6413453 | rs1410961 | 362 | -0.001 | -0.047 | 0.046 | 0.003 | 1 | 0.999 | 0.999 | 0.933 | 1 |
| rs201588284 | rs776254567 | 373 | 0 | -2.82e-04 | 2.82e-04 | 0.04 | 1 | 1 | 1 | 0.933 | 1 |
| rs201588284 | rs6517656 | 367 | 0 | -0.002 | 0.002 | 0.049 | 1 | 1 | 1 | 0.933 | 1 |
| rs1801282 | rs6517656 | 366 | -0.001 | -0.015 | 0.014 | 0.048 | 1 | 1 | 1 | 0.933 | 1 |
| rs72696119 | rs1801159 | 301 | -0.03 | -0.001 | -0.028 | 0.024 | 1 | 1 | 1 | 0.933 | 1 |
| rs72696119 | rs138772212 | 301 | -0.007 | 0 | -0.007 | 0.048 | 1 | 1 | 1 | 0.933 | 1 |
| rs1410961 | rs3779536 | 363 | -0.004 | -0.019 | 0.015 | 0.019 | 1 | 1 | 1 | 0.933 | 1 |
| rs7895340 | rs7747752 | 367 | -1.56e-04 | -0.028 | 0.028 | 0.007 | 1 | 0.999 | 0.999 | 0.933 | 1 |
| rs10811661 | rs1501299 | 367 | -0.003 | -0.02 | 0.016 | 0.038 | 1 | 1 | 1 | 0.933 | 1 |
| rs6517656 | rs7747752 | 367 | -0.037 | -0.002 | -0.034 | 0.023 | 1 | 1 | 1 | 0.933 | 1 |

Table S11. Maternal characteristics between cases and controls among the sub-population of 155 pregnant women

|  | Cases（n=72） | | Controls（n=83） | | Crude OR (95% CI) | Z | P |
| --- | --- | --- | --- | --- | --- | --- | --- |
| Age (Years, Mean±SD) | 30.81±4.99 | | 29.47±3.96 | | 1.07 (1.00-1.15) | 1.84 | 0.065 |
| TG（n(%)） |  |  |  |  |  |  |  |
| Low: <1.7 mmol/L | 45 (62.50) | | 70 (84.34) | | 1.00 | - | - |
| High: ≥1.7 mmol/L | 27 (37.50) | | 13 15.66) | | 3.23 (1.21-6.93) | 3.01 | 0.003 |
| TC（n, %） |  |  |  |  |  |  |  |
| <5.2 mmol/L | 56 (77.78) | | 74 (89.16) | | 1.00 | - | - |
| ≥5.2 mmol/L | 16 (22.22) | | 9 (10.84) | | 2.35( 0.96-5.72) | 1.88 | 0.060 |
| HDL（n, %） |  |  |  |  |  |  |  |
| ≤1.94 mmol/L | 68 (94.44) | | 77 (92.77) | | 1.32 (0.36-4.91) | 0.42 | 0.674 |
| >1.94 mmol/L | 4 (5.56) | | 6 (7.23) | | 1.00 | - | - |
| LDL（n, %） |  |  |  |  |  |  |  |
| <2.6 mmol/L | 18 (25.00) | | 21 (25.3) | | 1.00 | - | - |
| ≥2.6mmol/L | 54 (75.00) | | 62 (74.7) | | 1.02 (0.49-2.11) | 0.04 | 0.966 |
| TSH (mIU/L) (Median) | 0.86 | | 1.09 | | - | - | - |
| TSH (mIU/L) (Range) | 0-3.19 | | 0-6.80 | | - | - | - |
| SQRT (TSH (mIU/L))(Mean±SD) | 0.91±0.35 | | 1.06±0.46 | | 0.42(0.18-0.95) | -2.09 | 0.037 |
| SQRT (TSH (mIU/L))(Range) | 0-1.79 | | 0-2.61 | | - | - | - |
| BPA (ng/ml) (Median) ^a^ | 5.76 | | 4.63 | | - | - | - |
| BPA (ng/ml) (Range) ^a^ | 0.07-35.48 | | 0.05- 19.81 | | - | - | - |
| SQRT ( BPA (ng/ml))(Mean±SD) ^a^ | 2.54±0.97 | | 2.14±0.70 | | 1.84(1.20-2.81) | 2.80 | 0.005 |
| SQRT ( BPA (ng/ml))(Range) ^a^ | 0.27-5.96 | | 0.23-4.45 | | - | - | - |
| TSH |  | |  | |  |  |  |
| <0.91 mIU/L | 43 (59.72) | | 35 (42.17) | | 1.00 | - | - |
| >=0.91 mIU/L | 29 (40.28) | | 48 (57.83) | | 1.92 (1.01-3.64) | 0.65 | 0.046 |
| BPA |  | |  | |  |  |  |
| <4.9 ng/ml | 30 (41.67) | | 48 (57.83) | | 1.00 | - | - |
| >=4.9 ng/ml | 42 (58.33) | | 35 (42.17) | | 0.49 (0.26-0.93) | -0.71 | 0.030 |
| BMI |  | |  | |  |  |  |
| Normal (<18.5 or >=24.0 kg/m2) | 60 (83.33 ) | | 74 (89.16) | | 1.00 | - | - |
| Abnormal (18.5-23.9 kg/m2) | 12 (16.67) | | 9 (10.84) | | 1.64 (0.65-4.16) | 1.05 | 0.294 |
| Gravidity |  | |  | |  |  |  |
| <2 | 22 (30.56) | | 35 (42.17) | | 1.00 | - | - |
| >=2 | 50 (69.44) | | 48 (57.83) | | 1.66 (0.85-3.22) | 1.49 | 0.136 |
| Parity |  | |  | |  |  |  |
| <1 | 27 (37.50) | | 56 (67.47) | | 1.00 | - | - |
| >=1 | 45 (62.50) | | 27 (32.53) | | 3.46 (1.78-6.70) | 3.67 | 0.000 |

^a^ BPA concentrations were corrected by specific gravity

Table S12 Summary of logistic regression results (Odds Ratios and P-values)

|  | Reduced model | Full model 1 | Full model 2 | Full model 3 |
| --- | --- | --- | --- | --- |
| **Main effect** |  |  |  |  |
| rs8192678 |  |  |  |  |
| CT vs CC | 0.388* (0.034) | 0.515（0.622) | 0.201（0.183) | 0.346*（0.036) |
| TT vs CC | 0.294* (0.023) | 0.244（0.489) | 0.296（0.555) | 0.441（0.195) |
| SQRT (BPA) | 2.295** (0.002) | 2.423*（0.043) | 2.307**（0.002) | 2.299**（0.002) |
| SQRT (TSH) | 0.386* (0.048) | 0.384*（0.047) | 0.247（0.150) | 0.390（0.053) |
| TG (>=1.7 mmol/L vs <1.7 mmol/L) | 3.071* (0.023) | 3.128*（0.022) | 3.127*（0.022) | 3.575（0.181) |
| Maternal BMI | 1.352 (0.530) | 1.346（0.601) | 1.314（0.635) | 1.187（0.766) |
| Maternal gravidity | 0.235（0.030) | 0.227*（0.030) | 0.234*（0.029) | 0.233*（0.030) |
| Maternal parity | 8.919**（0.001) | 9.157**（0.001) | 8.576**（0.001) | 9.088**（0.001) |
| Maternal age | 1.008（0.864) | 1.005（0.918) | 1.010（0.839) | 1.011（0.815) |
| Interaction effect |  |  |  | 0.267（0.437) |
| rs8192678(CT)#SQRT (BPA) | - | 0.883（0.825) | - | - |
| rs8192678(TT)#SQRT (BPA) | - | 1.094（0.92) | - | - |
| rs8192678(CT)#SQRT (TSH) | - | - | 1.938（0.555) | - |
| rs8192678(TT)#SQRT (TSH) | - | - | 0.948（0.980) | - |
| rs8192678(CT)#TG (>=1.7 vs <1.7 mmol/L) | - | - | - | 1.396（0.768) |
| rs8192678(TT)#TG (>=1.7 vs <1.7 mmol/L) | - | - | - | 0.223（0.281) |
| Constant | 0.299（0.471) | 0.295（0.508) | 0.455（0.668) |  |
| **Model statistics** |  |  |  |  |
| Sample size | 152 | 152 | 152 | 152 |
| Log likelihood | -82.437 | -82.395 | -82.216 | -81.228 |
| Pseudo R² | 0.214 | 0.214 | 0.216 | 0.226 |
| LR χ²(df) | 44.89(9)*** | 44.98(11)*** | 45.34(11)** | 47.31(8)*** |
| Prob > χ² | 0.00000 | 0.00000 | 0.00000 | 0.00000 |
| **Likelihood ratio test results** |  |  |  |  |
| LR χ² | ref | 0.08 | 0.44 | -0.94 |
| df | ref | 2 | 2 | 2 |
| P value | ref | 0.9588 | 0.8018 | 1.0000 |

Table S13 Analysis of average marginal effects between PPARGC1A rs8192678, TG and GDM

|  | dy/dx with 95% CI ^a^ | Std. Err. | z | P>z |
| --- | --- | --- | --- | --- |
| **Model 1** |  |  |  |  |
| rs8192678 |  |  |  |  |
| CT vs CC | -0.175 (-0.330~-0.021) | 0.079 | -2.23 | 0.026 |
| TT vs CC | -0.224 (-0.419~-0.029) | 0.099 | -2.26 | 0.024 |
| TG: >=1.7 vs <1.7 mmol/L | 0.214 (0.036-0.391) | 0.091 | 2.36 | 0.018 |
| **Model 2** |  |  |  |  |
| rs8192678 (C>T): CT/TT vs CC | -0.190 (-0.333~-0.046) | 0.073 | -2.6 | 0.009 |
| TG: >=1.7 vs <1.7 mmol/L | 0.217 (0.040~0.394) | 0.091 | 2.40 | 0.017 |
| **Model 1** |  |  |  |  |
| rs8192678 (C>T): TT vs CC/CT | -0.130 (-0.308~0.050) | 0.091 | -1.41 | 0.159 |
| TG: >=1.7 vs <1.7 mmol/L | 0.201 (0.019~0.383) | 0.093 | 2.16 | 0.031 |

Abbreviations: GDM, gestational diabetes mellitus; aOR, adjusted odds ratio; CI, confidence interval; BPA, bisphenol A; TG, triglyceride; TSH, thyroid-stimulating hormone..

^a^ Estimations were adjusted for maternal age, body mass index, gravidity, parity, BPA and TSH levels.

Table S14 Analysis of average marginal effects between PPARGC1A rs8192678 and GDM at different natural sqrt-transformed BPA and TSH levels

|  | rs8192678 CT vs CC | |  | rs8192678 TT vs CC | |  | rs8192678 TT+CT vs CC | |  | rs8192678 TT vs CC+CT | |
| --- | --- | --- | --- | --- | --- | --- | --- | --- | --- | --- | --- |
|  | dy/dx with 95% CI ^a^ | P |  | dy/dx with 95% CI ^a^ | P |  | dy/dx with 95% CI ^a^ | P |  | dy/dx with 95% CI ^a^ | P |
| SQRT(BPA) |  |  |  |  |  |  |  |  |  |  |  |
| 0 | -0.116(-0.24~0.007) | 0.065 |  | -0.14(-0.286~0.005) | 0.058 |  | -0.122(-0.244~0) | 0.051 |  | -0.072(-0.179~0.034) | 0.184 |
| 1 | -0.16(-0.307~-0.014) | 0.032 |  | -0.198(-0.369~-0.027) | 0.023 |  | -0.171(-0.31~-0.032) | 0.016 |  | -0.106(-0.248~0.037) | 0.145 |
| 2 | -0.185(-0.349~-0.021) | 0.027 |  | -0.235(-0.435~-0.035) | 0.022 |  | -0.2(-0.352~-0.048) | 0.010 |  | -0.132(-0.312~0.048) | 0.150 |
| 3 | -0.178(-0.333~-0.023) | 0.025 |  | -0.233(-0.443~-0.023) | 0.030 |  | -0.194(-0.337~-0.051) | 0.008 |  | -0.14(-0.339~0.06) | 0.171 |
| 4 | -0.143(-0.273~-0.013) | 0.031 |  | -0.193(-0.39~0.004) | 0.054 |  | -0.156(-0.2778~-0.034) | 0.012 |  | -0.125(-0.317~0.068) | 0.204 |
| 5 | -0.098(-0.207~0.011) | 0.077 |  | -0.137(-0.311~0.037) | 0.124 |  | -0.106(-0.214~0.001) | 0.052 |  | -0.095(-0.26~0.07) | 0.259 |
| 6 | -0.058(-0.147~0.031) | 0.199 |  | -0.083(-0.226~0.059) | 0.251 |  | -0.063(-0.155~0.029) | 0.179 |  | -0.063(-0.193~0.067) | 0.342 |
| SQRT(TSH) |  |  |  |  |  |  |  |  |  |  |  |
| 0.0 | -0.164(-0.309~-0.019) | 0.026 |  | -0.217(-0.420~-0.015) | 0.035 |  | -0.179(-0.312~-0.045) | 0.009 |  | -0.133(-0.329~0.061) | 0.180 |
| 0.5 | -0.178(-0.333~-0.022) | 0.025 |  | -0.231(-0.436~-0.025) | 0.027 |  | -0.193(-0.337~-0.049) | 0.008 |  | -0.136(-0.329~0.057) | 0.167 |
| 1.0 | -0.181(-0.341~-0.021) | 0.026 |  | -0.231(-0.432~-0.030) | 0.024 |  | -0.196(-0.344~-0.047) | 0.010 |  | -0.132(-0.315~0.050) | 0.157 |
| 1.5 | -0.174(-0.329~-0.019) | 0.027 |  | -0.219(-0.406~-0.031) | 0.022 |  | -0.187(-0.332~-0.042) | 0.011 |  | -0.122(-0.288~0.044) | 0.150 |
| 2.0 | -0.158(-0.303~-0.013) | 0.032 |  | -0.197(-0.369~-0.024) | 0.025 |  | -0.169(-0.307~-0.031) | 0.016 |  | -0.108(-0.255~0.039) | 0.152 |
| 2.5 | -0.137(-0.275~0.001) | 0.052 |  | -0.168(-0.333~-0.003) | 0.046 |  | -0.146(-0.281~-0.010) | 0.035 |  | -0.091(-0.223~0.040) | 0.173 |
| 3.0 | -0.113(-0.250~0.023) | 0.105 |  | -0.137(-0.303~0.027) | 0.103 |  | -0.119(-0.258~0.018) | 0.090 |  | -0.075(-0.195~0.045) | 0.223 |

Abbreviations: GDM, gestational diabetes mellitus; aOR, adjusted odds ratio; CI, confidence interval; SQRT (BPA), bisphenol A concentration was transformed by square root; TG, triglyceride; SQRT (TSH) , thyroid-stimulating hormone concentration was transformed by square root.

^a^ Estimations were adjusted for maternal age, body mass index, gravidity, parity, and TG.

Table S15 Analysis of average marginal effects between TG and GDM at different natural sqrt-transformed BPA and TSH levels

|  | Model 1 | |  | Model 2 | |  | Model 3 | |
| --- | --- | --- | --- | --- | --- | --- | --- | --- |
|  | dy/dx with 95% CI | P |  | dy/dx with 95% CI | P |  | dy/dx with 95% CI | P |
| SQRT(BPA) |  |  |  |  |  |  |  |  |
| 0 | 0.143(-0.021~0.308) | 0.087 |  | 0.143(-0.02~0.307) | 0.087 |  | 0.137(-0.031~0.307) | 0.110 |
| 1 | 0.198(0.013~0.383) | 0.036 |  | 0.2(0.015~0.384) | 0.034 |  | 0.188(-0.001~0.378) | 0.052 |
| 2 | 0.224(0.034~0.414) | 0.020 |  | 0.227(0.038~0.416) | 0.018 |  | 0.212(0.017~0.407) | 0.033 |
| 3 | 0.208(0.043~0.374) | 0.013 |  | 0.21(0.046~0.375) | 0.012 |  | 0.198(0.026~0.37) | 0.024 |
| 4 | 0.163(0.027~0.298) | 0.018 |  | 0.163(0.028~0.297) | 0.017 |  | 0.157(0.015~0.298) | 0.029 |
| 5 | 0.109(-0.006~0.226) | 0.065 |  | 0.108(-0.006~0.223) | 0.065 |  | 0.108(-0.012~0.23) | 0.079 |
| 6 | 0.064(-0.033~0.162) | 0.196 |  | 0.062(-0.033~0.158) | 0.199 |  | 0.066(-0.036~0.169) | 0.204 |
| SQRT(TSH) |  |  |  |  |  |  |  |  |
| 0.0 | 0.19(0.042~0.337) | 0.011 |  | 0.192(0.045~0.338) | 0.010 |  | 0.182(0.027~0.337) | 0.021 |
| 0.5 | 0.21(0.043~0.377) | 0.013 |  | 0.213(0.047~0.38) | 0.012 |  | 0.199(0.025~0.373) | 0.025 |
| 1.0 | 0.219(0.036~0.402) | 0.019 |  | 0.222(0.039~0.405) | 0.017 |  | 0.206(0.018~0.394) | 0.031 |
| 1.5 | 0.215(0.029~0.400) | 0.023 |  | 0.217(0.032~0.403) | 0.021 |  | 0.203(0.012~0.393) | 0.036 |
| 2.0 | 0.197(0.019~0.375) | 0.030 |  | 0.199(0.021~0.377) | 0.028 |  | 0.189(0.005~0.372) | 0.043 |
| 2.5 | 0.17(-0.001~0.342) | 0.052 |  | 0.171(0.000~0.343) | 0.051 |  | 0.166(-0.009~0.343) | 0.064 |
| 3.0 | 0.139(-0.03~0.309) | 0.108 |  | 0.139(-0.03~0.310) | 0.108 |  | 0.140(-0.035~0.315) | 0.117 |

Abbreviations: GDM, gestational diabetes mellitus; aOR, adjusted odds ratio; CI, confidence interval; SQRT (BPA), bisphenol A concentration was transformed by square root; TG, triglyceride; SQRT (TSH) , thyroid-stimulating hormone concentration was transformed by square root.

^a^ Estimations were adjusted for maternal age, body mass index, gravidity, parity and PPARGC1A rs8192678 .

Table S16 Validated marginal effect o rs8192678 at varying BPA and TSH levls ^a^

|  | rs8192678 CT vs CC | |  | rs8192678 TT vs CC | |  | rs8192678 TT+CT vs CC | |  | rs8192678 TT vs CC+CT | |
| --- | --- | --- | --- | --- | --- | --- | --- | --- | --- | --- | --- |
|  | dy/dx with 95% CI ^b^ | P |  | dy/dx with 95% CI ^b^ | P |  | dy/dx with 95% CI ^b^ | P |  | dy/dx with 95% CI ^b^ | P |
| SQRT(BPA) |  |  |  |  |  |  |  |  |  |  |  |
| 0 | -0.116(-0.259~0.027) | 0.111 |  | -0.140(-0.317~0.036) | 0.120 |  | -0.122(0.100~-0.268) | 0.100 |  | -0.072(-0.201~0.056) | 0.271 |
| 1 | -0.160(-0.318~-0.003) | 0.046 |  | -0.198(-0.405~0.009) | 0.061 |  | -0.171(0.035~-0.330) | 0.035 |  | -0.106(-0.278~0.067) | 0.230 |
| 2 | -0.185(-0.359~-0.012) | 0.037 |  | -0.235(-0.485~0.015) | 0.065 |  | -0.200(0.023~-0.373) | 0.023 |  | -0.132(-0.352~0.088) | 0.240 |
| 3 | -0.178(-0.345~-0.011) | 0.037 |  | -0.233(-0.501~0.035) | 0.088 |  | -0.194(0.022~-0.359) | 0.022 |  | -0.140(-0.385~0.106) | 0.266 |
| 4 | -0.143(-0.290~0.003) | 0.055 |  | -0.193(-0.447~0.060) | 0.134 |  | -0.156(0.037~-0.302) | 0.037 |  | -0.125(-0.361~0.112) | 0.302 |
| 5 | -0.098(-0.229~0.033) | 0.143 |  | -0.137(-0.359~0.086) | 0.229 |  | -0.106(0.116~-0.239) | 0.116 |  | -0.095(-0.298~0.108) | 0.360 |
| 6 | -0.058(-0.171~0.055) | 0.312 |  | -0.083(-0.265~0.098) | 0.369 |  | -0.063(0.283~-0.178) | 0.283 |  | -0.063(-0.226~0.099) | 0.446 |
| SQRT(TSH) |  |  |  |  |  |  |  |  |  |  |  |
| 0.0 | -0.165(-0.322~-0.007) | 0.041 |  | -0.218(-0.474~0.039) | 0.096 |  | -0.179(-0.333~-0.026) | 0.022 |  | -0.134(-0.373~0.106) | 0.274 |
| 0.5 | -0.178(-0.344~-0.012) | 0.036 |  | -0.231(-0.493~0.031) | 0.083 |  | -0.193(-0.359~-0.028) | 0.022 |  | -0.136(-0.375~0.102) | 0.263 |
| 1.0 | -0.181(-0.352~-0.011) | 0.037 |  | -0.232(-0.485~0.022) | 0.073 |  | -0.196(-0.367~-0.025) | 0.024 |  | -0.132(-0.357~0.093) | 0.250 |
| 1.5 | -0.174(-0.340~-0.009) | 0.039 |  | -0.219(-0.451~0.012) | 0.063 |  | -0.187(-0.353~-0.022) | 0.026 |  | -0.122(-0.324~0.080) | 0.236 |
| 2.0 | -0.159(-0.316~-0.002) | 0.048 |  | -0.197(-0.404~0.010) | 0.062 |  | -0.169 (-0.326~-0.013) | 0.033 |  | -0.108(-0.284~0.068) | 0.228 |
| 2.5 | -0.137(-0.292~0.017) | 0.081 |  | -0.169(-0.361~0.024) | 0.086 |  | -0.146(-0.299~0.007) | 0.061 |  | -0.092(-0.245~0.061) | 0.240 |
| 3.0 | -0.114(-0.272~0.045) | 0.160 |  | -0.138(-0.329~0.053) | 0.157 |  | -0.119(-0.277~0.037) | 0.133 |  | -0.075(-0.211~0.061) | 0.280 |

^a^ Bootstrap sampling and estimation using Stata 15.1, performing 1000 bootstrap replications.

^b^ Estimations were adjusted for maternal age, body mass index, gravidity, parity, and TG.

Table 17 Adjusted Odds Ratios (aOR) with 95% Confidence Intervals (CI) for Gestational Diabetes Mellitus (GDM) associated with rs8192678 and rs2971672 genotypes in validation analyses ^a^

|  | β | SE | Z | aOR with 95% CI ^b^ | P |
| --- | --- | --- | --- | --- | --- |
| rs2971672（A>C） |  |  |  |  |  |
| AC vs AA | -0.361 | 0.244 | -1.48 | 0.697 (0.427~1.137) | 0.148 |
| CC vs AA | -0.753 | 0.308 | -2.44 | 0.471 (0.257~0.863) | 0.015 |
| AC+CC vs AA | -0.484 | 0.227 | -2.13 | 0.616 (0.392~0.967) | 0.036 |
| CC vs AC+AA | 0.578 | 0.161 | -1.96 | 0.578 (0.329~1.016) | 0.057 |
| rs8192678（C>T） |  |  |  |  |  |
| CT vs CC | -0.390 | 0.245 | -1.59 | 0.677 (0.411~1.117) | 0.106 |
| TT vs CC | -0.874 | 0.327 | -2.67 | 0.417 (0.220~0.790) | 0.007 |
| CT+TT vs CC | -0.522 | 0.231 | -2.26 | 0.593 (0.376~0.935) | 0.025 |
| TT vs CT+CC | -0.647 | 0.288 | -2.24 | 0.524 (0.198~0.919) | 0.024 |

^a^ Bootstrap sampling and estimation using Stata 15.1, performing 1000 bootstrap replications.

^b^ Estimations were adjusted for maternal age, body mass index, gravidity, parity, and TG.
