# Supplementary material for: Genetic susceptibility to gestational diabetes and its mild modification by bisphenol A and thyroid-stimulating hormone: findings from a South Chinese pregnancy cohort
Source: Front Nutr. 2025 Dec 10;12:1652265. doi: 10.3389/fnut.2025.1652265 (PMC12729077; doi:10.3389/fnut.2025.1652265)
Supplement: Supplementary file 3 [file Presentation_1.PDF]

Supplemental Methods and Results

Methods

2.2 Whole exome sequencing (WES)

Genomic DNA was extracted from whole blood samples using a DNA extraction kit (Qiagen, Hilden, Germany), according to the manufacturer’s instructions. DNA was stored at −80 °C until further processing. Following the manufacturer's protocol, 200 ng of genomic DNA from each individual was sheared by Biorupter (Diagenode, Belgium) to acquire 150–200 bp fragments. The ends of the DNA fragments were repaired, and Illumina Adapter was added (Fast Library Prep Kit, iGeneTech, Beijing, China). Then, libraries were constructed, and the whole exomes were captured using the AIExome Enrichment Kit V1 (iGeneTech,Beijing, China) and sequenced on an Illumina NovaSeq 6000 (Illumina, San Diego, CA) next generation sequencing platform, with 150 paired-end reads.

WES data were analyzed using the standard pipeline. First, the raw reads were filtered to remove low-quality reads using FastQC. Clean reads were then mapped to the reference genome GRCh37 (hg19) using Burrows-Wheeler alignment (BWA). After removing duplications, single nucleotide variants (SNVs) and indels were identified using the Genome Analysis Toolkit (GATK), and the intersection of the mutation sets of all patients was analyzed.

2.2.1 Identification of rare potentially-pathogenic variants

The minor allele frequencies of the most promising candidate pathogenic variants were screened using public databases, including 1000 Genomes Project (http://www.1000genomes.org), Exome Aggregation Consortium (ExAC)

(http://exac.broadinstitute.org), and the Genome Aggregation Database (gnomAD).

The significant SNVs (P<0.05) were validated by leave-one-out cross-validation (LOOCV), k-fold cross-validation, and random subset validation. The agreement of p-values between the Chi^2 and Fisher's test was checked, and subsequently, stability scores were calculated for each variant: Highly stable (>=0.8) , Moderately Stable (0.6-0.8), Partially Stable (0.4-0.6), Unstable (<0.4).

The significant SNVs (P<0.05) were classified according to damaging or probably damaging prediction. Predictive software tools were listed: SIFT (http://sift.jcvi.org/), Polyphen-2 (http://genetics.bwh.harvard.edu/pph2/), MutationAssessor, MutationTaster (http://www.mutationtaster.org/), LRT, FATHMM, fathmm-MKL, PROVEAN (http://provean.jcvi.org/index.php), MetaSVM, MetaLR, M-CAP and Combined Annotation Dependent Depletion (cadd.gs.washington.edu). The mutations were categorized into four levels: high、likeyhigh、medium and low. High (within the exonic region; not within repeat region; at least one MAF was less than 0.01 by 1000g, ExAC, gnomAD; at least one software prediction result was damaging); Likely high (within the exonic or splicing region; not within repeat region; at least one MAF was less than 0.01 by 1000g, ExAC, gnomAD); Medium (within the exonic or splicing region; not within repeat region; all MAFs were at least 0.01 by 1000g, ExAC, gnomAD; at least one software prediction result was damaging); Low represents all the other variants.

2.3 Genotyping

Genomic DNA was extracted from whole blood using a DNA extraction kit (Qiagen, Hilden, Germany), following the manufacturer’s instructions.

DNA was stored at −80 °C until further processing. Two microliters of DNA from each sample were used for genotyping. All SNVs were genotyped using the Sequenom MassArray platform, which involved polymerase chain reaction (PCR), shrimp alkaline phosphatase (SAP) reaction, single base extension, resin cleanup, and detection by mass spectrometry. The gene mass spectrogram was obtained using matrix-assisted laser desorption ionization time-of-flight mass spectrometry.

2.4 Statistical analysis

In the validation stage, we used the SHEsisPlus platform for the analyses of Hardy–Weinberg equilibrium (HWE), linkage disequilibrium (LD), and comparisons of genotype and allele frequencies [1, 2]. Results were expressed as odds ratios (ORs) with 95% confidence intervals (95% CI).

Functional enrichment analysis and Protein–protein enrichment analysis was performed using a online tool Metascape (https://metascape.org/)[3]. A gene list of 246 genes for 308 missense variants were first converted into their corresponding H. sapiens Entrez gene IDs using the latest version of the database (last updated on 2024-05-01). If multiple identifiers correspond to the same Entrez gene ID, they will be considered as a single Entrez gene ID in downstream analyses. Pathway and process enrichment analysis were carried out with the following ontology sources: GO Biological Processes, GO Cellular Components, GO Molecular Functions, KEGG Pathway, and Immunologic Signatures. All genes in the genome have been used as the enrichment background. Terms with a p-value < 0.01, a minimum count of 3, and an enrichment factor > 1.5 (the enrichment factor is the ratio between the observed counts and the counts expected by chance) were collected and grouped into clusters based on

their membership similarities. More specifically, p-values are calculated based on the cumulative hypergeometric distribution [4], and q-values are calculated using the Benjamini-Hochberg procedure to account for multiple testings [5]. Kappa scores [6] are used as the similarity metric when performing hierarchical clustering on the enriched terms, and sub-trees with a similarity of > 0.3 are considered a cluster. The most statistically significant term within a cluster is chosen to represent the cluster.

Protein–protein enrichment analysis has been carried out with the following databases: STRING [7], BioGrid [8], OmniPath [9], InWeb_IM [10].Only physical interactions in STRING (physical score > 0.132) and BioGrid are used (https://metascape.org/blog/?p=219). The resultant network contains the subset of proteins that form physical interactions with at least one other member in the list. If the network contains between 3 and 500 proteins, the Molecular Complex Detection (MCODE) algorithm [11] has been applied to identify densely connected network components. The MCODE networks identified for individual gene lists have been gathered and are shown in Figure 1(c). Pathway and process enrichment analysis has been applied to each MCODE component independently, and the three best-scoring terms by p-value have been retained as the functional description of the corresponding components, shown in Figure 1(c).

The prediction of structural change was performed using the online tool Hope (https://www3.cmbi.umcn.nl/hope/) and SWISS-MODEL (https://swissmodel.expasy.org/) [12,13]. The alteration mode of proteins of the significant genes was visualized using PyMOL (http://www.pymol.org) [14].

The phase separation (PS) of the selected proteins was predicted by PhaSePred

(predict.phasep.pro). This online tool incorporates residue-level scores of several PS predictors and PS-related features [15].

2.5 Power calculation

In this study, we assumed that the allele frequency of PPARGC1A rs8192678 (C>T) or GCK rs2971672 (A>C) in the control group was 0.52. We were going to obtain a sample of 185 patients, along with one controls for each patient. This resulted in a sample of 370 patients achieving detection powers of 98.85%, 90.06%, 67.47%, 39.58%, and 18.68% for odds ratios of 0.4, 0.5, 0.6, 0.7, and 0.8, respectively, against the null hypothesis of equal odds using two-side Z test at a 0.05 significance level.

A logistic regression of a binary response variable (Y: GDM) on a binary independent variable (X: rs8192678 （C>T）) with a sample size of 372 observations (of which 18.01% are in the group X=TT) achieves 88.70% power using an Z-test with a significance level (α) of 0.05 to detect a change in Prob(Y=GDM) . This change corresponds to an odds ratio of 0.417. An adjustment was made since a multiple regression of the independent variable of interest on the other independent variables in the logistic regression obtained an R-Squared of 0.0238. The power is 74.4% to detect a change in Prob(Y=GDM) corresponding to an odds ratio of 0.595 with a sample size of 372 observations (of which 66.40% are in the group X=CT+TT). For rs2971672 (A>C), the power is 84.47% to detect a change in Prob(Y=GDM) corresponding to an odds ratio of 0.47 with a sample size of 371 observations (of which 20.49% are in the group X=CC) and is 69.38% corresponding to an odds ratio of 0.616 with a sample size of 371 observations (of which 65.77% are in the group X=AC+CC).

For sub-populations, a logistic regression of a binary response variable (Y: GDM) on a

continuous, normally distributed variable (X: BPA) with a sample size of 152 observations achieves 97.54% power at a significance level of 0.05 to detect a change in Prob(Y=1) from 0.4517 at the mean of X to 0.654 when X is increased by one standard deviation. This change corresponds to an odds ratio of 2.295. An adjustment was made due to a multiple regression of the independent variable of interest on the other independent variables in the logistic regression, which obtained an R-Squared of 0.3903. The power is 99.4% to detect a change in Prob(Y=1) from 0.4522 at the mean of X to 0.6577 when X is increased by one standard deviation, corresponding to an odds ratio of 2.328, with an R-Squared of 0.2546 for the other independent variables. The power is 93.86% to detect a change in Prob(Y=1) from 0.4505 at the mean of X to 0.6386 when X is increased by one standard deviation, corresponding to an odds ratio of 2.156, with an R-Squared of 0.446 for the other independent variables.

Results

3.4 Enrichment and Protein-protein interaction network construction

The online tool Metascape identified 54 enriched biological processes, 19 enriched molecular function clusters, 13 enriched cellular components, and four enriched KEGG pathways. We keep the GO terms of which the best log P <=-2 (Table S5). The top-ranked biological processes with the smallest P-values showed that these 246 genes were strongly related to various aspects of cilium movement involved in cell motility, cilium or flagellum-dependent cell motility and cilium-dependent cell motility, cilium movement, cilium assembly, cilium organization, all of which are related to cilium-dependent cell motility. The top molecular function clusters showed that these differentially expressed genes between cases and controls were strongly related to functions involving structural molecule activity, ATP-dependent

activity and protein binding, such as extracellular matrix constituent, lubricant activity, minus-end-directed microtubule motor activity, dynein intermediate chain binding. The cell component GO category showed enrichment predominantly for Golgi lumen, dynein complex and cilium. The two most highly enriched classes of KEGG pathways were olfactory transduction, with 13 genes identified (OR6A2, CALML5, OR51A7, OR4D11, OR10A4, OR10A2, OR56B1, OR52N5, OR52N2, OR8B3, OR11G2, OR51A2, OR2T27), and fanconi anemia pathway, with 4 genes defined (FANCE, PMS2, TELO2, POLN).

3.5 Validation and Linkage disequilibrium analysis

When analyzing the LD patterns of 62 SNVs, we identified that MAGEB16 rs5973488 with rs1410961, NFKB1 rs72696119 with rs17032850, TCF7L2 rs11196218 with rs6585205, ADIPOQ rs822393 with rs182052, CDKN2B rs10965250 with CDKN2A/B rs10811661, CDKAL1 rs7756992 with rs7747752, LPIN2 rs767620772 with FANCA rs778093769 IFT140 rs775044452 with COBLL1 rs764256858 showed strong relationships with both D' > 0.8 and R^2 >0.33 (Figure S3). The haplotype analysis of the sixteen variants were presented in Table S9. Moreover, the gene interaction P values are shown in Table S10. KCNJ1 rs117535913 with IGF2BP2 rs4402960, ADIPOQ rs822393 with rs1501299, APOA2 rs6413453 with MAGEB16 rs1410961, ADIPOQ rs16861194 with PPARGC1B 138772212, MAGEB16 rs5973488 with APOA2 rs6413453, GPI rs8191371 with GCK rs2971672, SLC16A11 rs13342232 with LRBA rs776254567, SLC16A11 rs13342232 with DIO2 rs225014, TCF7L2 rs7895340 with CDKAL1 rs7747752 showed strong relationships with P < 0.01.

1. Shi YY, He L: SHEsis, a powerful software platform for analyses of linkage disequilibrium, haplotype construction, and genetic association at polymorphism loci. *Cell research* 2005, 15(2):97-98.
2. Li Z, Zhang Z, He Z, Tang W, Li T, Zeng Z, He L, Shi Y: A partition-ligation-combination-subdivision EM algorithm for haplotype inference with multiallelic markers: update of the SHEsis (http://analysis.bio-x.cn). *Cell research* 2009, 19(4):519-523.
3. Zhou et al., Metascape provides a biologist-oriented resource for the analysis of systems-level datasets. Nature Communications (2019) 10(1):1523.
4. Zar, J.H. Biostatistical Analysis 1999 4th edn., NJ Prentice Hall, pp. 523
5. Hochberg Y., Benjamini Y. More powerful procedures for multiple significance testing. Statistics in Medicine (1990) 9:811-818.
6. Cohen, J. A coefficient of agreement for nominal scales. Educ. Psychol. Meas. (1960) 20:27-46.
7. Szklarczyk D. et al. STRING v11: protein-protein association networks with increased coverage, supporting functional discovery in genome-wide experimental datasets. Nucleic Acids Res. (2019) 47:D607-613.
8. Stark C. et al. BioGRID: a general repository for interaction datasets. Nucleic Acids Res. (2006) 34:D535-539.
9. Turei D. et al. A scored human protein-protein interaction network to catalyze genomic interpretation. Nat. Methods. (2016) 13:966-967.
10. Li T. et al. A scored human protein-protein interaction network to catalyze genomic interpretation. Nat. Methods. (2017) 14:61-64.
11. Bader, G.D. et al. An automated method for finding molecular complexes in large protein interaction networks. BMC bioinformatics (2003) 4:2.
12. Venselaar H, te Beek TAH, Kuipers RKP, Hekkelman ML, Vriend G: Protein structure analysis of mutations causing inheritable diseases. An e-Science approach with life scientist friendly interfaces. *BMC Bioinformatics* 2010, 11(1):548.
13. Waterhouse A, Bertoni M, Bienert S, Studer G, Tauriello G, Gumienny R, Heer FT, de Beer TAP, Rempfer C, Bordoli L, Lepore R, Schwede T SWISS-MODEL: homology modelling of protein structures and complexes.Nucleic Acids Res 46, W296-W303. (2018)
14. Venselaar H, te Beek TAH, Kuipers RKP, Hekkelman ML, Vriend G: Protein structure analysis of mutations causing inheritable diseases. An e-Science approach with life scientist friendly interfaces. BMC Bioinformatics 2010, 11(1):548.
15. Chen Z, Hou C, Wang L, Yu C, Chen T, Shen B, Hou Y, Li P, Li T. Screening membraneless organelle participants with machine-learning models that integrate multimodal features. Proc Natl Acad Sci U S A. 2022 Jun 14;119(24):e2115369119.
